# Supplementary figures and images for: Type I and II PRMTs inversely regulate post-transcriptional intron detention through Sm and CHTOP methylation
Source: eLife. 2022 Jan 5;11:e72867. doi: 10.7554/eLife.72867 (PMC8765754; doi:10.7554/eLife.72867)

c

Chemiluminescence

Digital

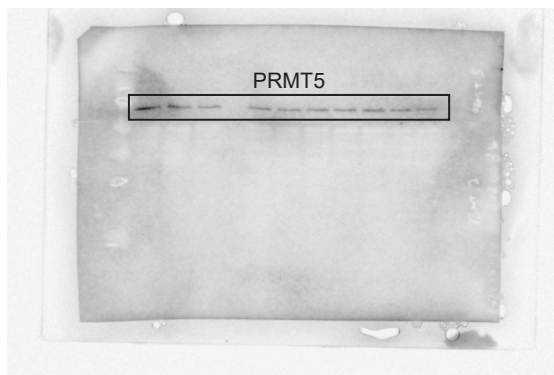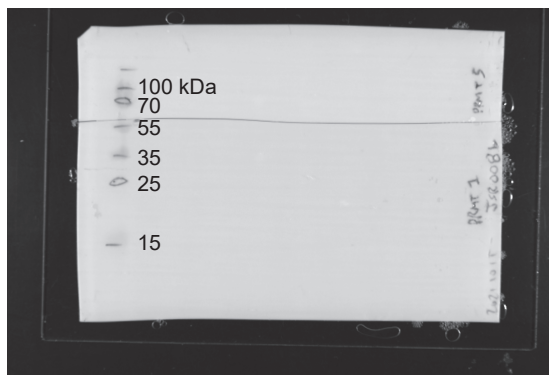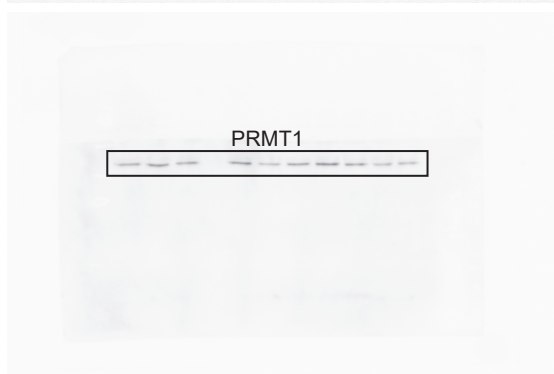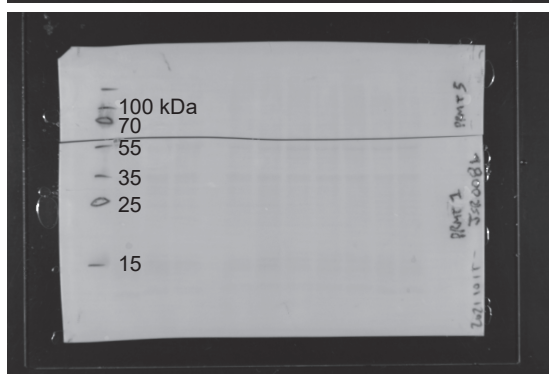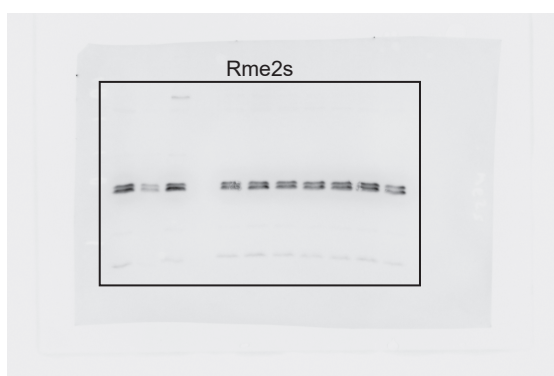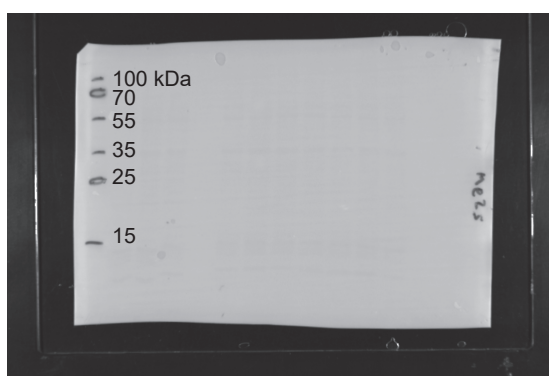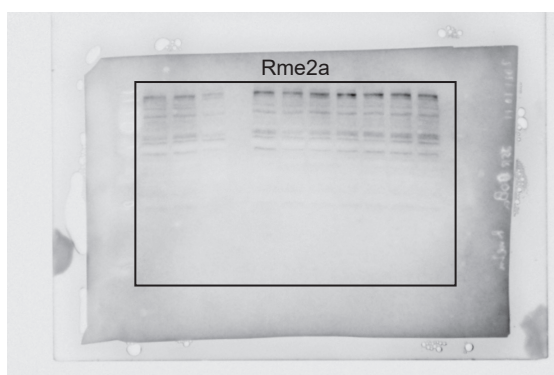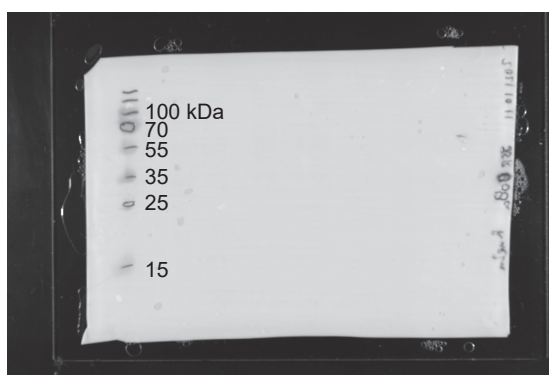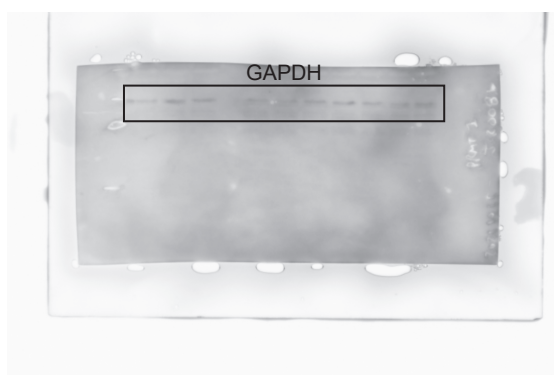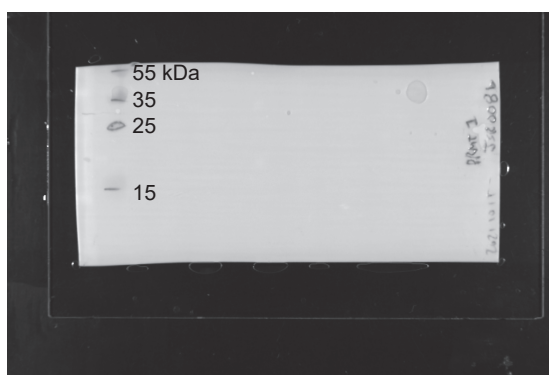

Supplement: Figure 1—figure supplement 1—source data 1. [file elife-72867-fig1-figsupp1-data1.zip › Figure1-figure_supplement_1-source data1/Figure 1-Figure Supplement 1-source data 1.pdf]

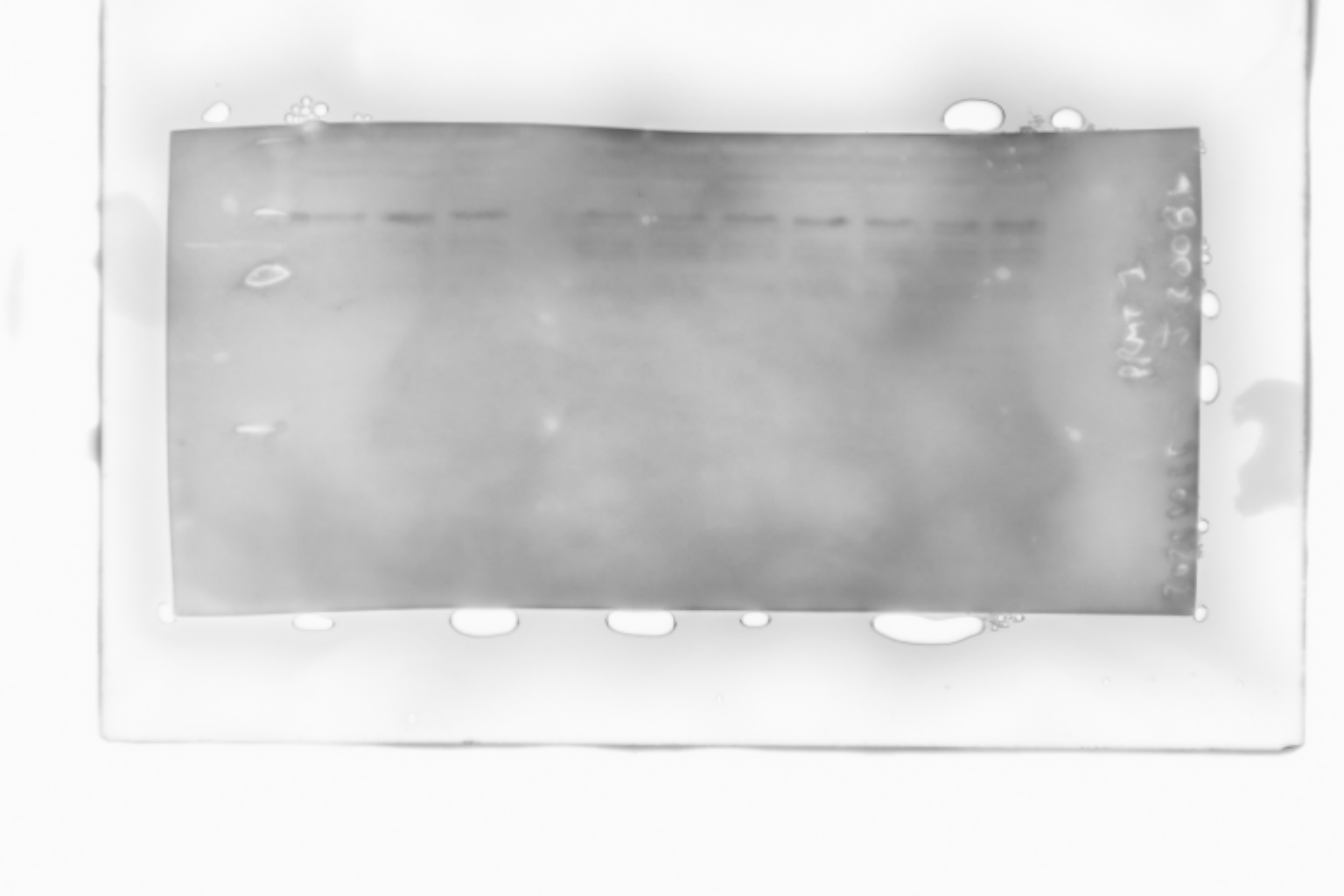

Supplement: Figure 1—figure supplement 1—source data 1. [file elife-72867-fig1-figsupp1-data1.zip › Figure1-figure_supplement_1-source data1/PRMTKnockDown_GAPDH_1.10000_High_30sec_10.tif]

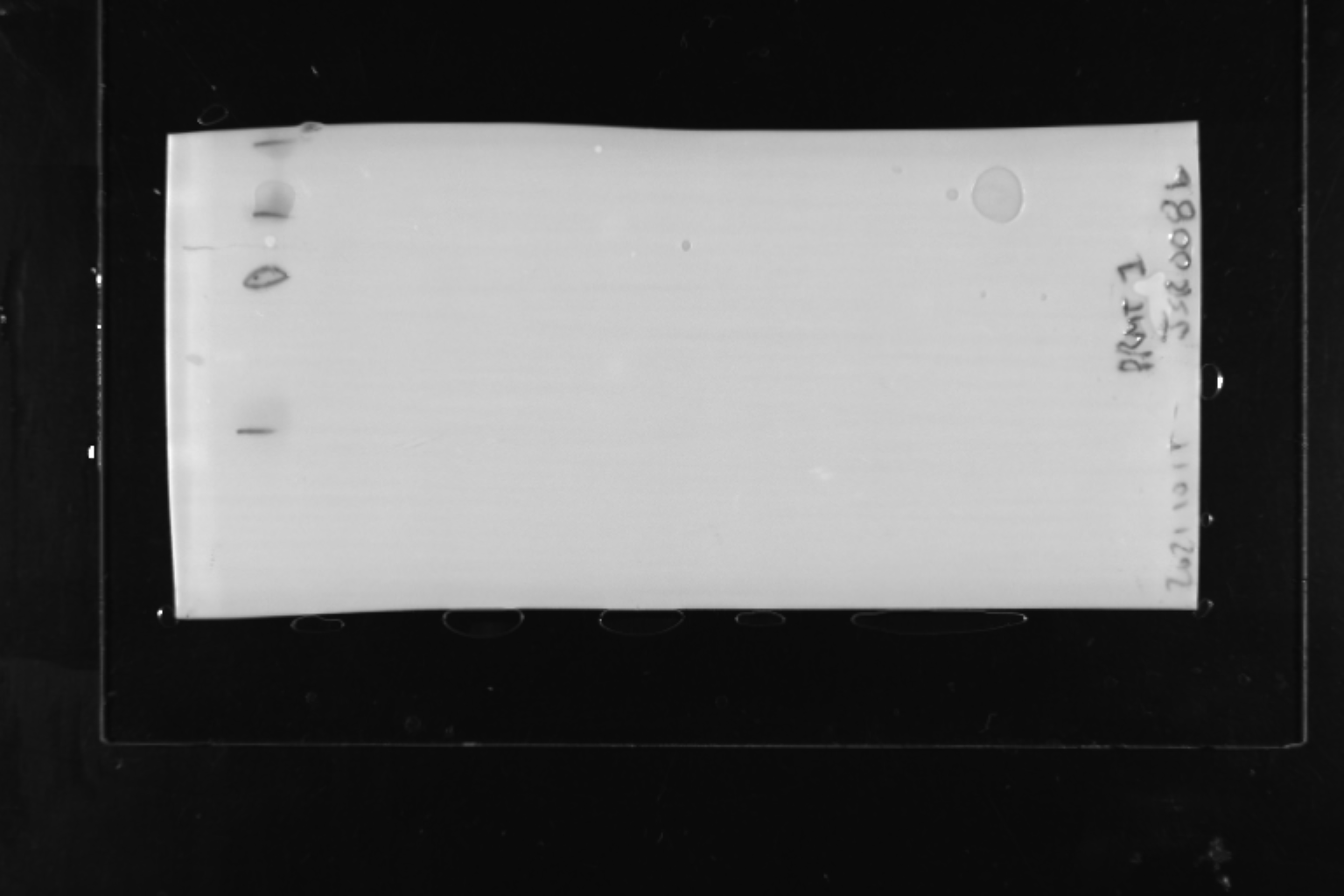

Supplement: Figure 1—figure supplement 1—source data 1. [file elife-72867-fig1-figsupp1-data1.zip › Figure1-figure_supplement_1-source data1/PRMTKnockDown_GAPDH_1.10000_High_digital.tif]

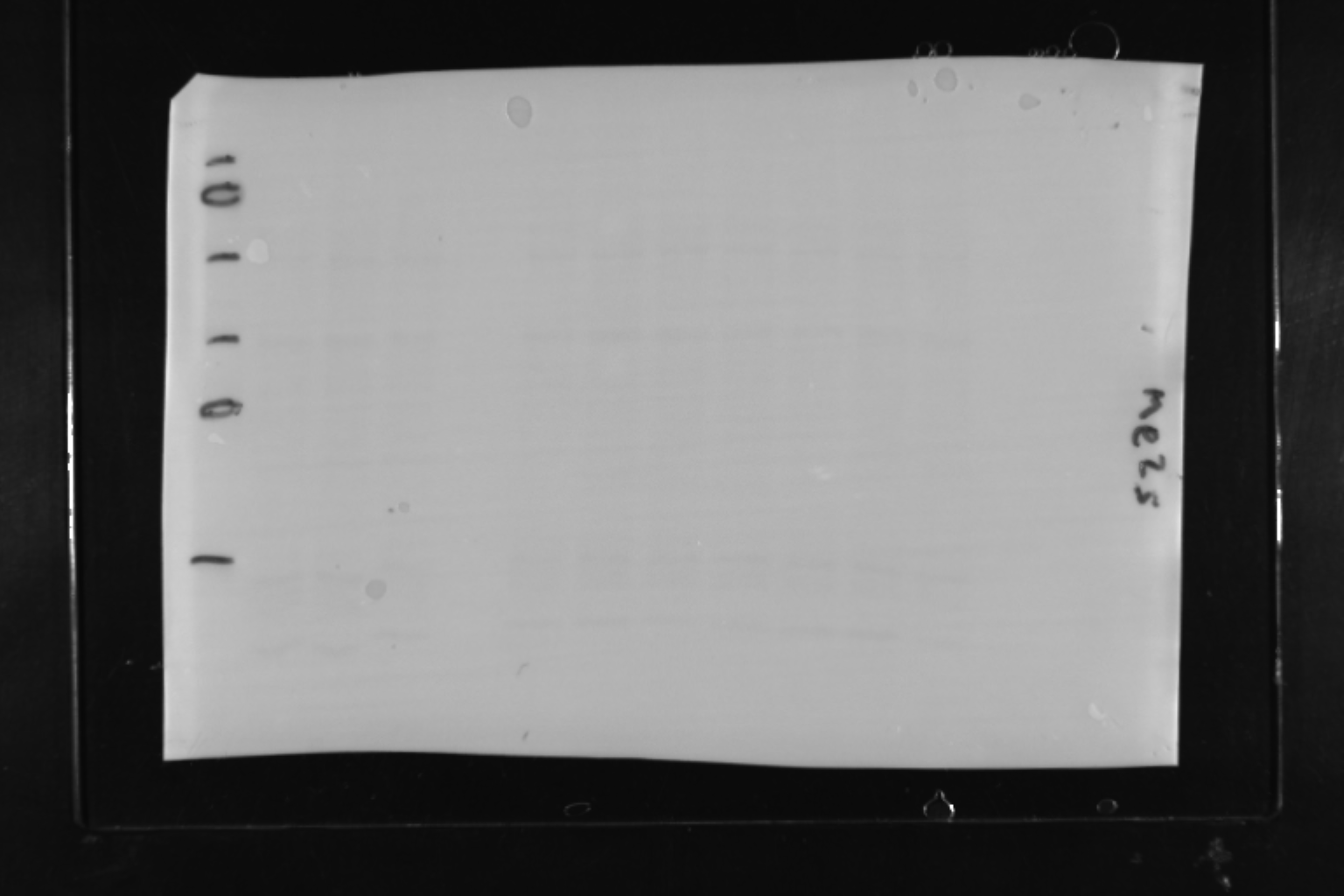

Supplement: Figure 1—figure supplement 1—source data 1. [file elife-72867-fig1-figsupp1-data1.zip › Figure1-figure_supplement_1-source data1/PRMTKnockDown_Me2s_1.2000_digital.tif]

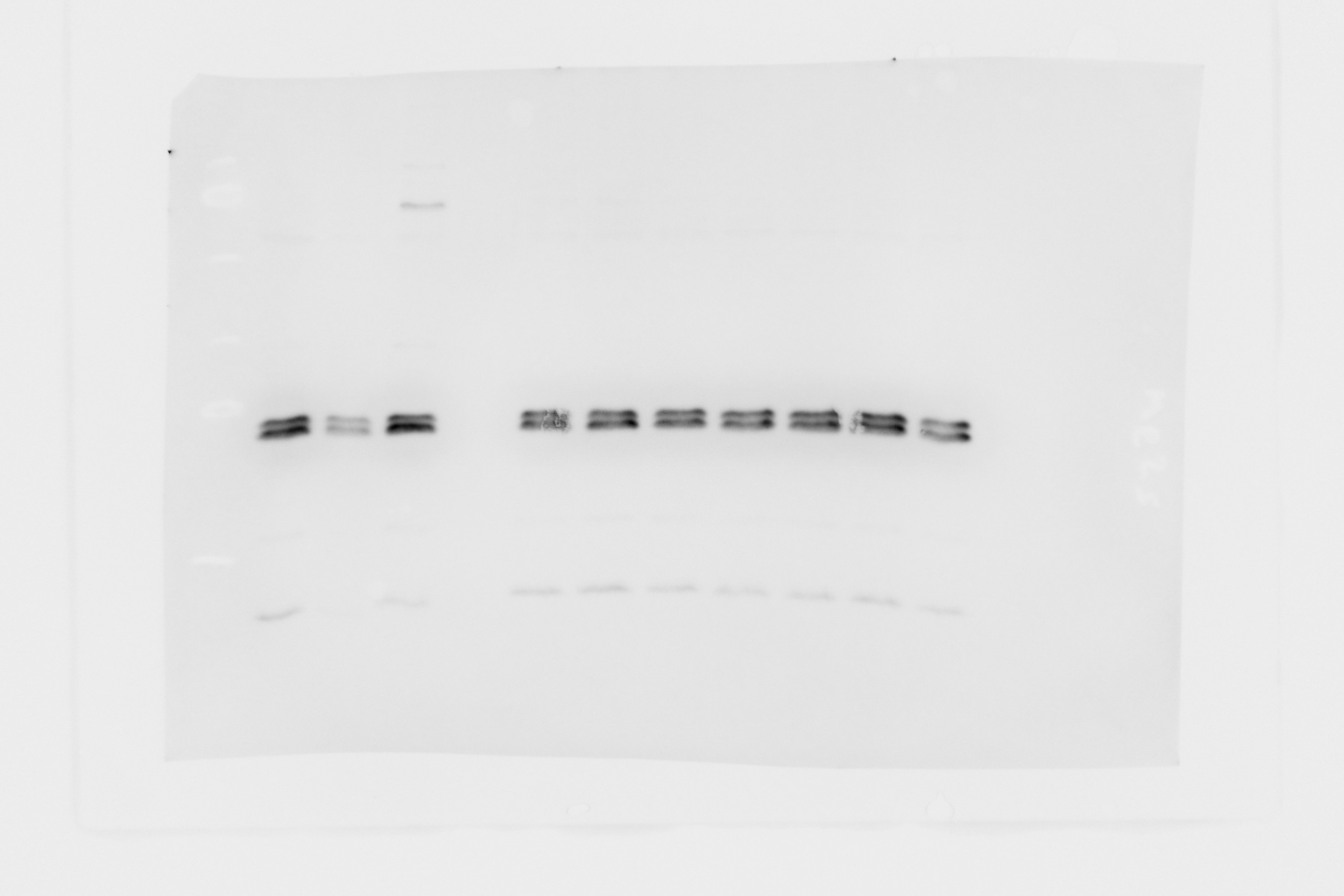

Supplement: Figure 1—figure supplement 1—source data 1. [file elife-72867-fig1-figsupp1-data1.zip › Figure1-figure_supplement_1-source data1/PRMTKnockDown_Me2s_1.2000_StandardRes_30sec_5.tif]

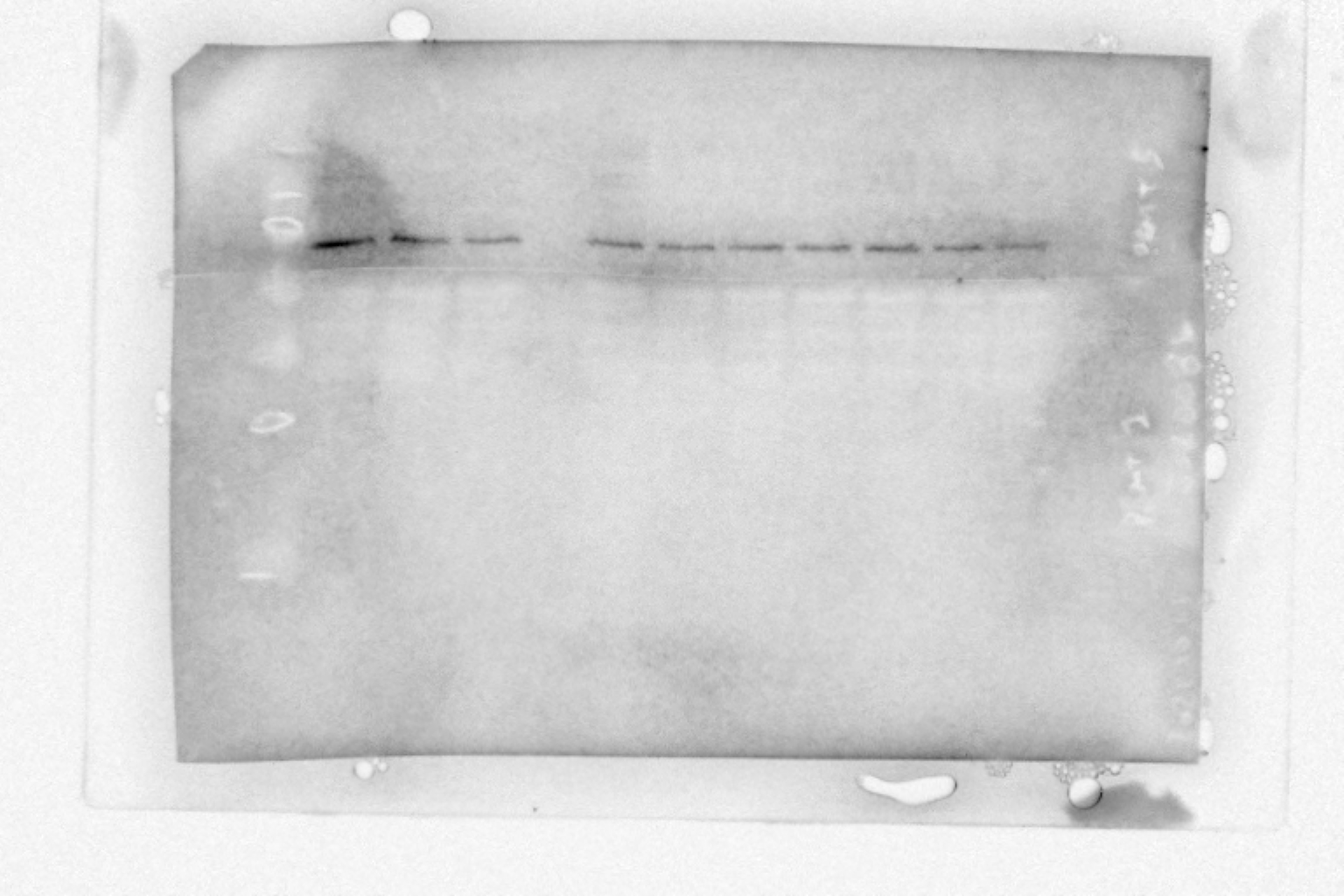

Supplement: Figure 1—figure supplement 1—source data 1. [file elife-72867-fig1-figsupp1-data1.zip › Figure1-figure_supplement_1-source data1/PRMTKnockDown_PRMT5_1.5000_High_30sec_10.tif]

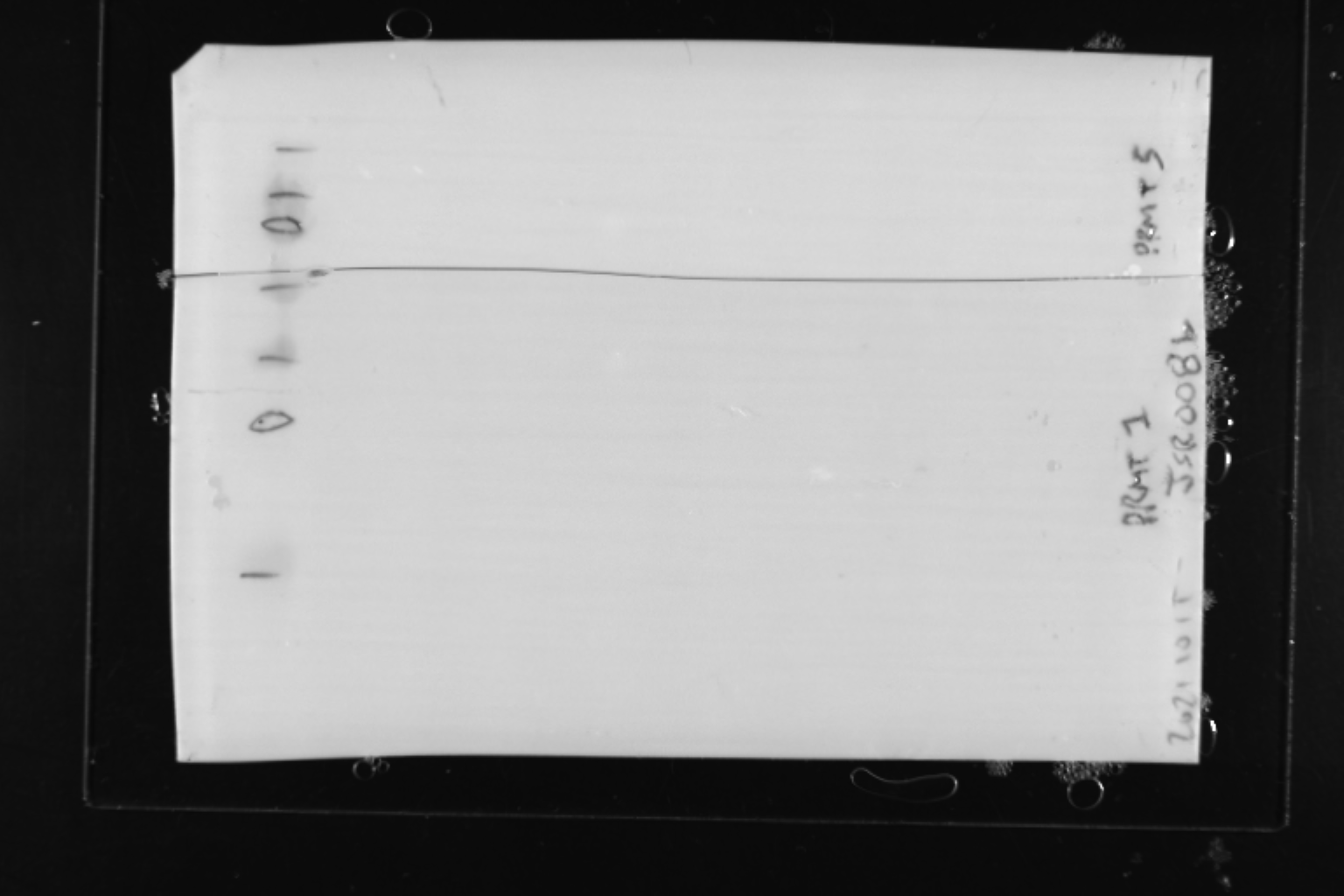

Supplement: Figure 1—figure supplement 1—source data 1. [file elife-72867-fig1-figsupp1-data1.zip › Figure1-figure_supplement_1-source data1/PRMTKnockDown_PRMT5_1.5000_High_digital.tif]

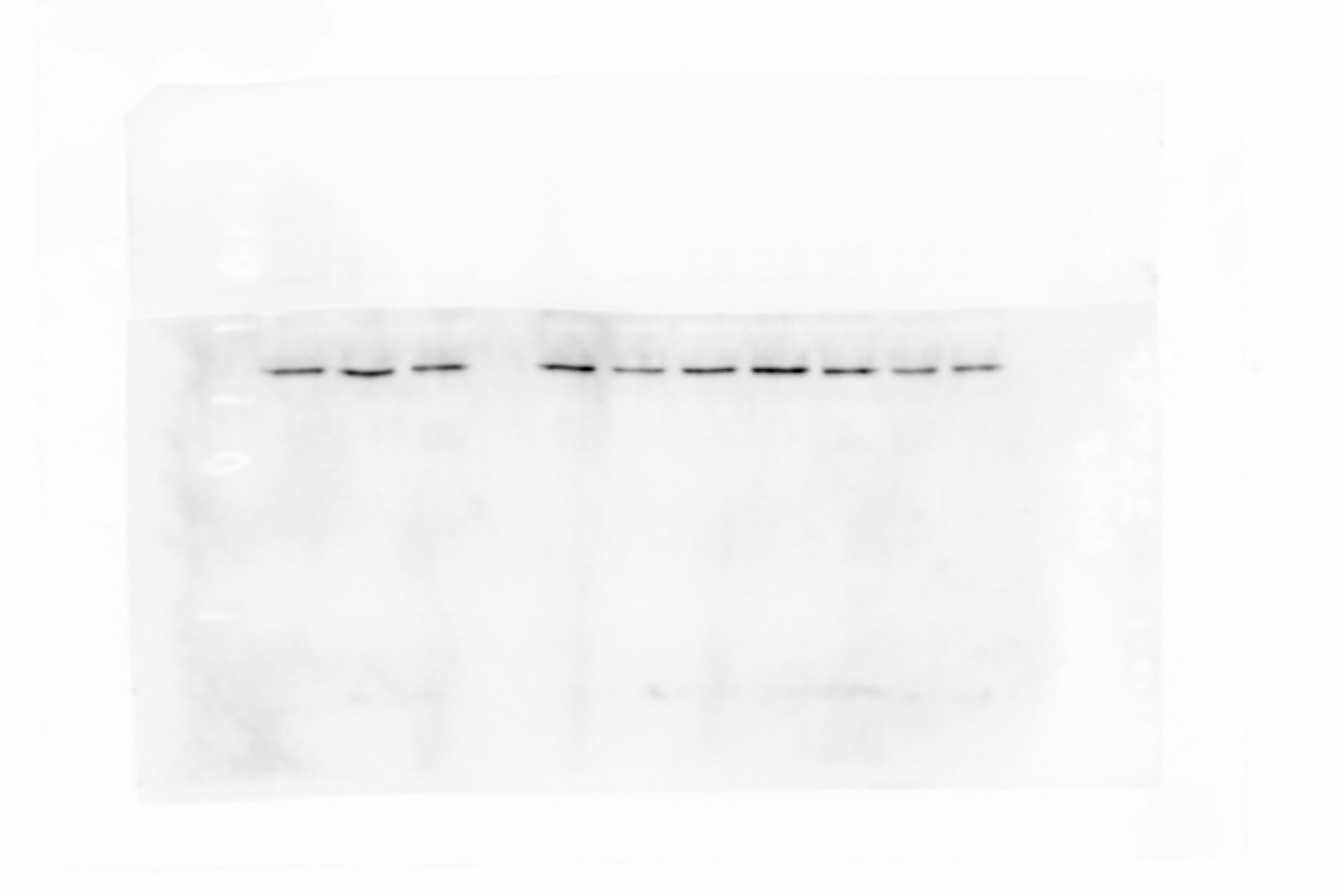

Supplement: Figure 1—figure supplement 1—source data 1. [file elife-72867-fig1-figsupp1-data1.zip › Figure1-figure_supplement_1-source data1/PRMTKnockDown_PRMT5_1.5000_PRMT1_1.5000_High_30sec_9.tif]

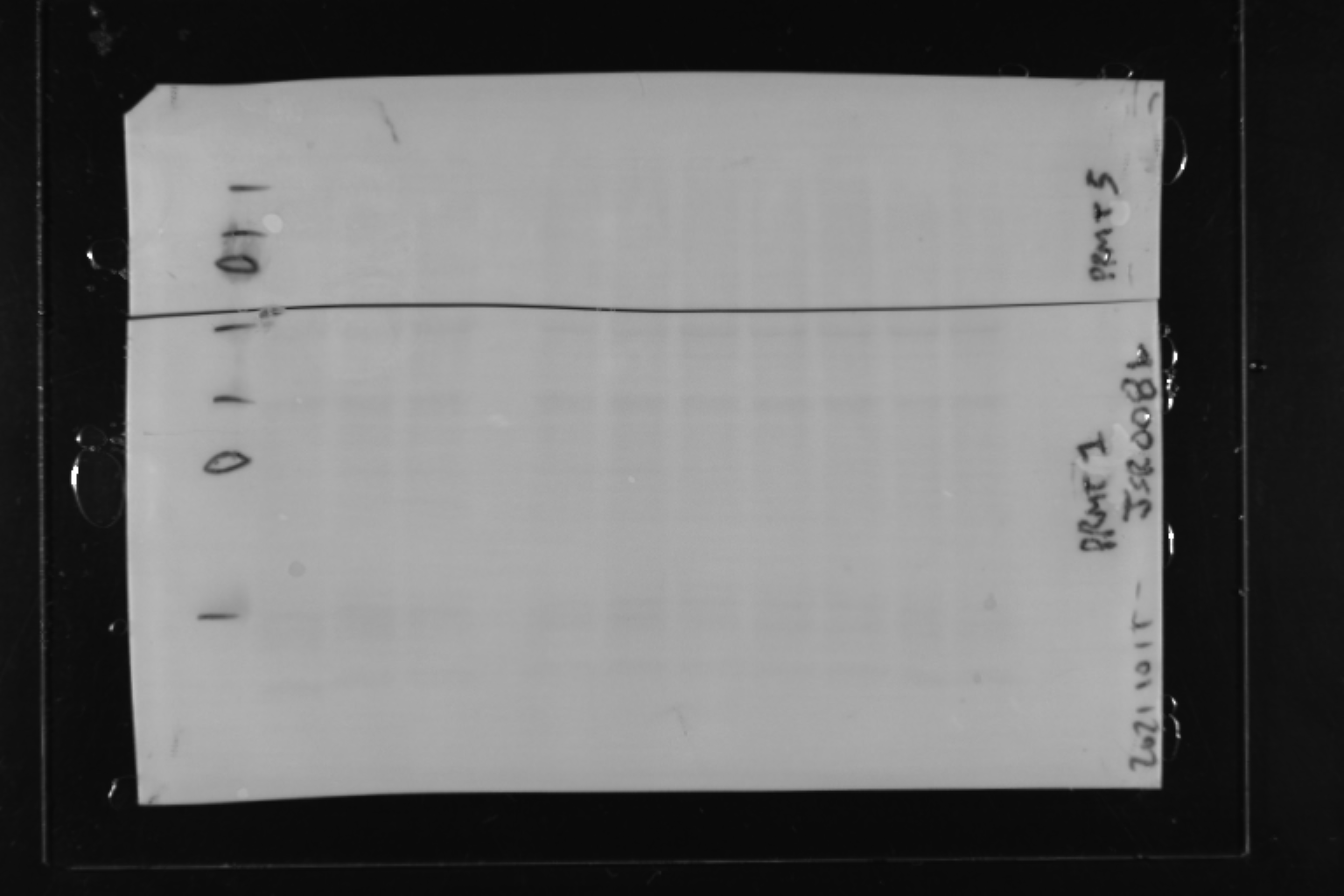

Supplement: Figure 1—figure supplement 1—source data 1. [file elife-72867-fig1-figsupp1-data1.zip › Figure1-figure_supplement_1-source data1/PRMTKnockDown_PRMT5_1.5000_PRMT1_1.5000_High_digital.tif]

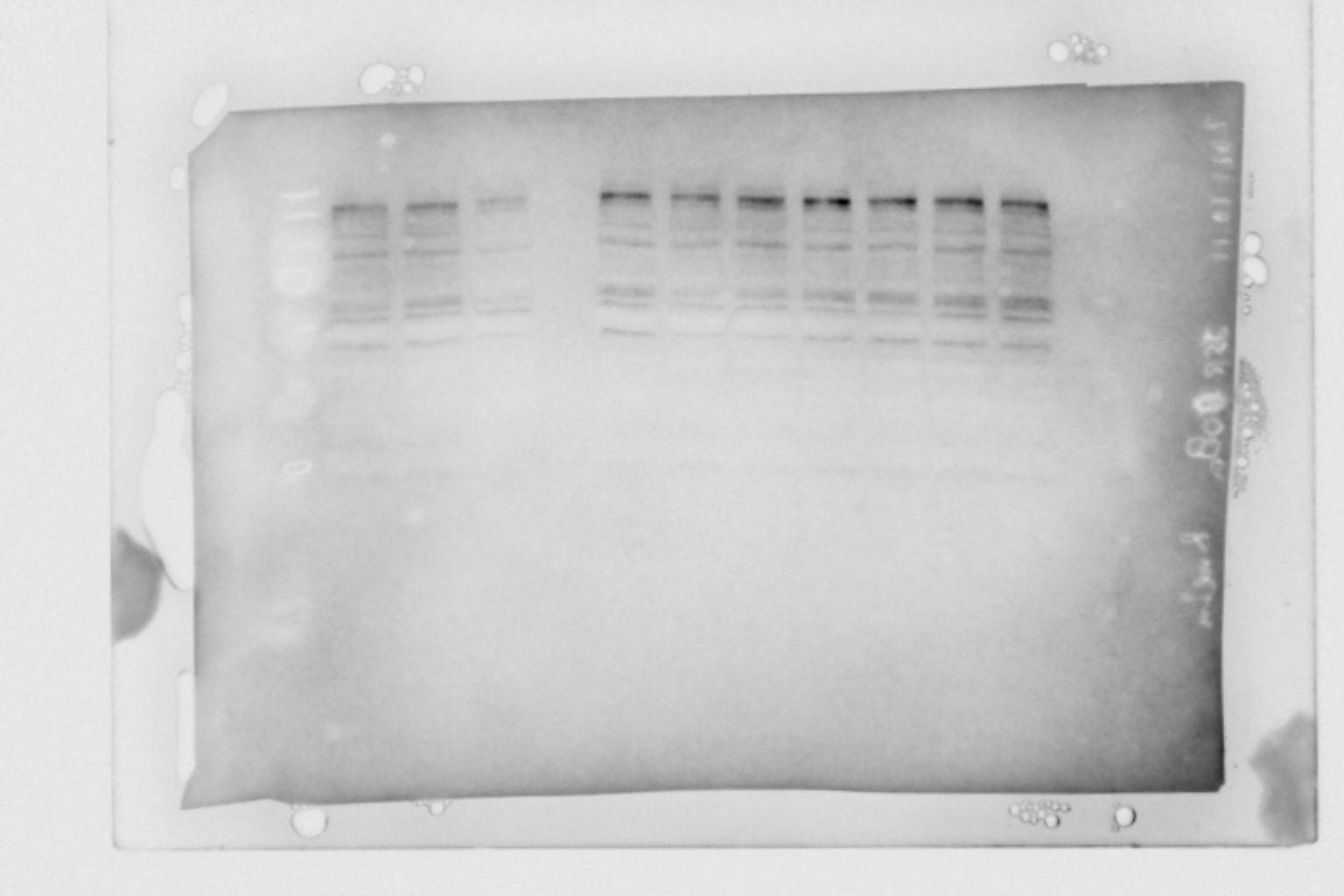

Supplement: Figure 1—figure supplement 1—source data 1. [file elife-72867-fig1-figsupp1-data1.zip › Figure1-figure_supplement_1-source data1/PRMTKnockDown_RMe2a_1.2000_High_30sec_10.tif]

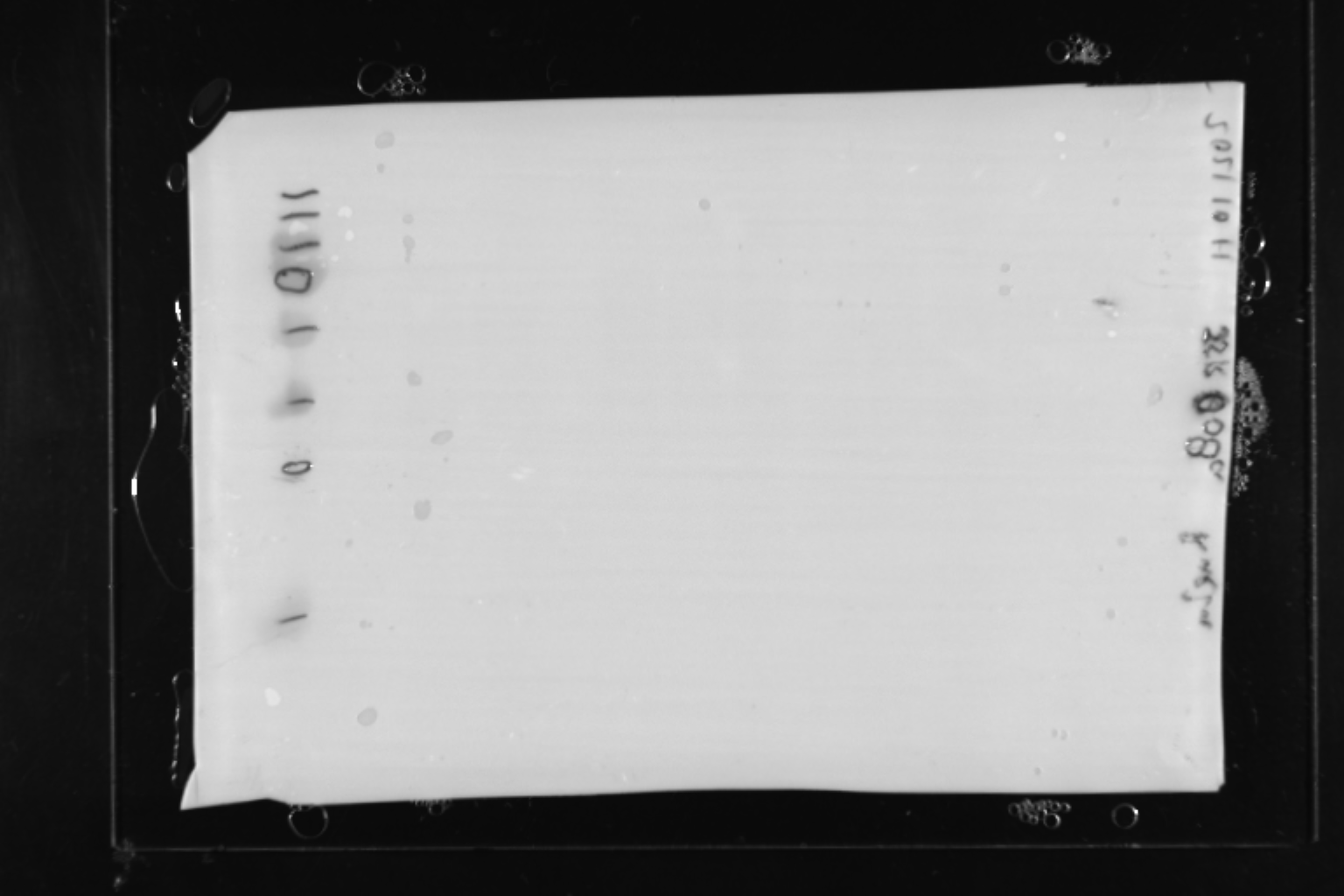

Supplement: Figure 1—figure supplement 1—source data 1. [file elife-72867-fig1-figsupp1-data1.zip › Figure1-figure_supplement_1-source data1/PRMTKnockDown_RMe2a_1.2000_High_digital.tif]

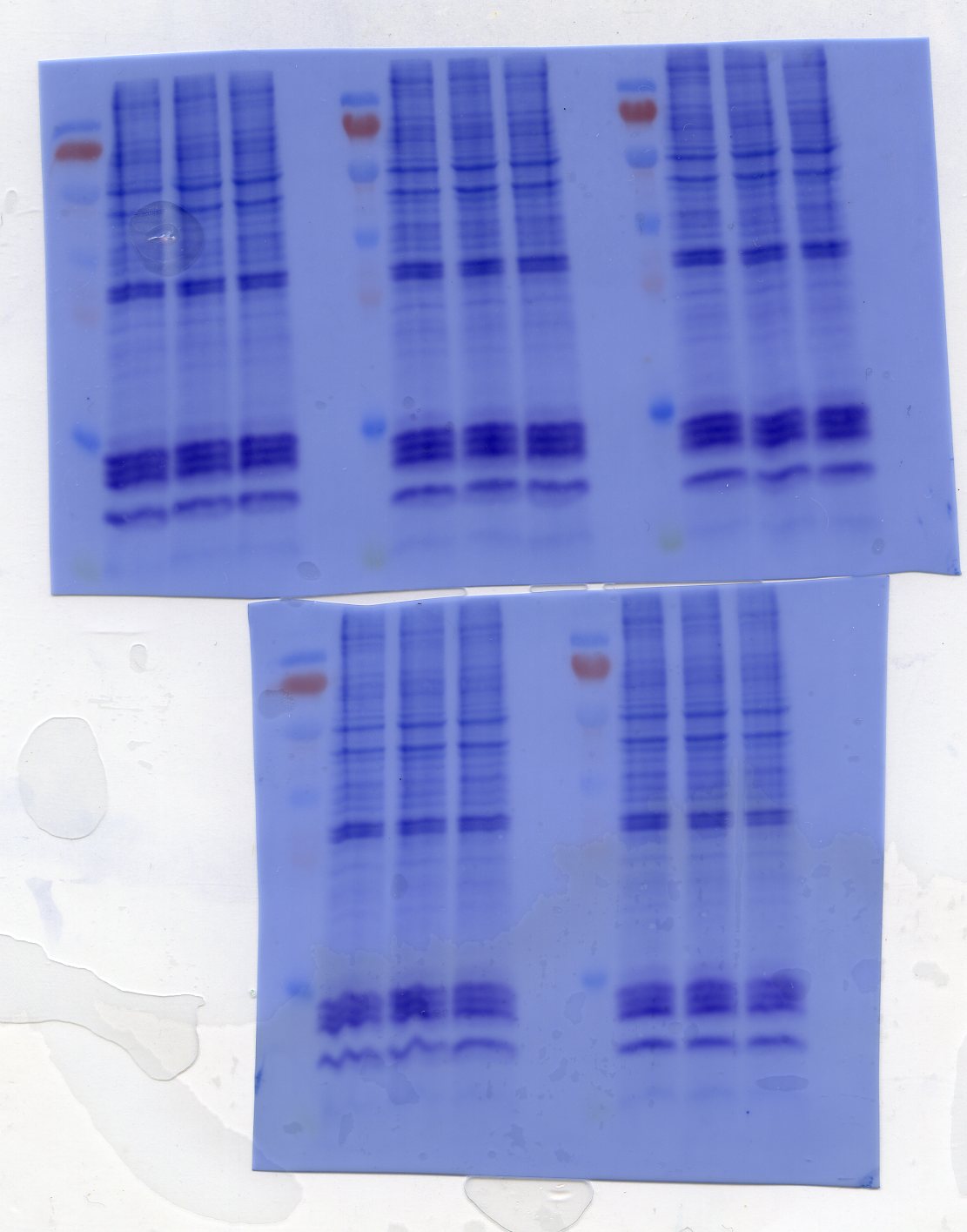

Supplement: Figure 1—figure supplement 1—source data 2. [file elife-72867-fig1-figsupp1-data2.zip › Figure1-figure_supplement_1-source data2/042921_A549_48h_PRMTi_chromatin.jpg]

d

Chemiluminescence

Digital

Direct Blue 71 Membrane Stain

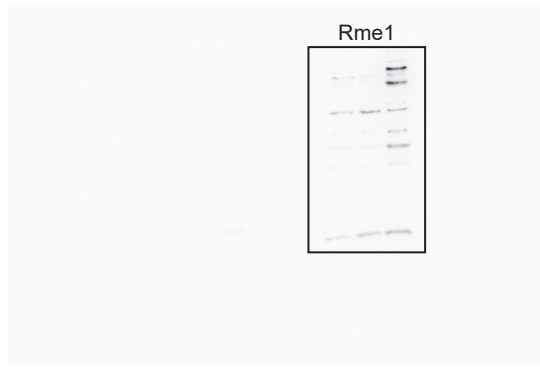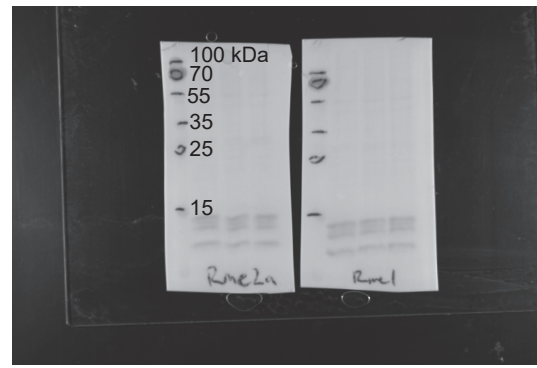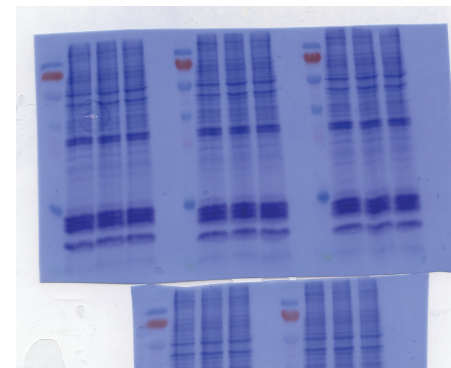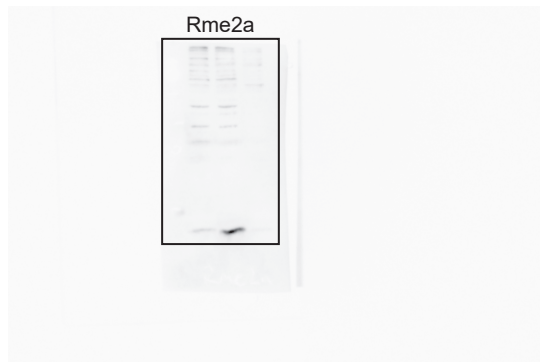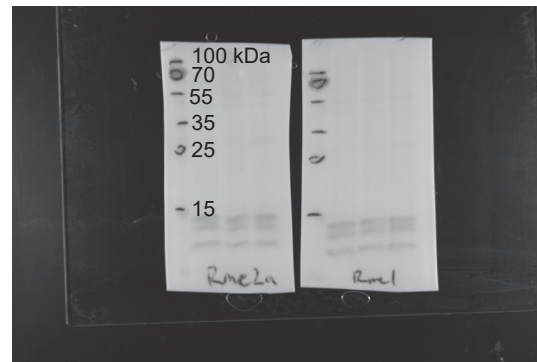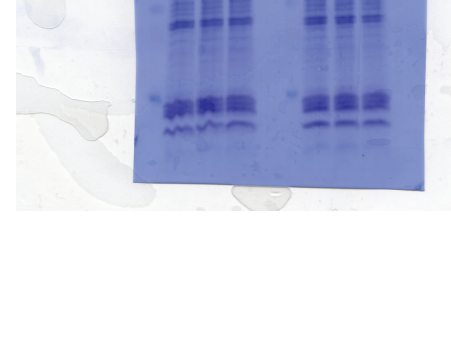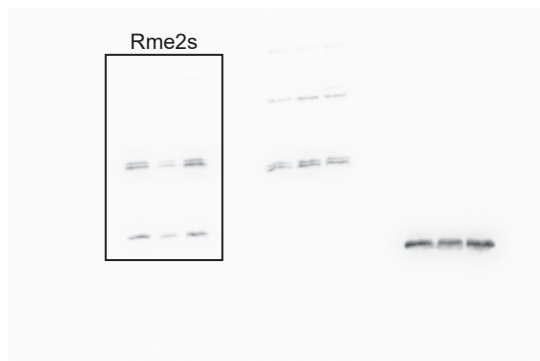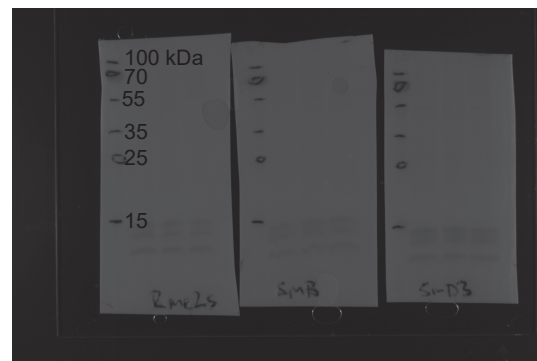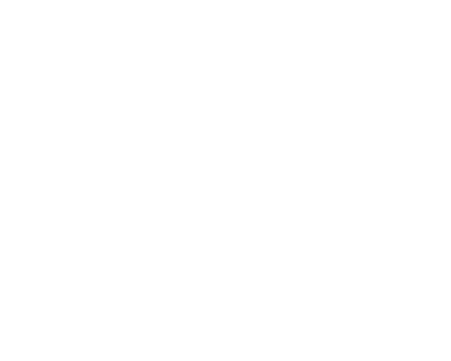

Supplement: Figure 1—figure supplement 1—source data 2. [file elife-72867-fig1-figsupp1-data2.zip › Figure1-figure_supplement_1-source data2/Figure 1-Figure Supplement 1-source data 2.pdf]

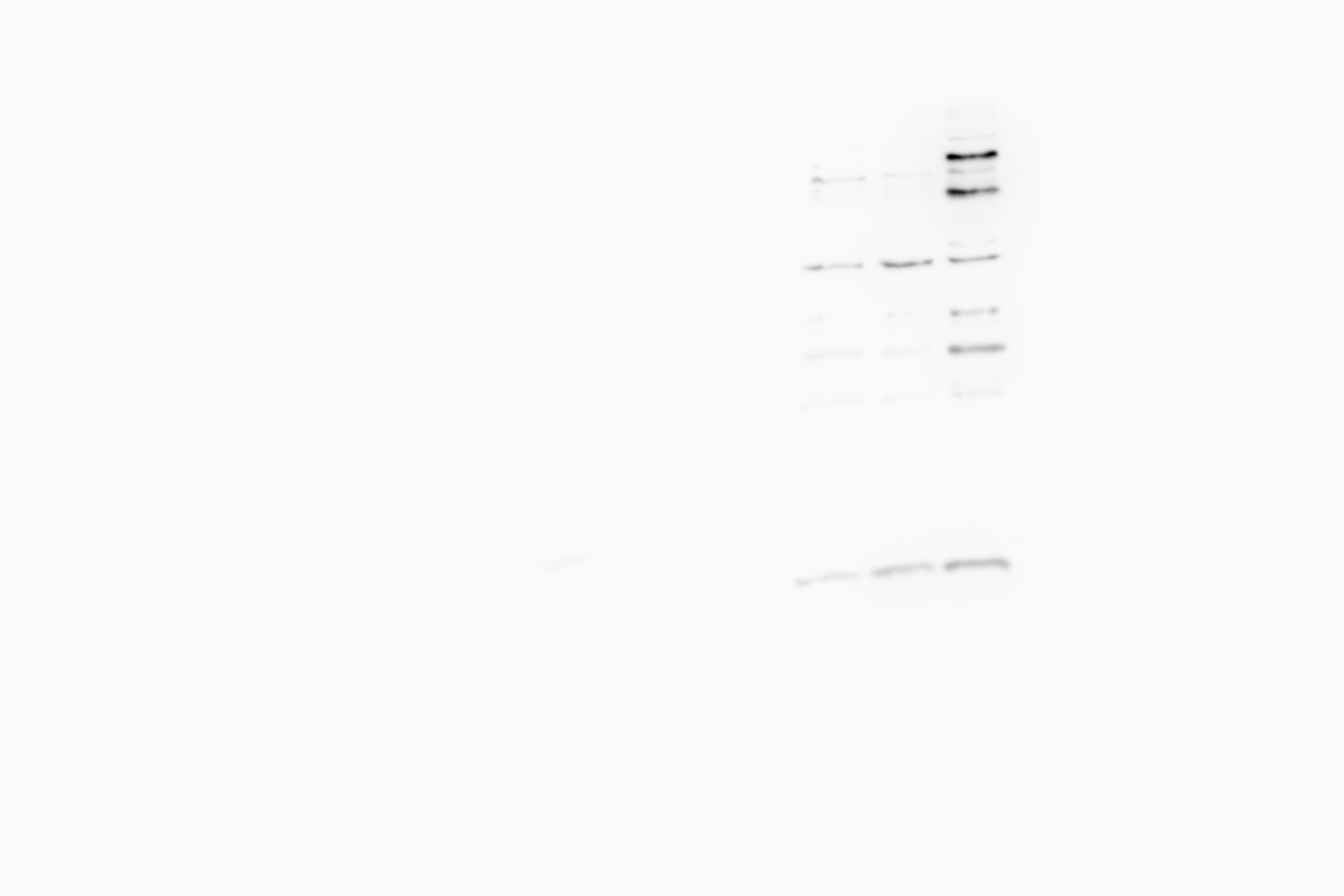

Supplement: Figure 1—figure supplement 1—source data 2. [file elife-72867-fig1-figsupp1-data2.zip › Figure1-figure_supplement_1-source data2/Rme2a_1.2000_Rme1_1.2000_10sec_Standard_9.tif]

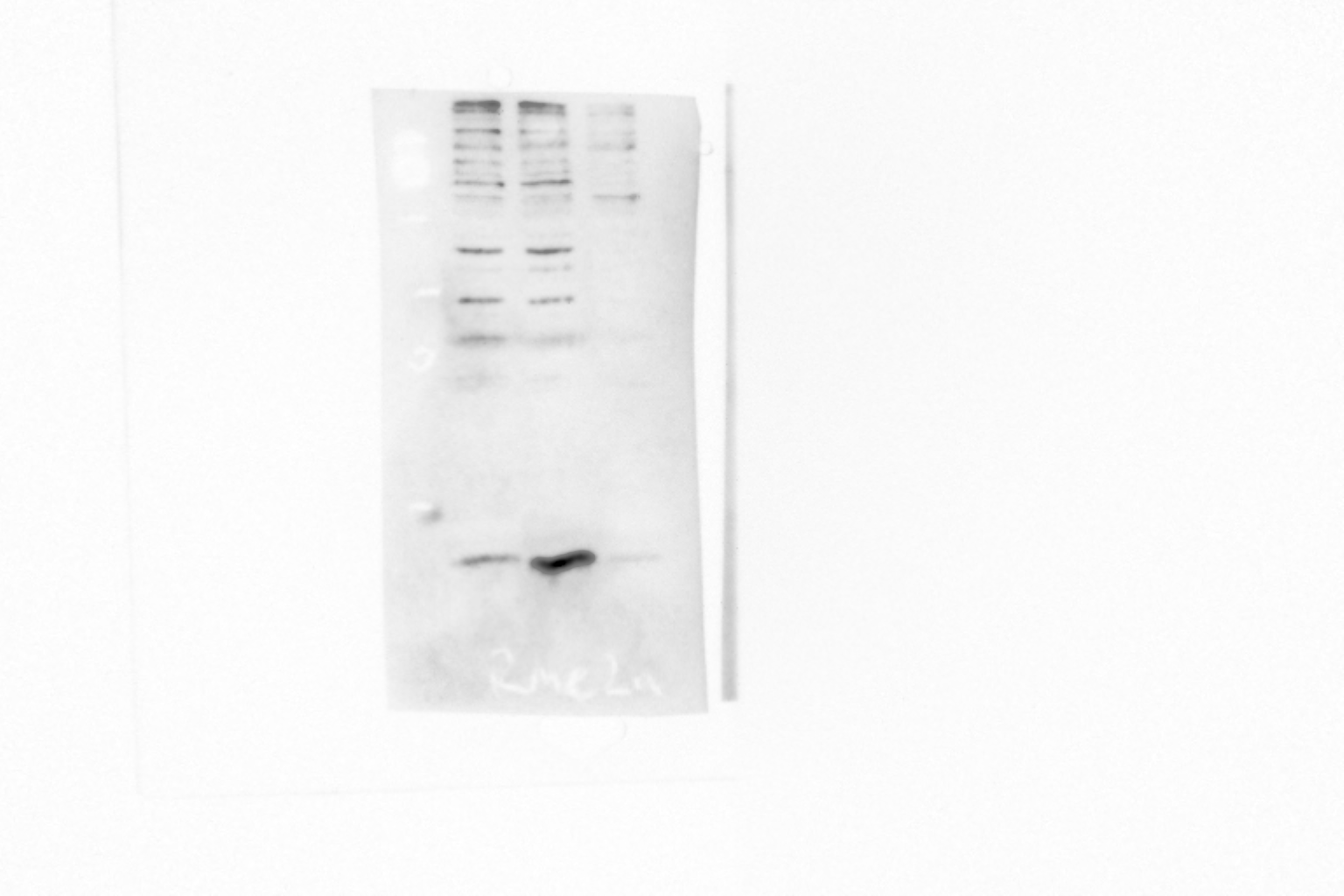

Supplement: Figure 1—figure supplement 1—source data 2. [file elife-72867-fig1-figsupp1-data2.zip › Figure1-figure_supplement_1-source data2/Rme2a_1.2000_Rme1_1.2000_60sec_High_15.tif]

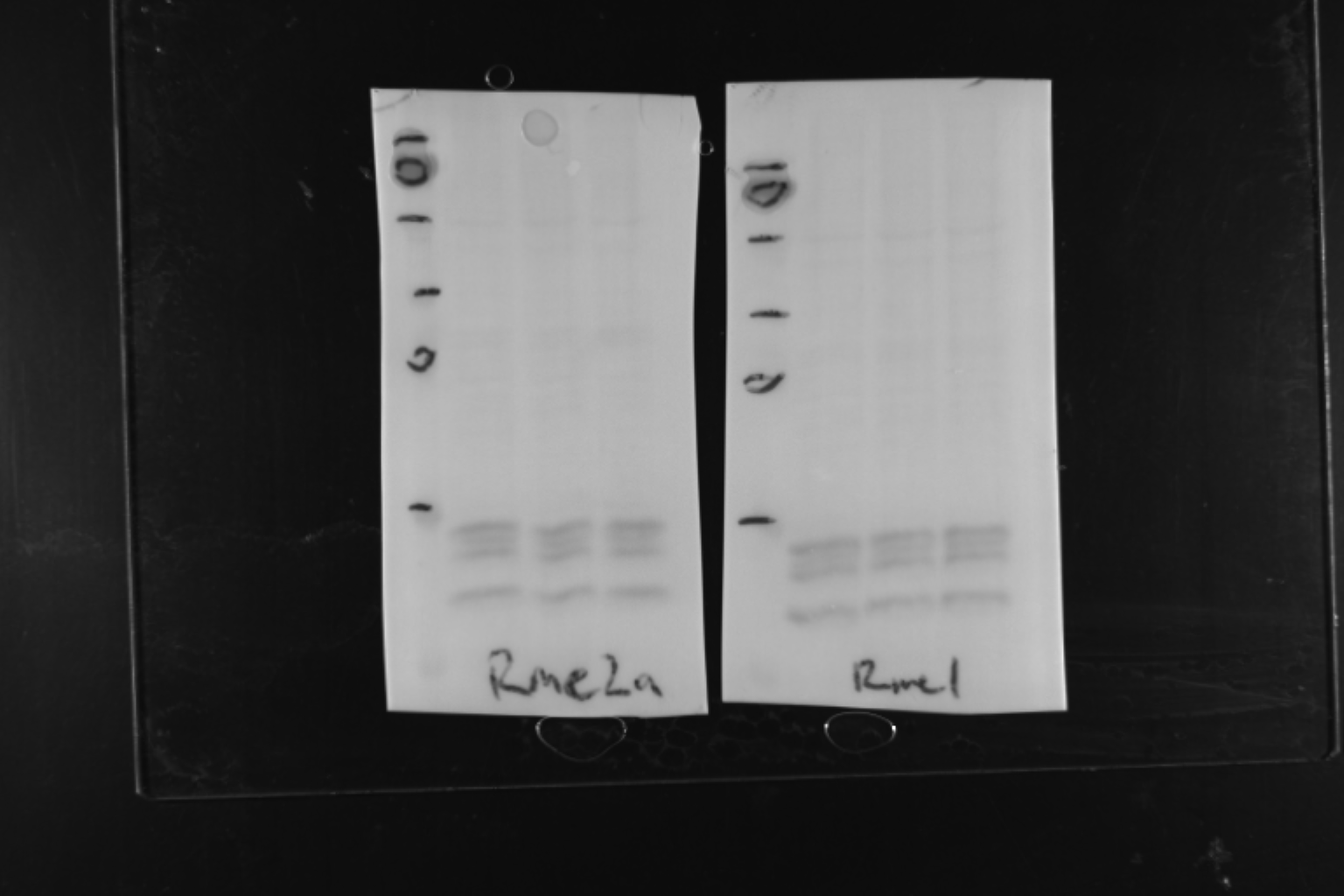

Supplement: Figure 1—figure supplement 1—source data 2. [file elife-72867-fig1-figsupp1-data2.zip › Figure1-figure_supplement_1-source data2/Rme2a_1.2000_Rme1_1.2000_60sec_High_ladder.tif]

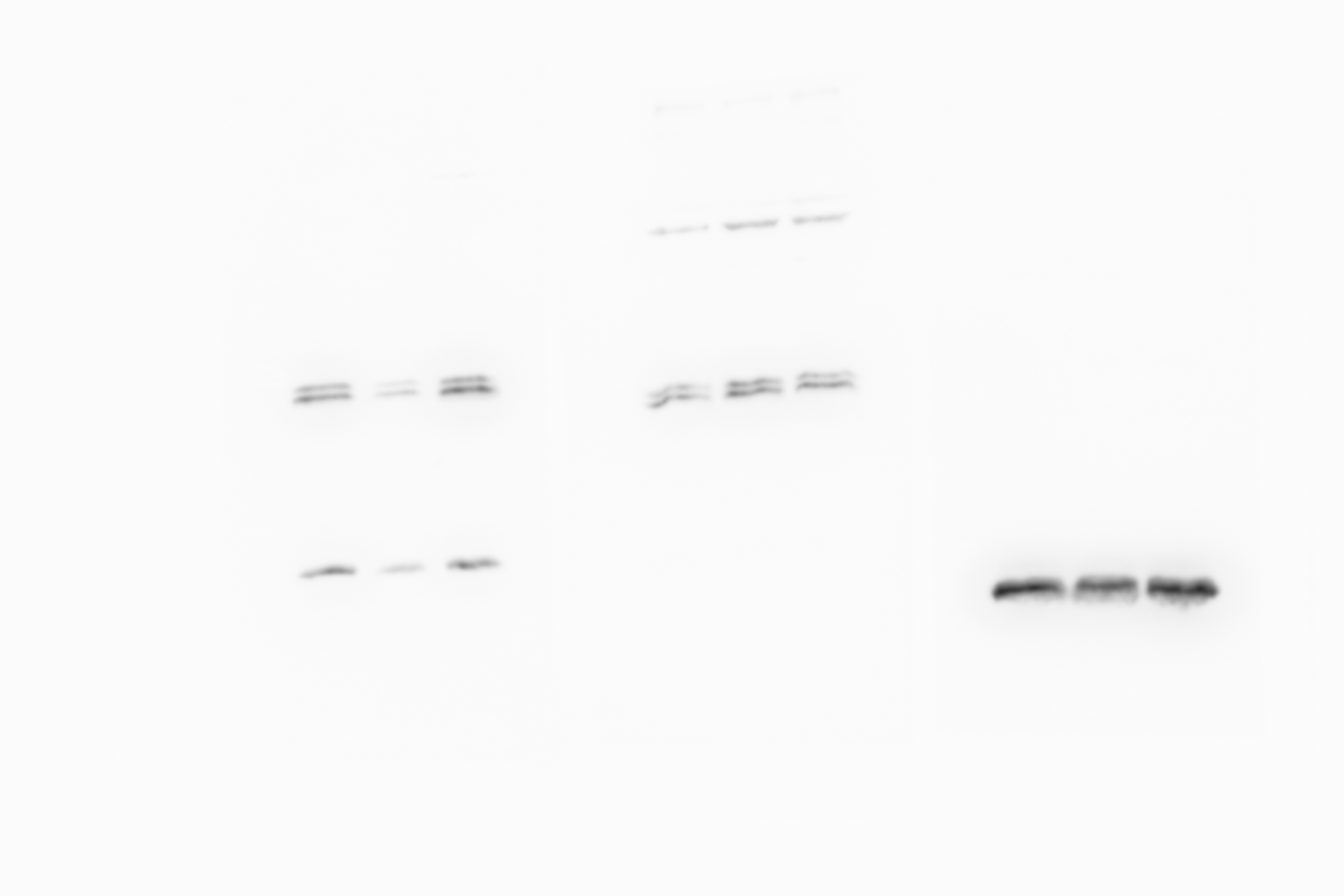

Supplement: Figure 1—figure supplement 1—source data 2. [file elife-72867-fig1-figsupp1-data2.zip › Figure1-figure_supplement_1-source data2/Rme2s_1.2000_SmB_1.2000_SmD3_1.2000_10sec_Standard_15.tif]

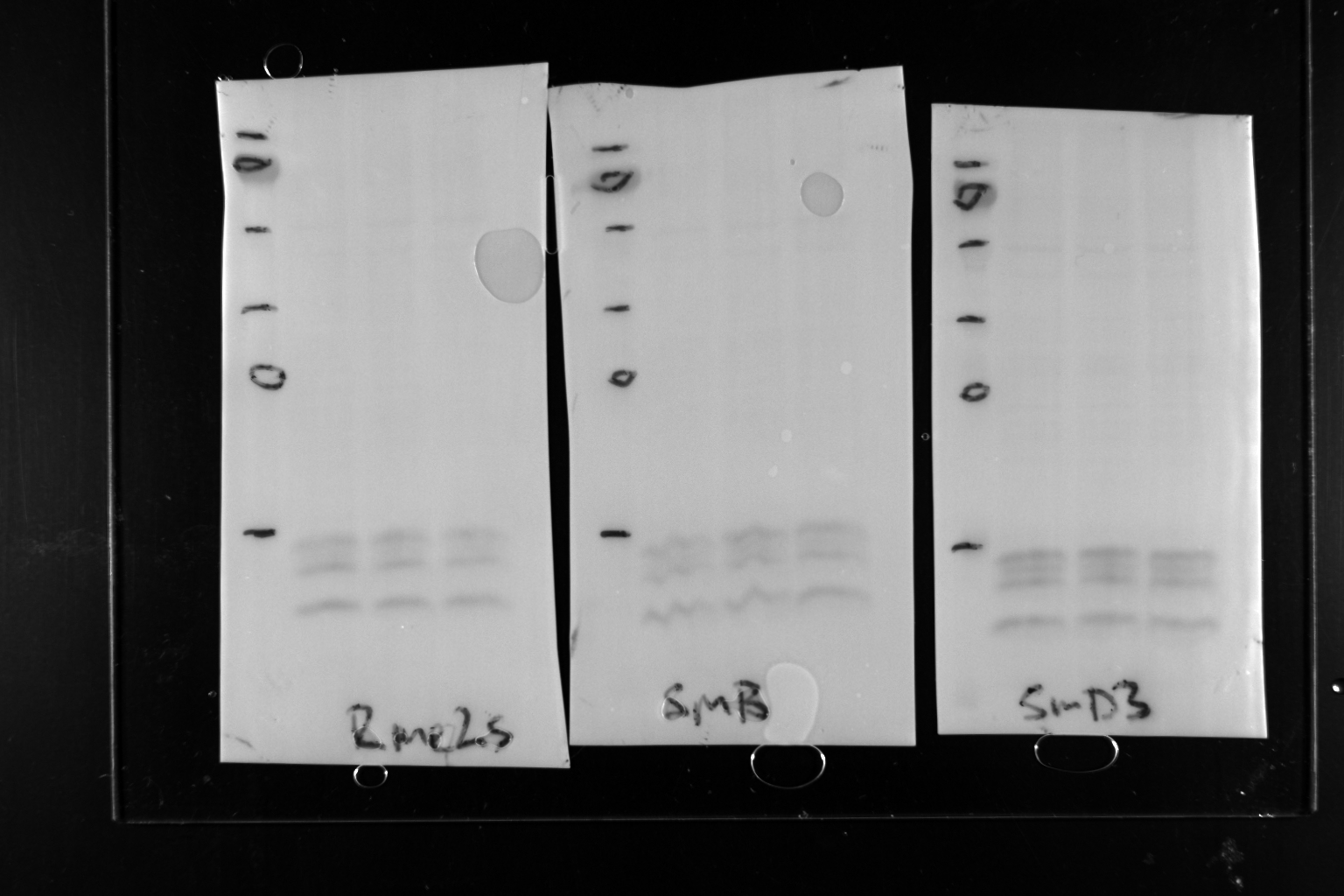

Supplement: Figure 1—figure supplement 1—source data 2. [file elife-72867-fig1-figsupp1-data2.zip › Figure1-figure_supplement_1-source data2/Rme2s_1.2000_SmB_1.2000_SmD3_1.2000_10sec_Standard_ladder.tif]

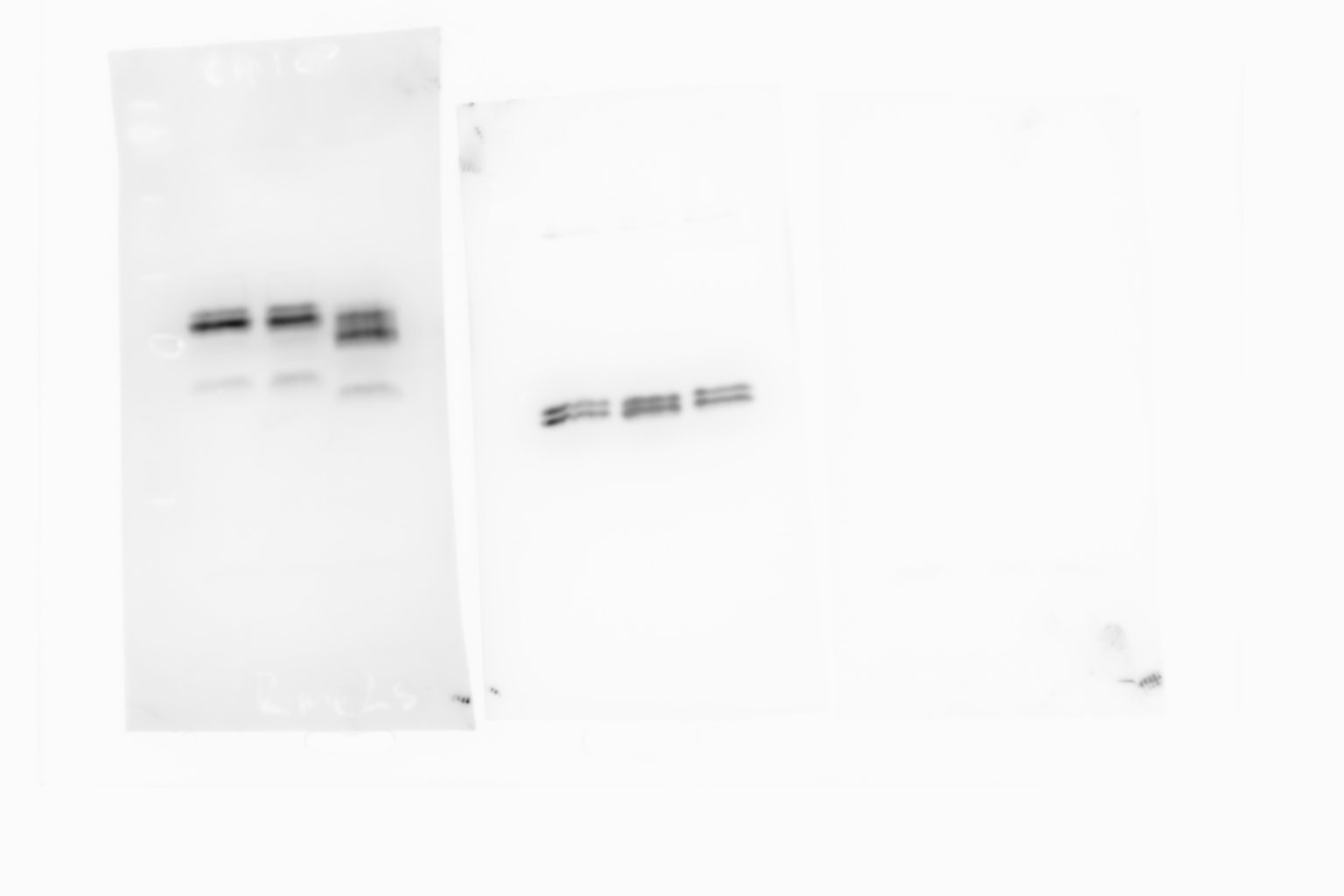

Supplement: Figure 4—source data 1. [file elife-72867-fig4-data1.zip › Figure4-source data1/CHTOP_1.2000_SNRPB_1.2000_SNRPD3_1.50000_30sec_Std_4.tif]

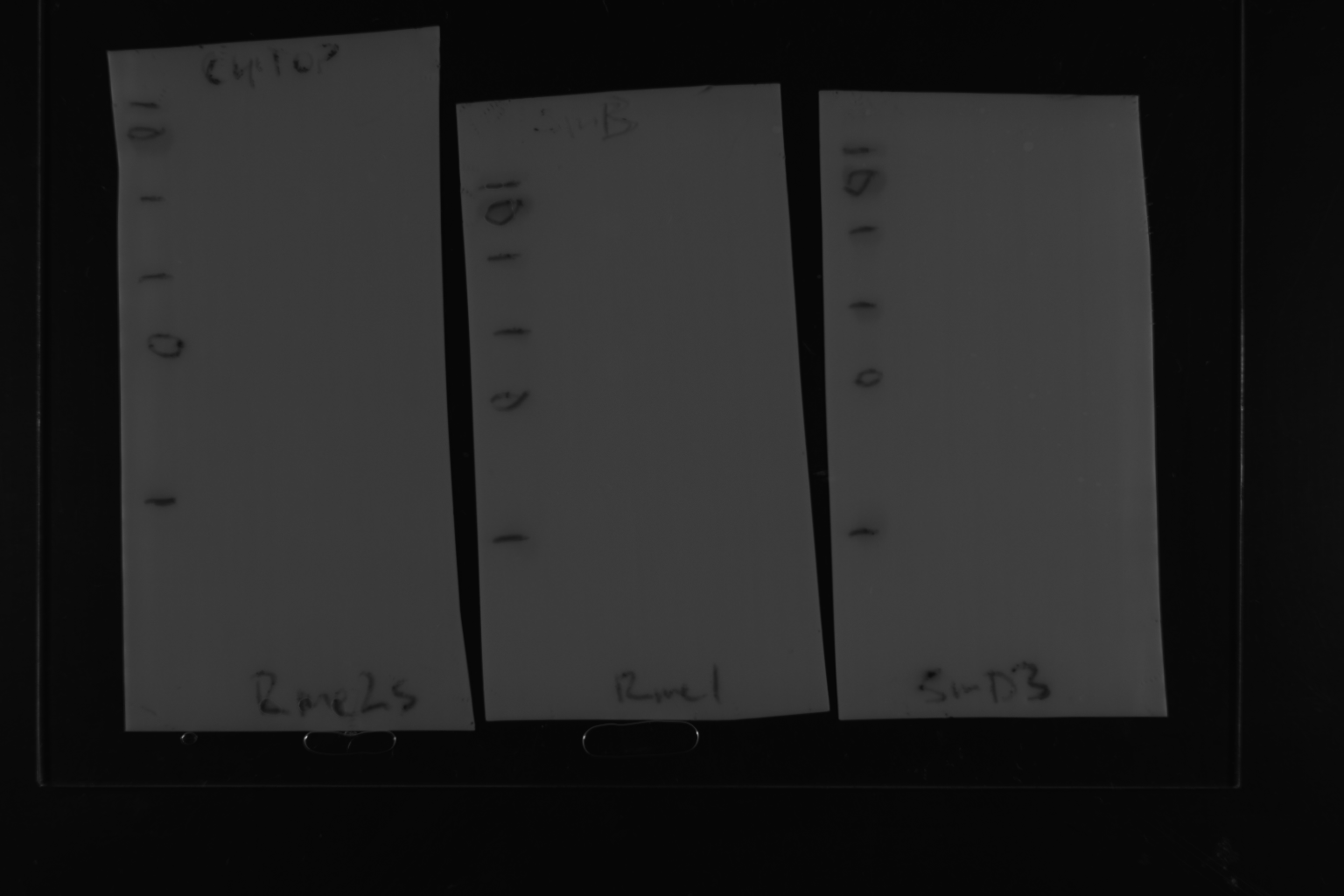

Supplement: Figure 4—source data 1. [file elife-72867-fig4-data1.zip › Figure4-source data1/CHTOP_1.2000_SNRPB_1.2000_SNRPD3_1.50000_30sec_Std_4_ladder.tif]

f

Chemiluminescence

Digital

Direct Blue 71 Membrane Stain

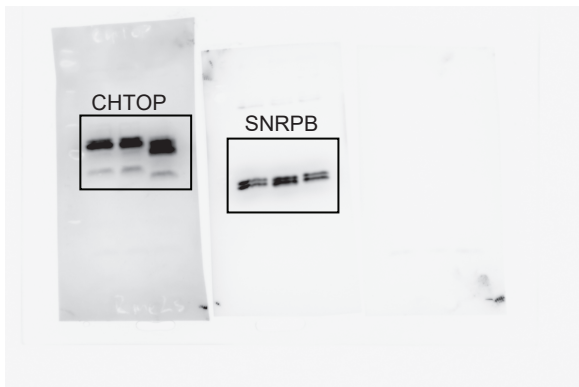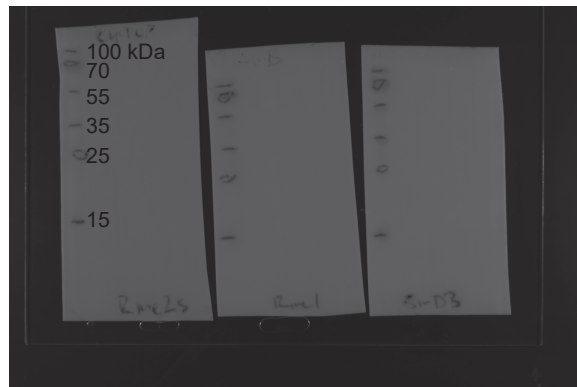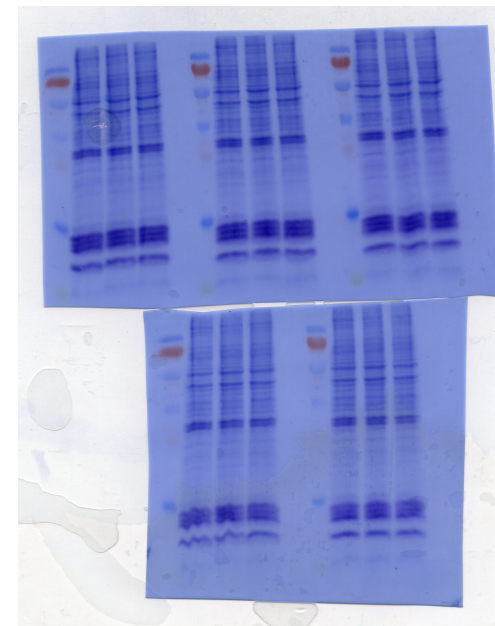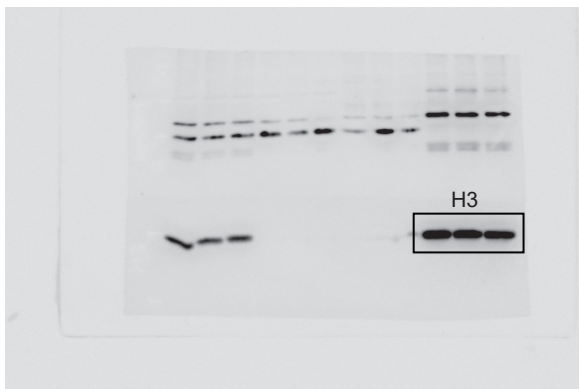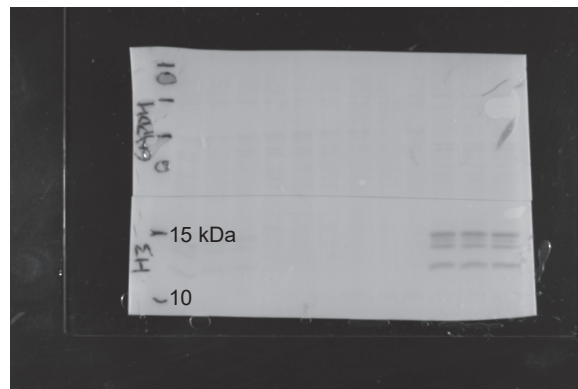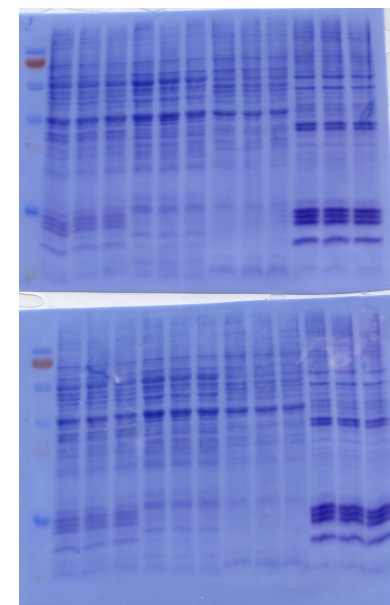

Supplement: Figure 4—source data 1. [file elife-72867-fig4-data1.zip › Figure4-source data1/Figure 4-source data 1.pdf]

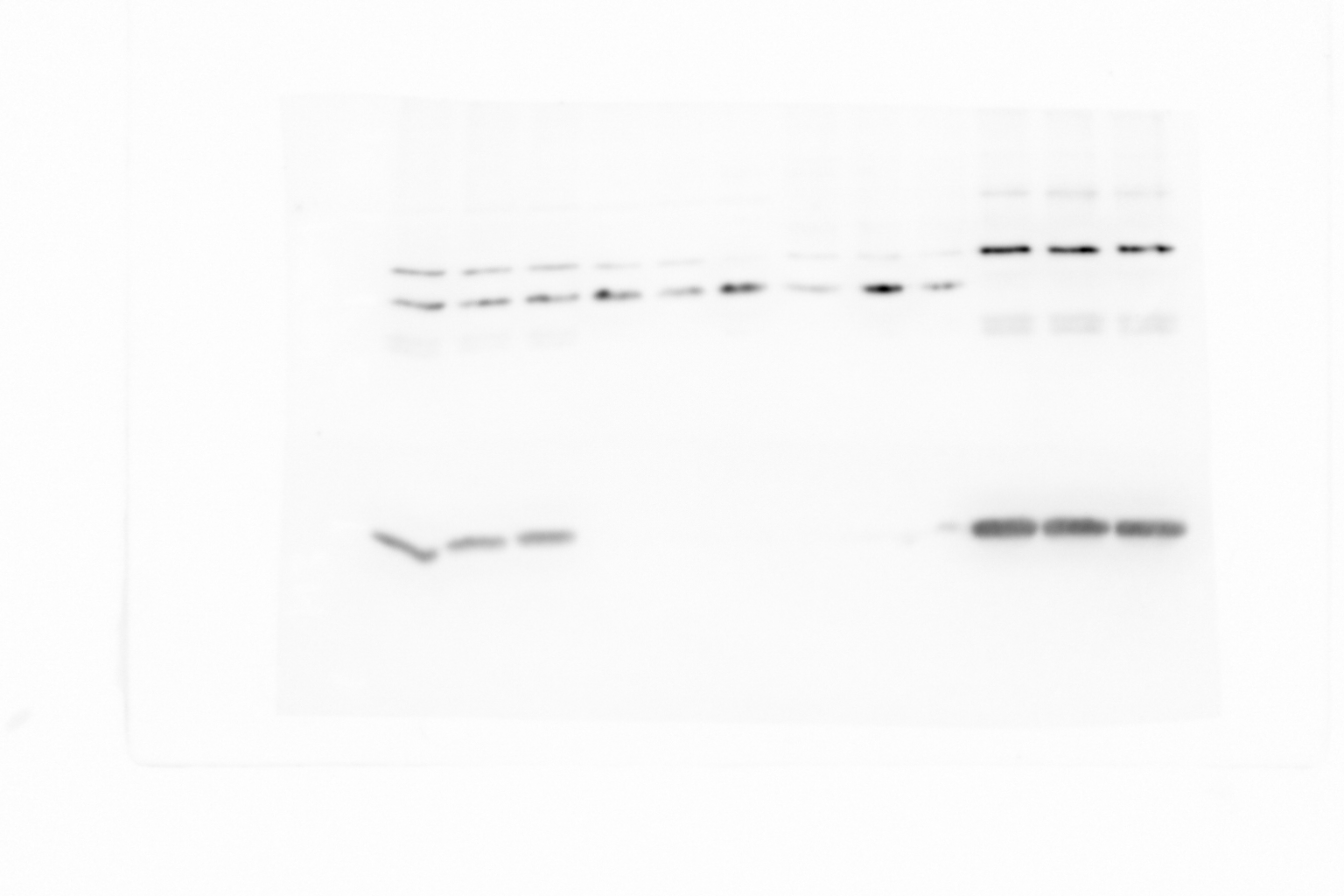

Supplement: Figure 4—source data 1. [file elife-72867-fig4-data1.zip › Figure4-source data1/PRMTi_2d_Fractionation_GAPDH_1.10000_H3_1.100000_30sec_Standard_13.tif]

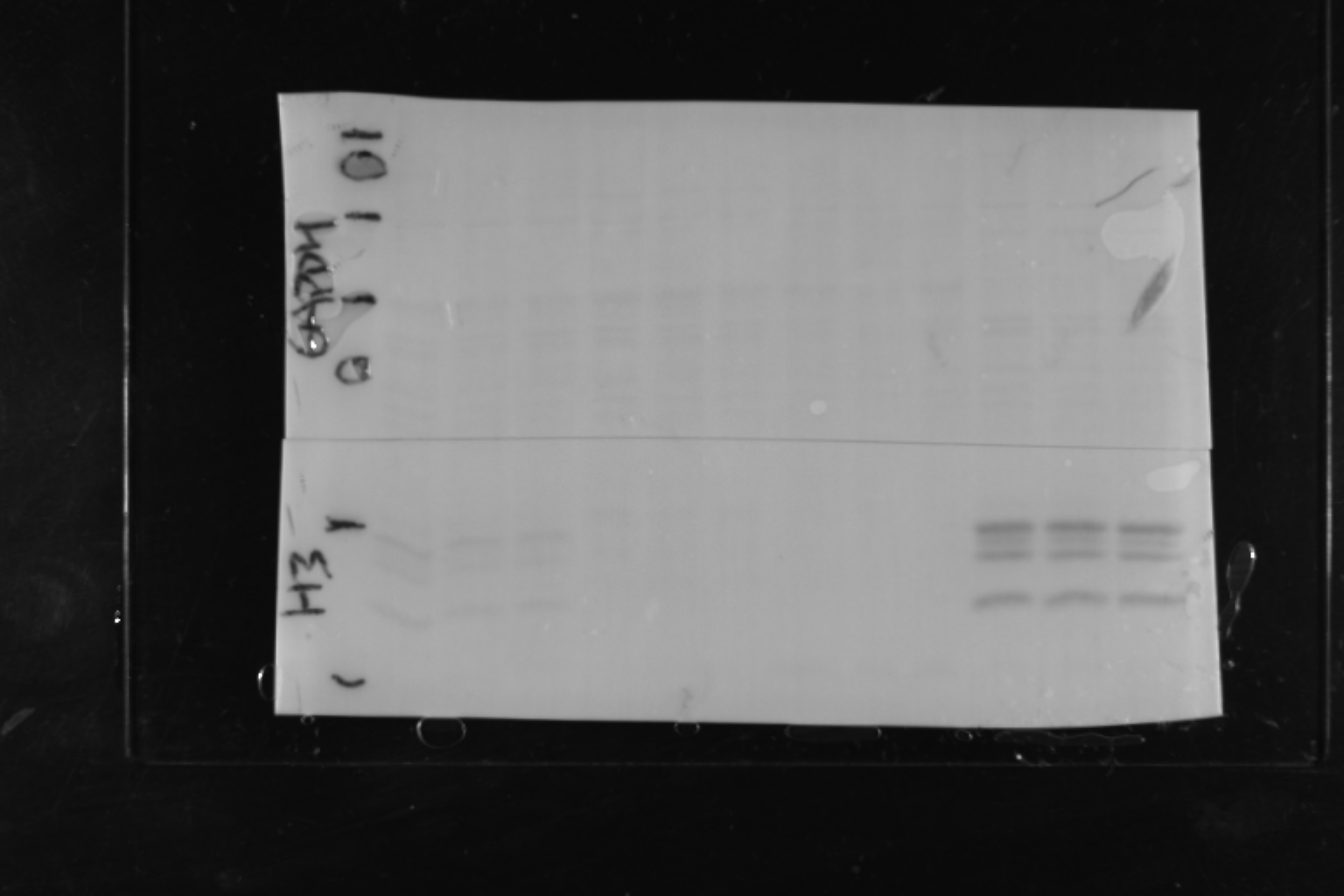

Supplement: Figure 4—source data 1. [file elife-72867-fig4-data1.zip › Figure4-source data1/PRMTi_2d_Fractionation_GAPDH_1.10000_H3_1.100000_30sec_Standard_ladder.tif]

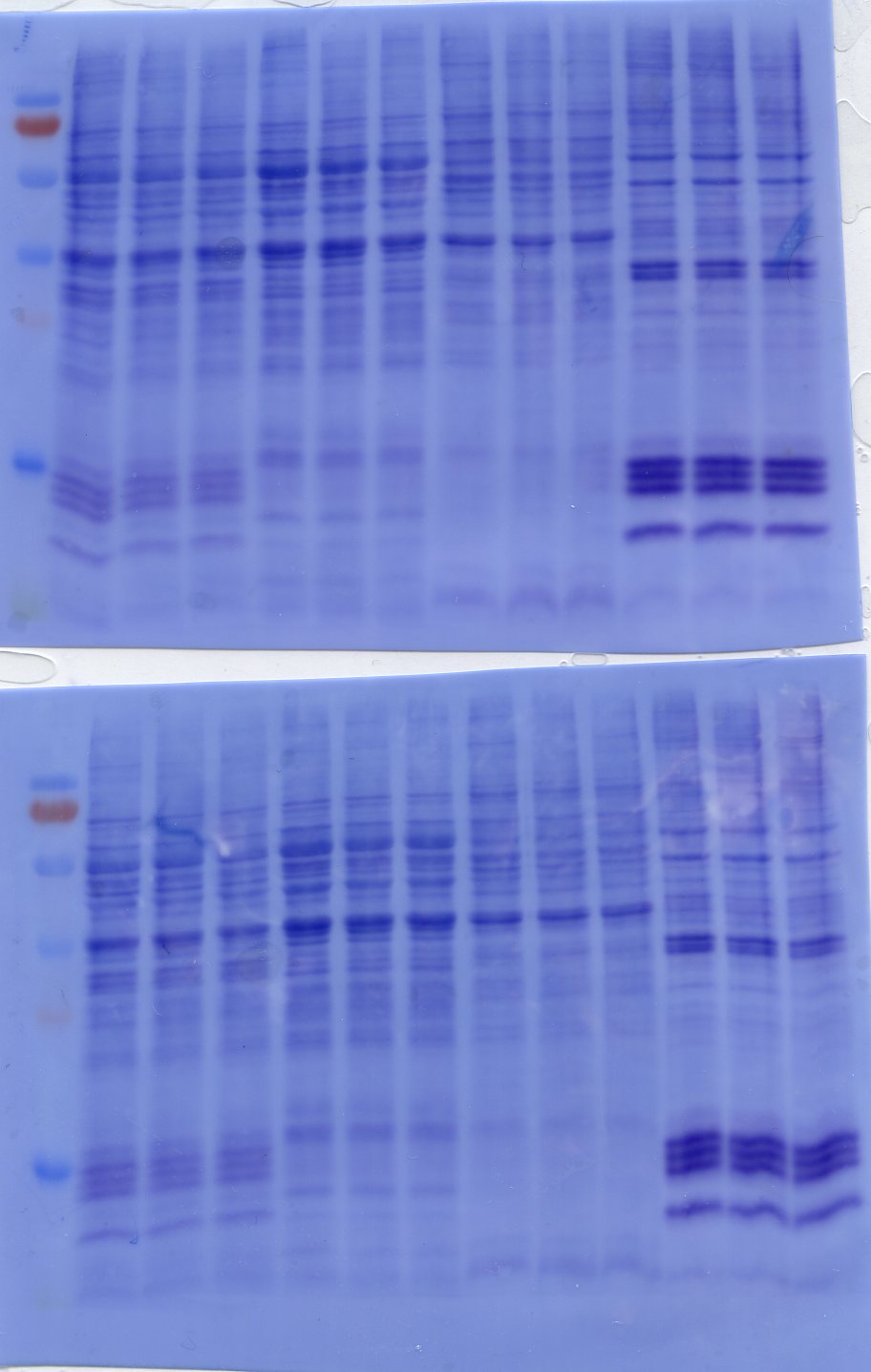

Supplement: Figure 4—source data 1. [file elife-72867-fig4-data1.zip › Figure4-source data1/PRMTi_48h_Fractionation_DB71.jpg]

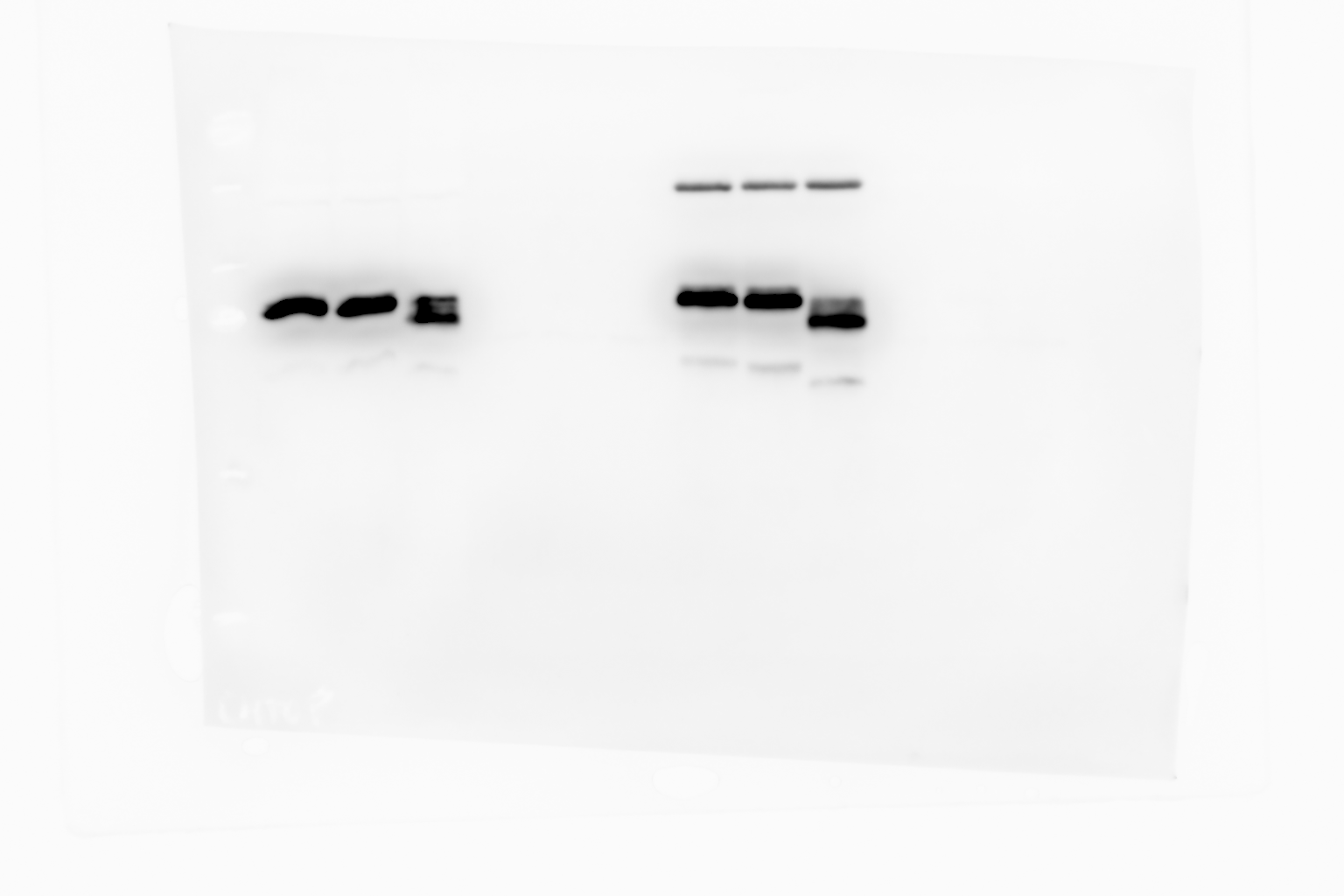

Supplement: Figure 4—source data 2. [file elife-72867-fig4-data2.zip › Figure4-source data2/A549_48h_PRMTi_CHTOP_1.2000_30sec_Standard_10.tif]

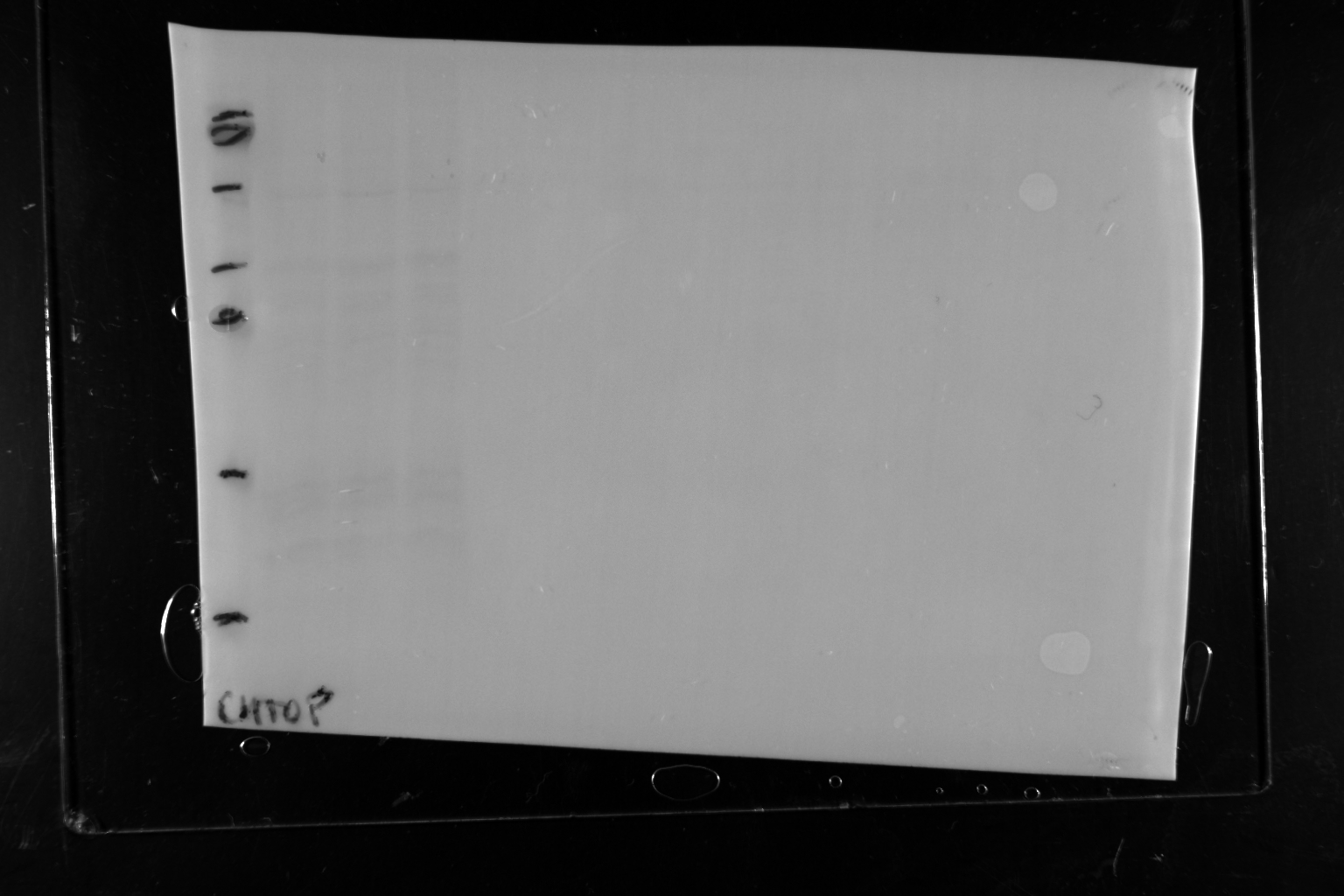

Supplement: Figure 4—source data 2. [file elife-72867-fig4-data2.zip › Figure4-source data2/A549_48h_PRMTi_CHTOP_1.2000_30sec_Standard_ladder.tif]

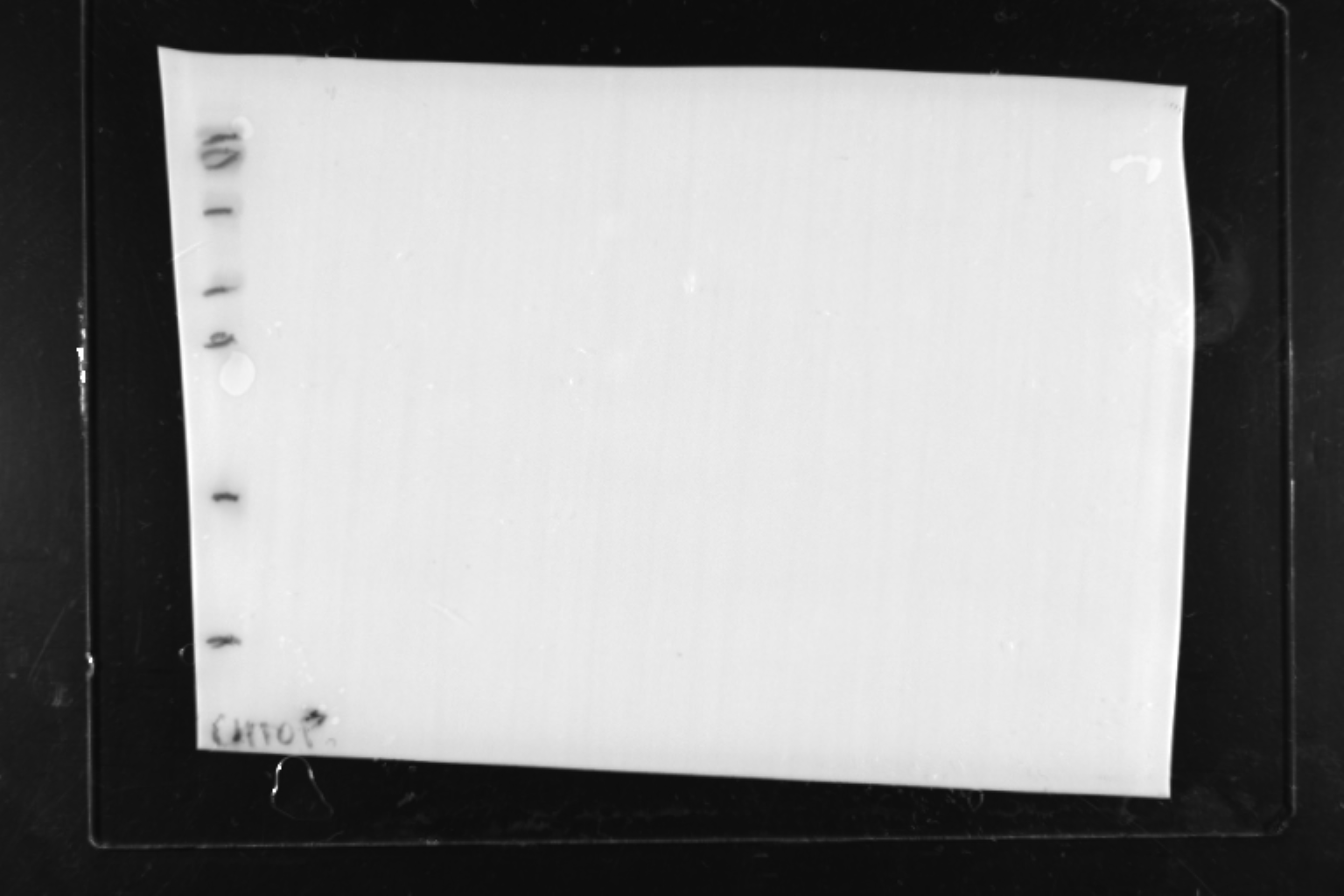

Supplement: Figure 4—source data 2. [file elife-72867-fig4-data2.zip › Figure4-source data2/A549_48h_PRMTi_Rme2a_1.2000_120sec_High_lader.tif]

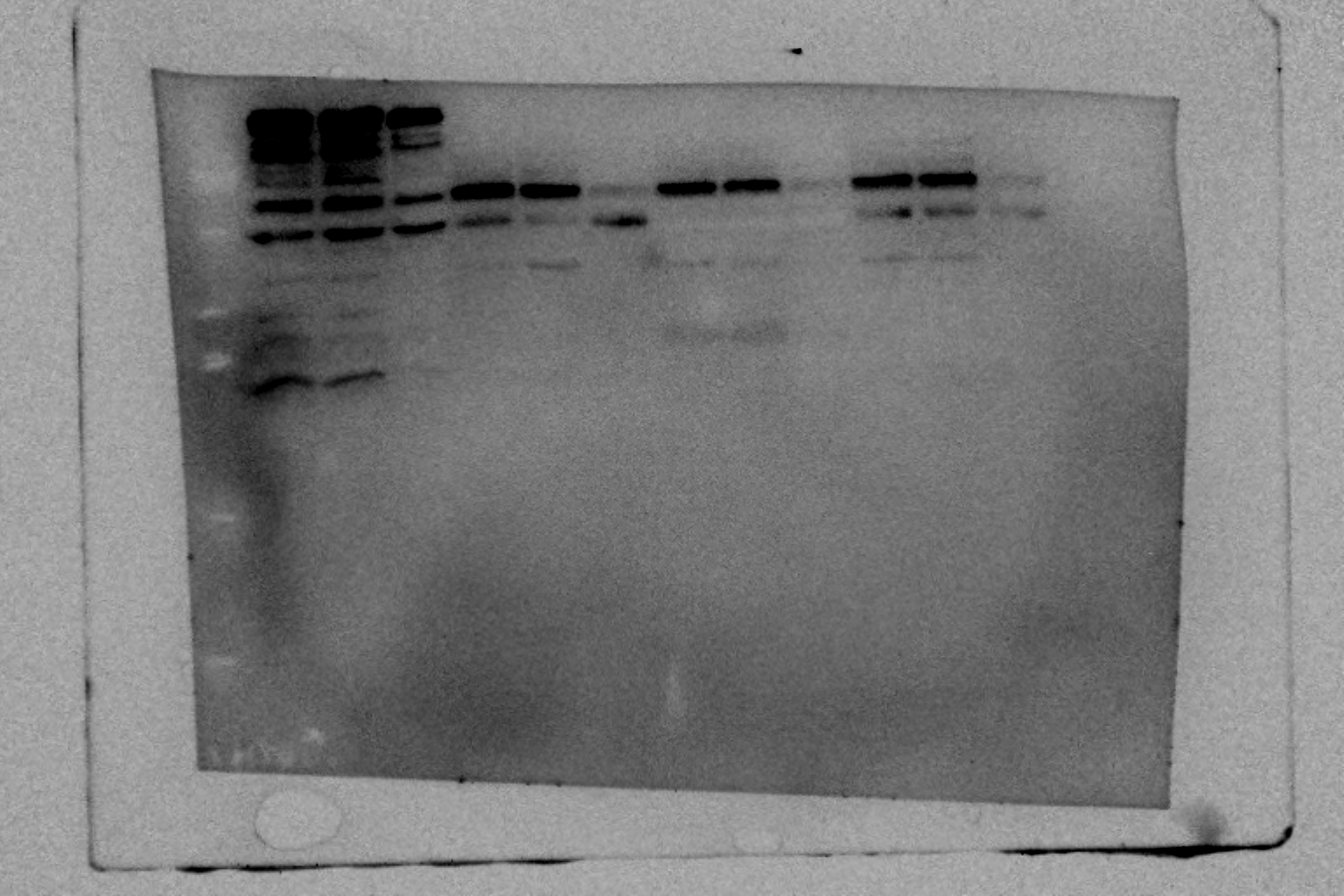

Supplement: Figure 4—source data 2. [file elife-72867-fig4-data2.zip › Figure4-source data2/A549_48h_PRMTi_Rme2a_1.2000_60sec_High_15.tif]

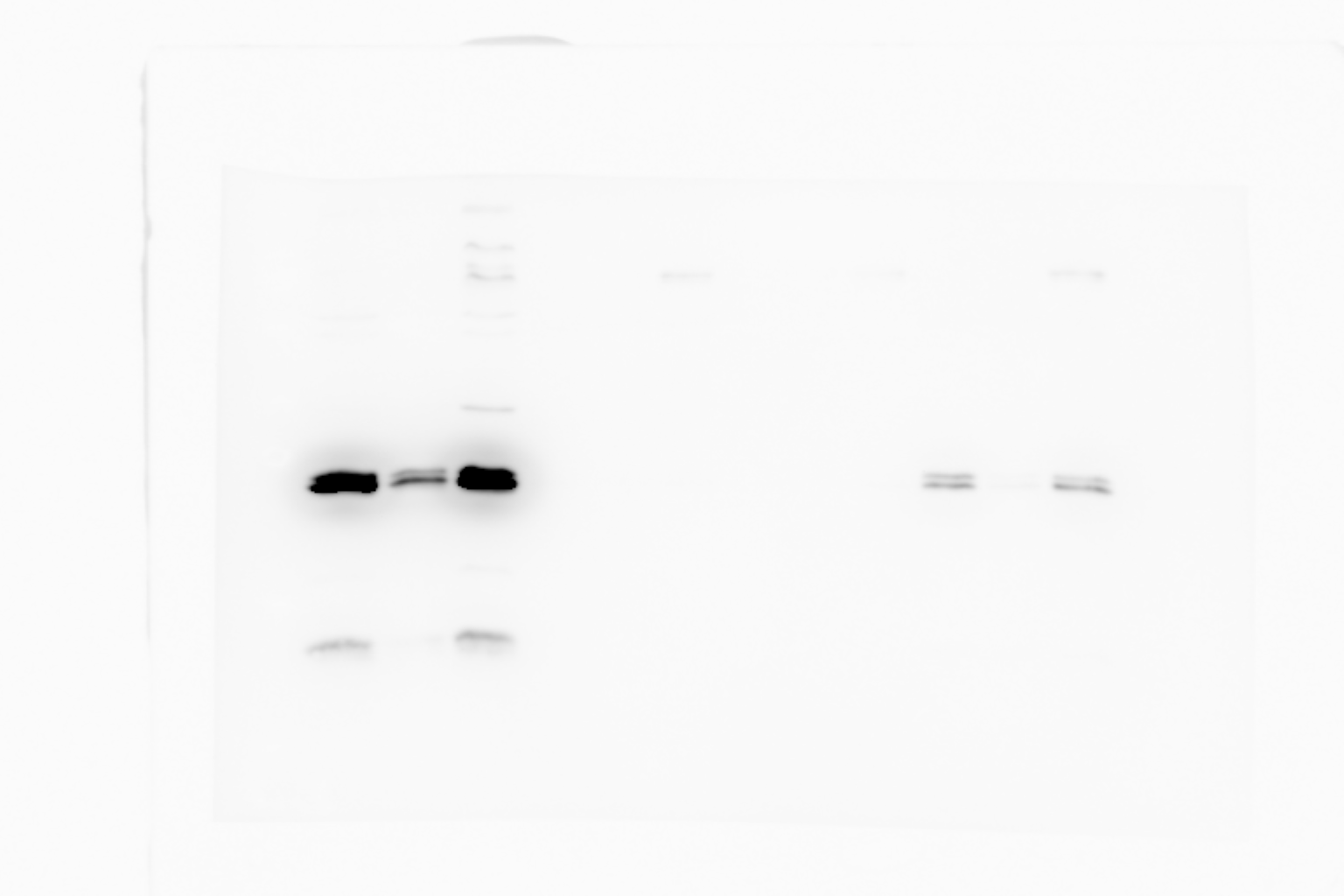

Supplement: Figure 4—source data 2. [file elife-72867-fig4-data2.zip › Figure4-source data2/A549_48h_PRMTi_Rme2s_1.2000_30sec_High_10.tif]

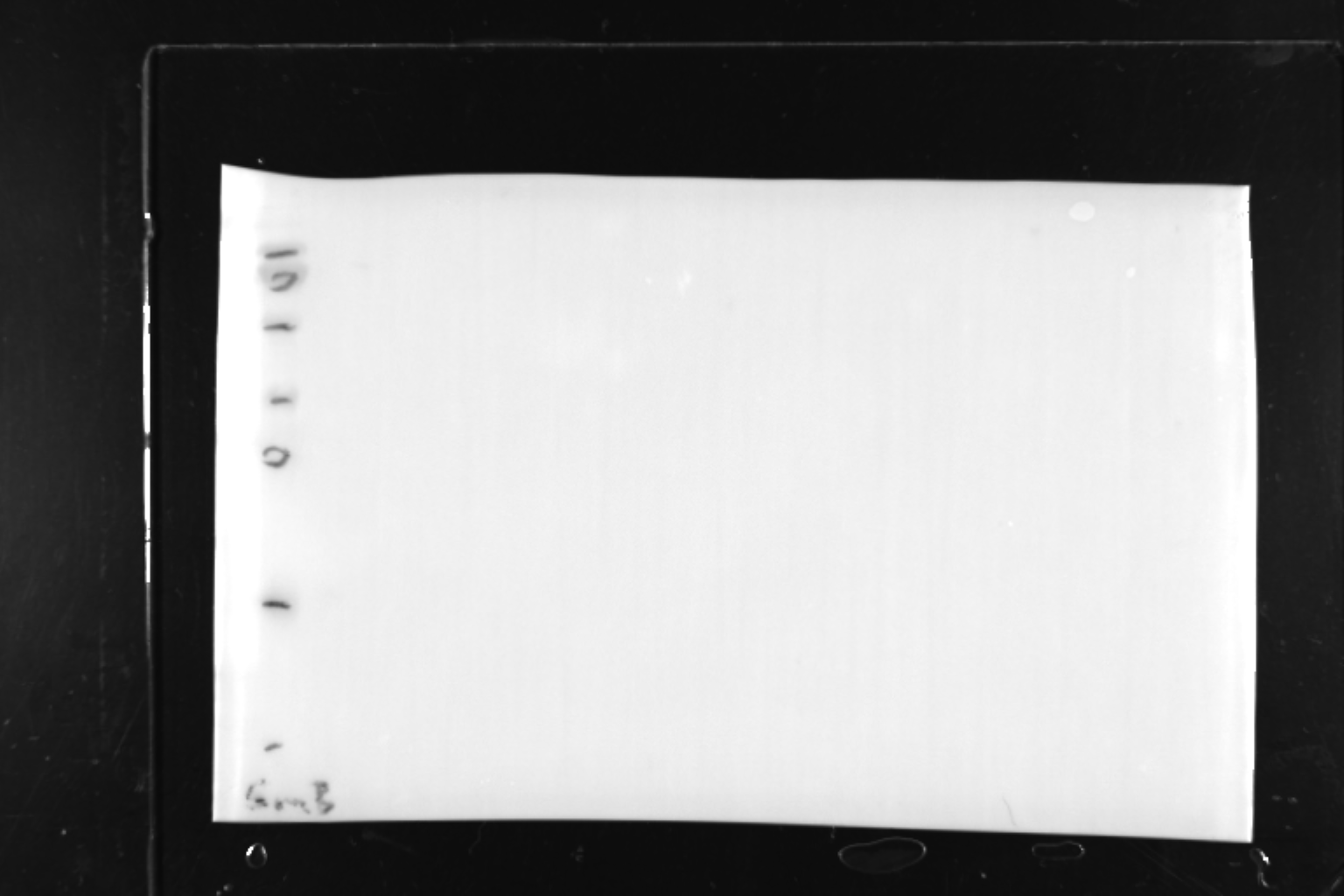

Supplement: Figure 4—source data 2. [file elife-72867-fig4-data2.zip › Figure4-source data2/A549_48h_PRMTi_Rme2s_1.2000_30sec_High_ladder.tif]

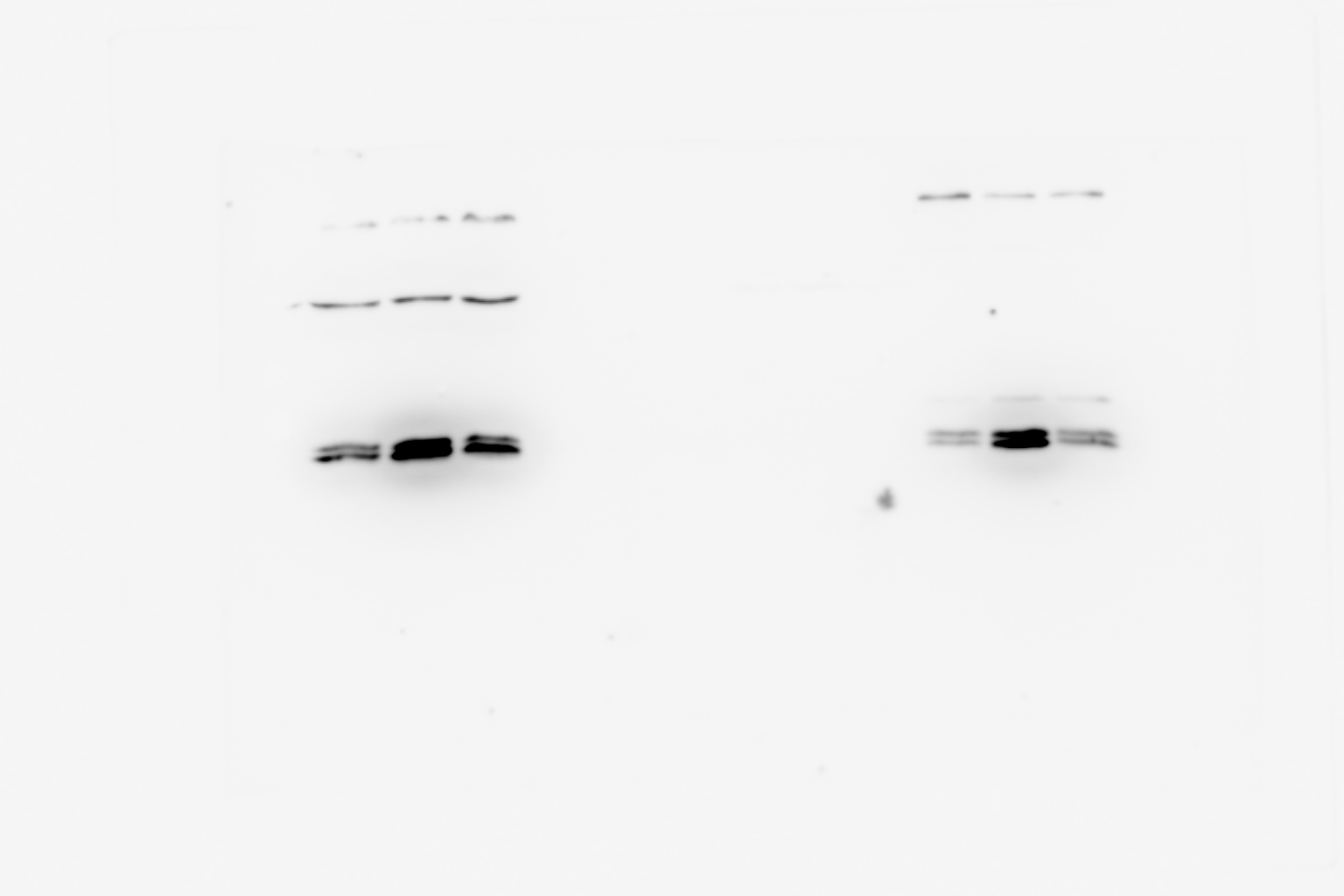

Supplement: Figure 4—source data 2. [file elife-72867-fig4-data2.zip › Figure4-source data2/A549_48h_PRMTi_SNRPB_1.2000_30sec_High_1.tif]

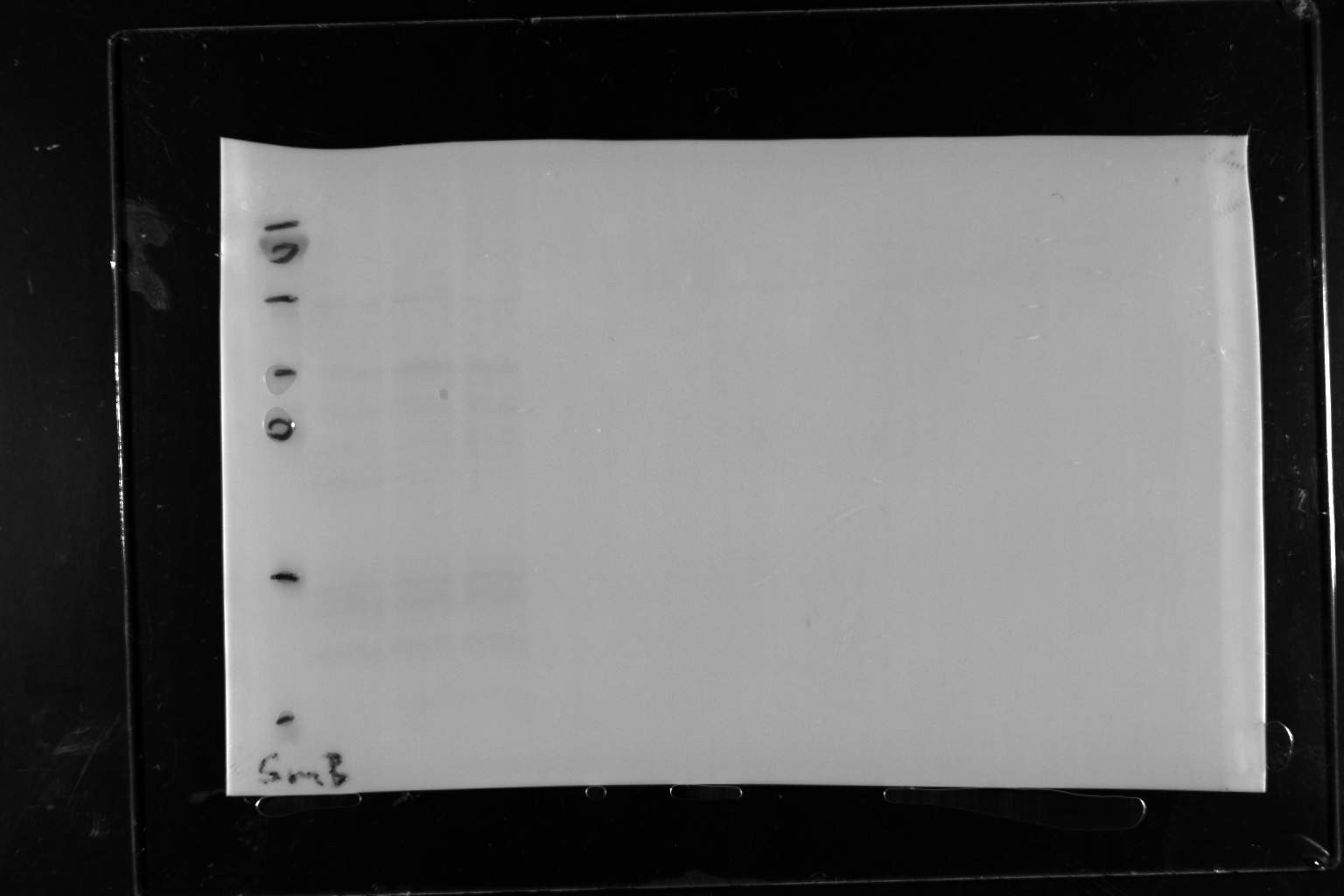

Supplement: Figure 4—source data 2. [file elife-72867-fig4-data2.zip › Figure4-source data2/A549_48h_PRMTi_SNRPB_1.2000_30sec_Standard_ladder.tif]

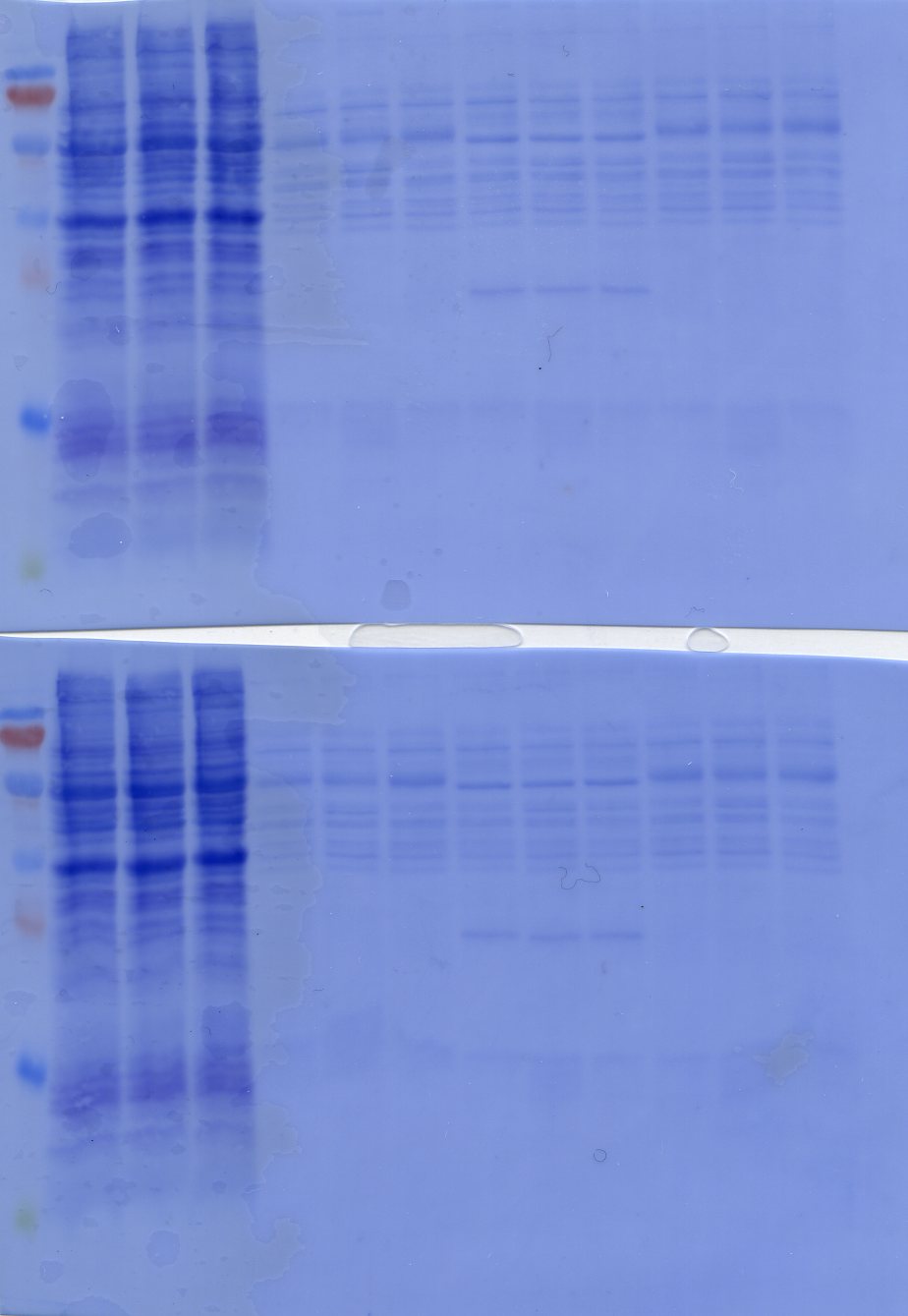

Supplement: Figure 4—source data 2. [file elife-72867-fig4-data2.zip › Figure4-source data2/CHTOP_SNRPB_IP_DB71.jpg]

g

Chemiluminescence

Digital

Direct Blue 71 Membrane Stain

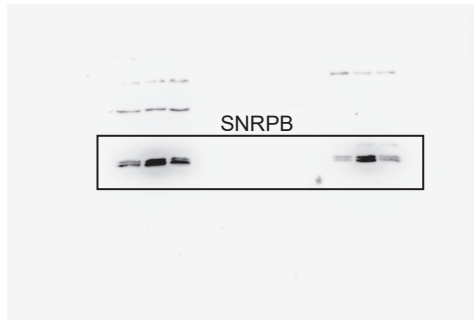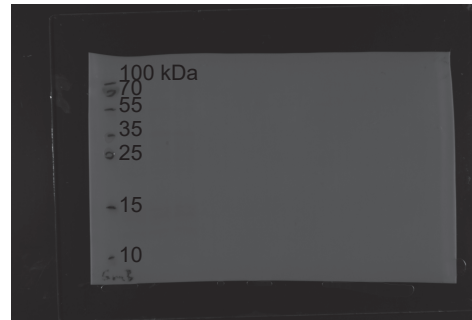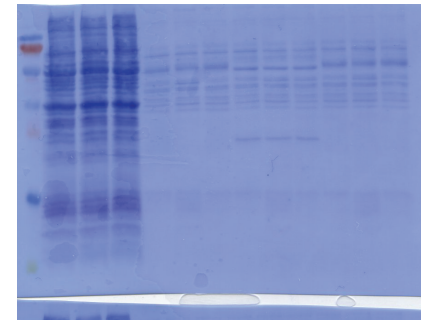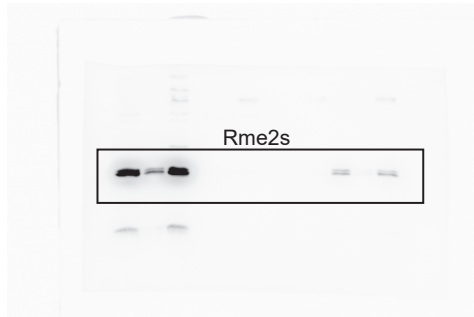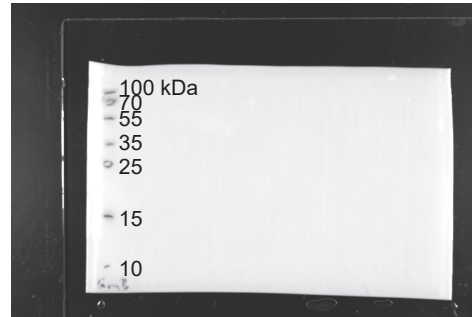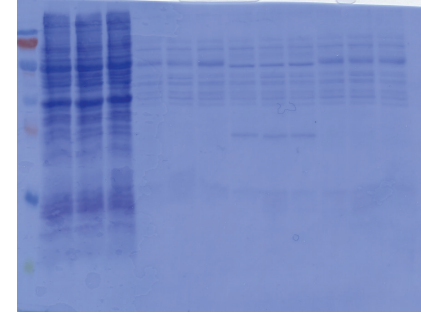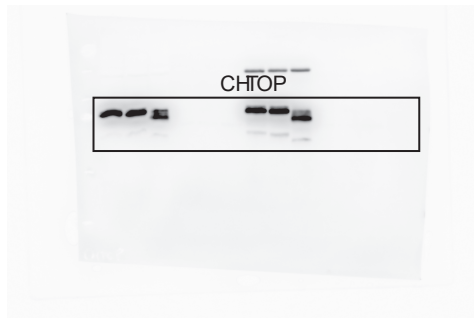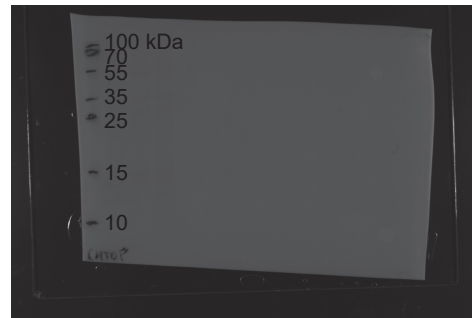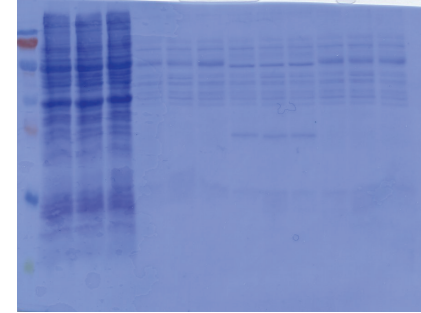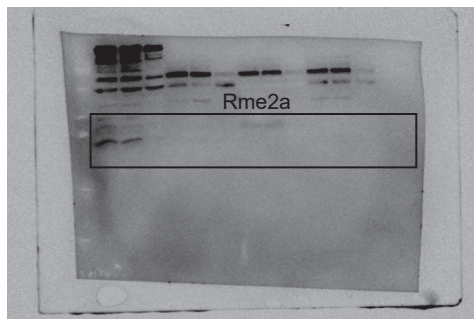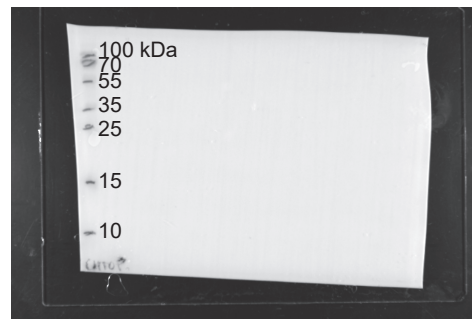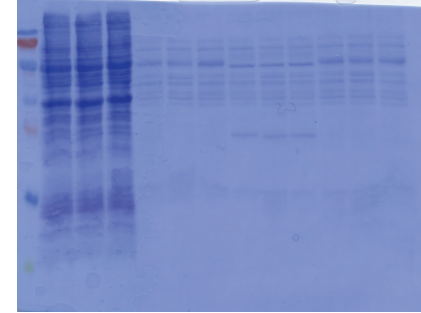

Supplement: Figure 4—source data 2. [file elife-72867-fig4-data2.zip › Figure4-source data2/Figure 4-source data 2.pdf]

c

Chemiluminescence

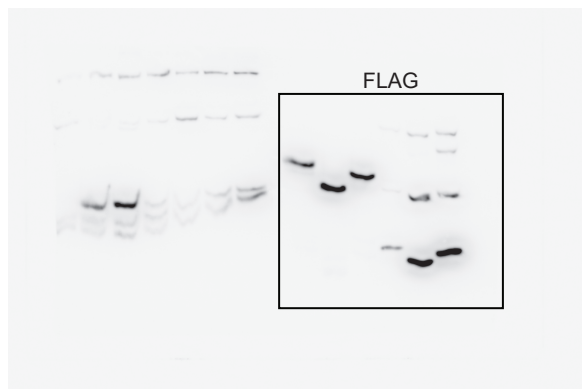

Digital

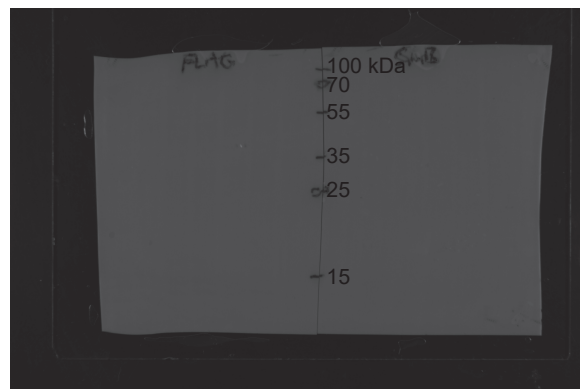

Direct Blue 71 Membrane Stain

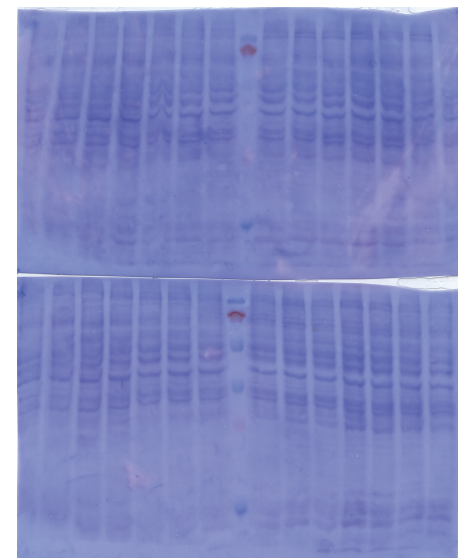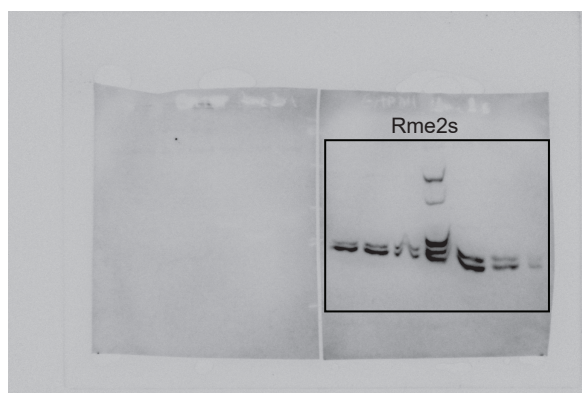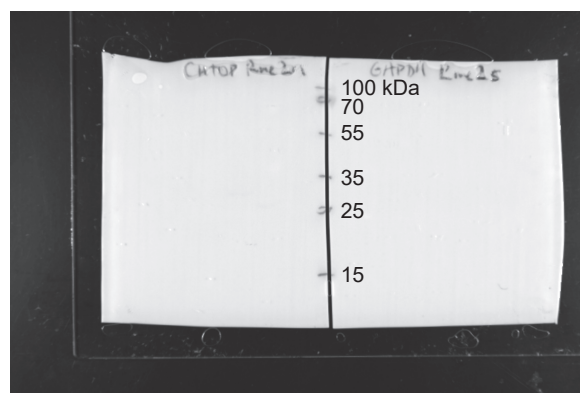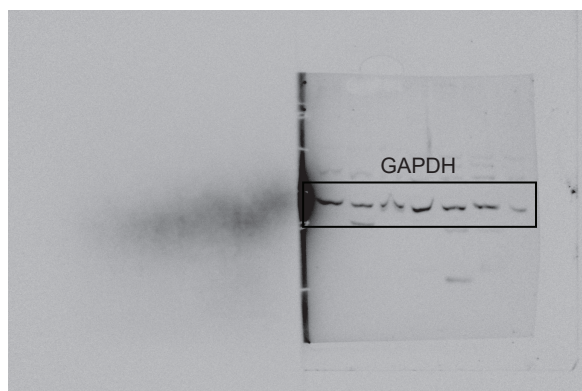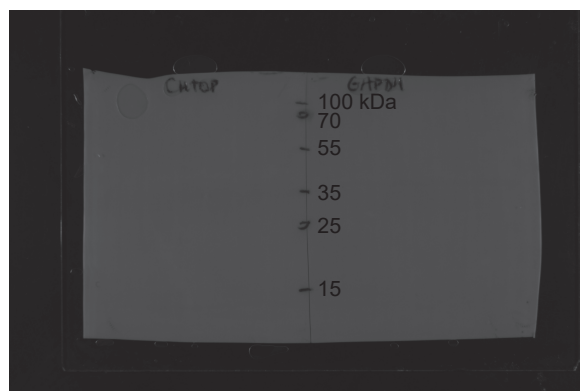

Supplement: Figure 5—figure supplement 1—source data 1. [file elife-72867-fig5-figsupp1-data1.zip › Figure5-figure_supplement_1-source data1/Figure 5-Figure Supplement 1-source data 1.pdf]

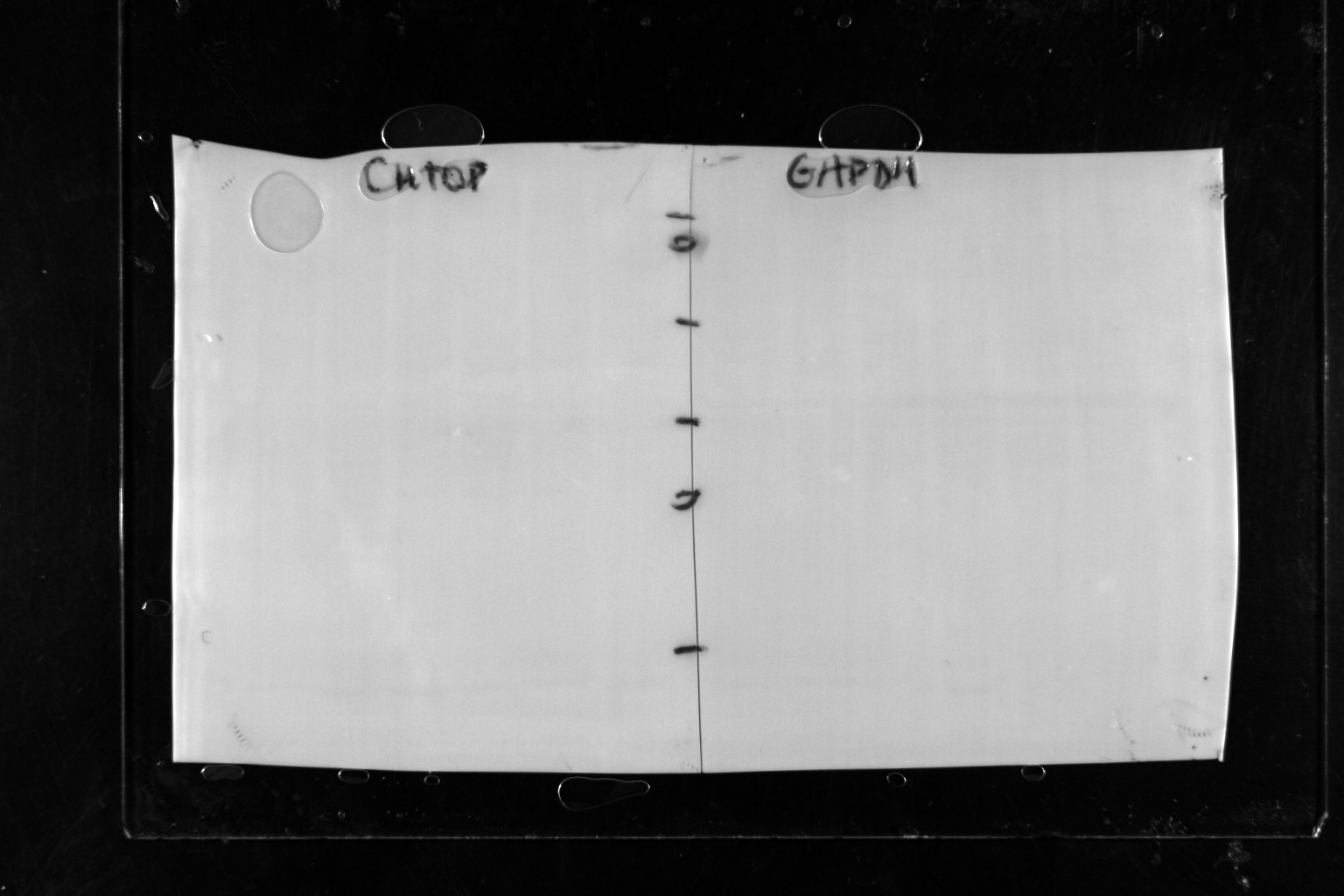

Supplement: Figure 5—figure supplement 1—source data 1. [file elife-72867-fig5-figsupp1-data1.zip › Figure5-figure_supplement_1-source data1/SmExp_CHTOPexp_CHTOP_1.2000_GAPDH_1.10000_30sec_Standard_ladder.tif]

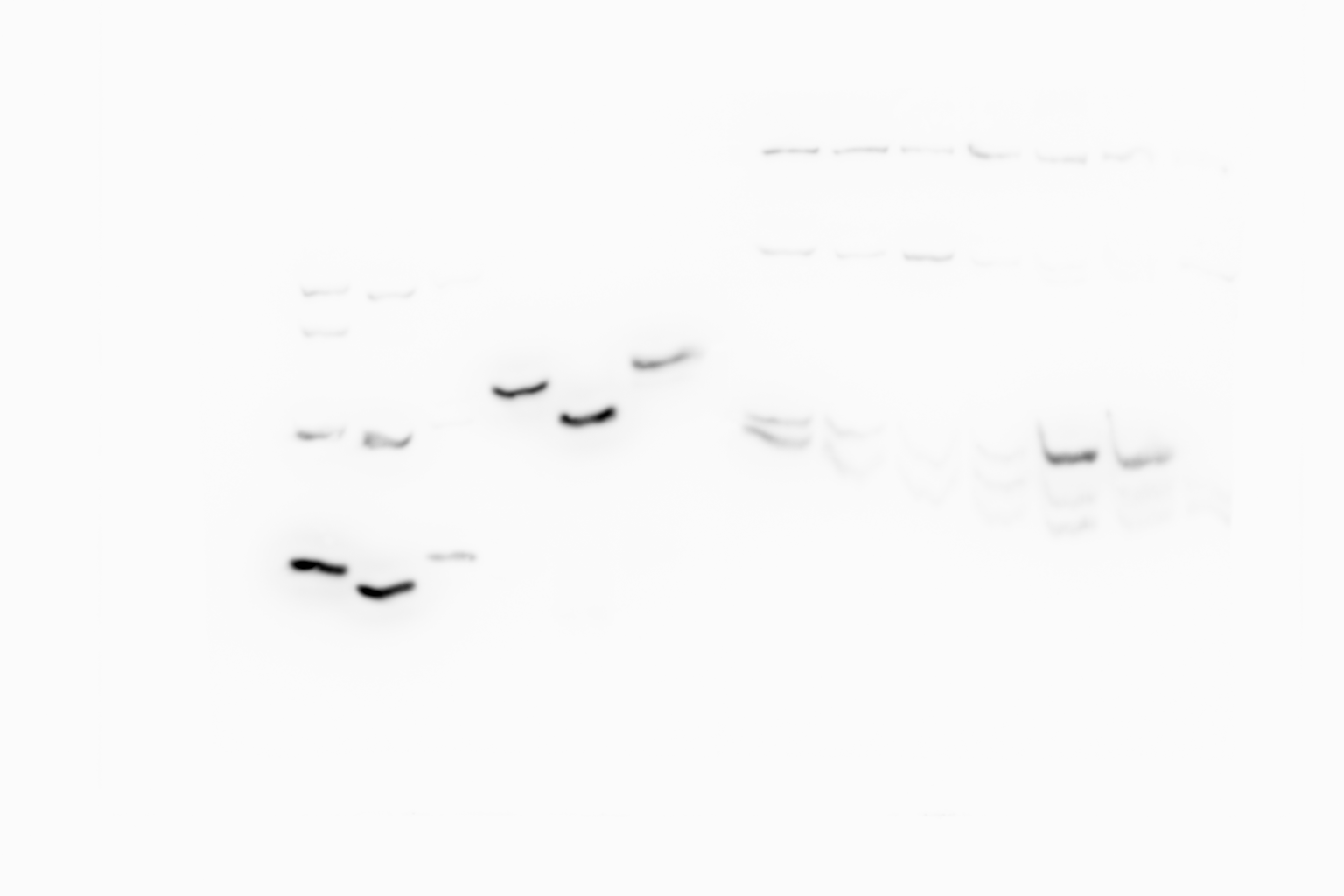

Supplement: Figure 5—figure supplement 1—source data 1. [file elife-72867-fig5-figsupp1-data1.zip › Figure5-figure_supplement_1-source data1/SmExp_CHTOPexp_FLAG_1.10000_SmB_1.2000_30sec_Standard_4.tif]

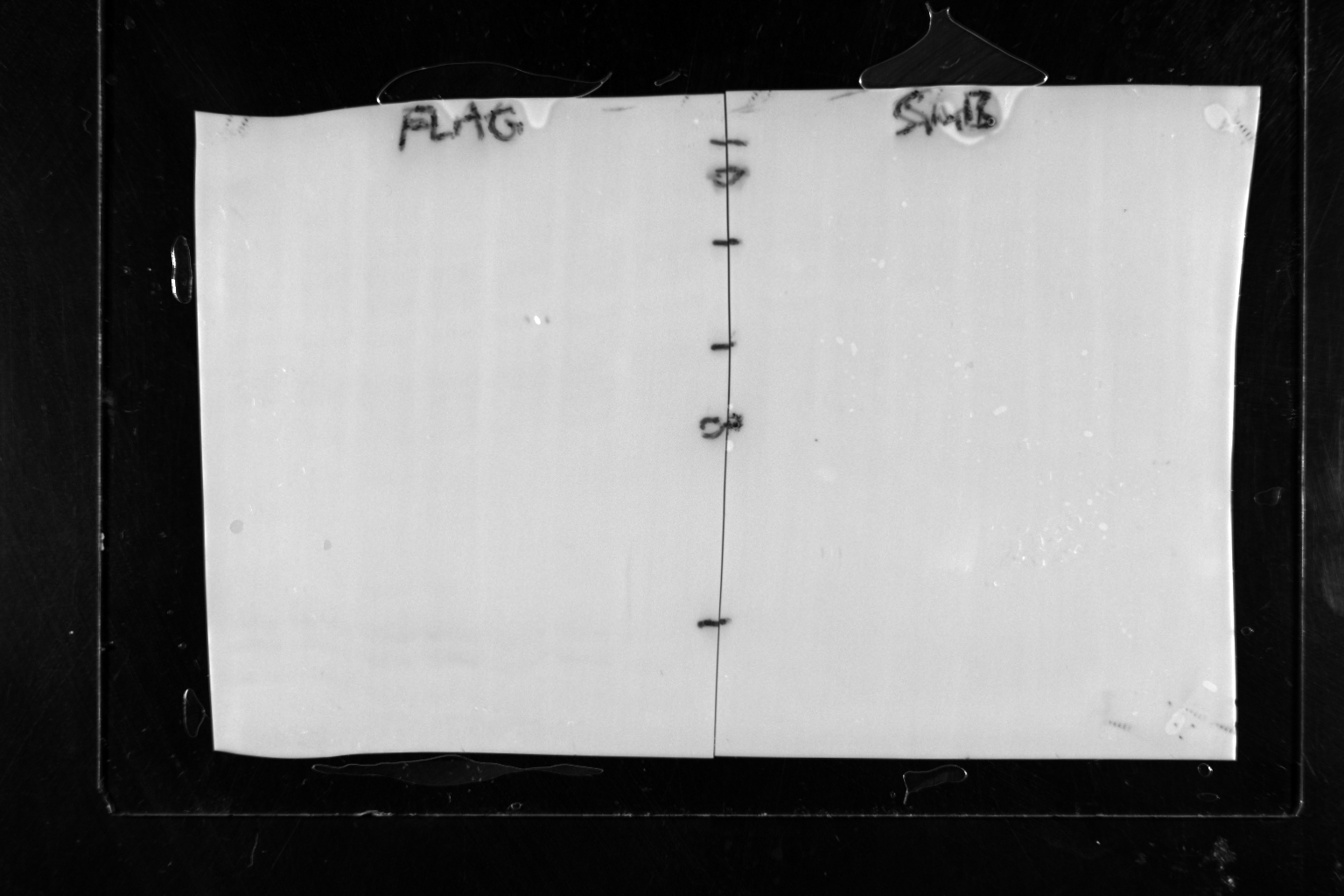

Supplement: Figure 5—figure supplement 1—source data 1. [file elife-72867-fig5-figsupp1-data1.zip › Figure5-figure_supplement_1-source data1/SmExp_CHTOPexp_FLAG_1.10000_SmB_1.2000_30sec_Standard_ladder.tif]

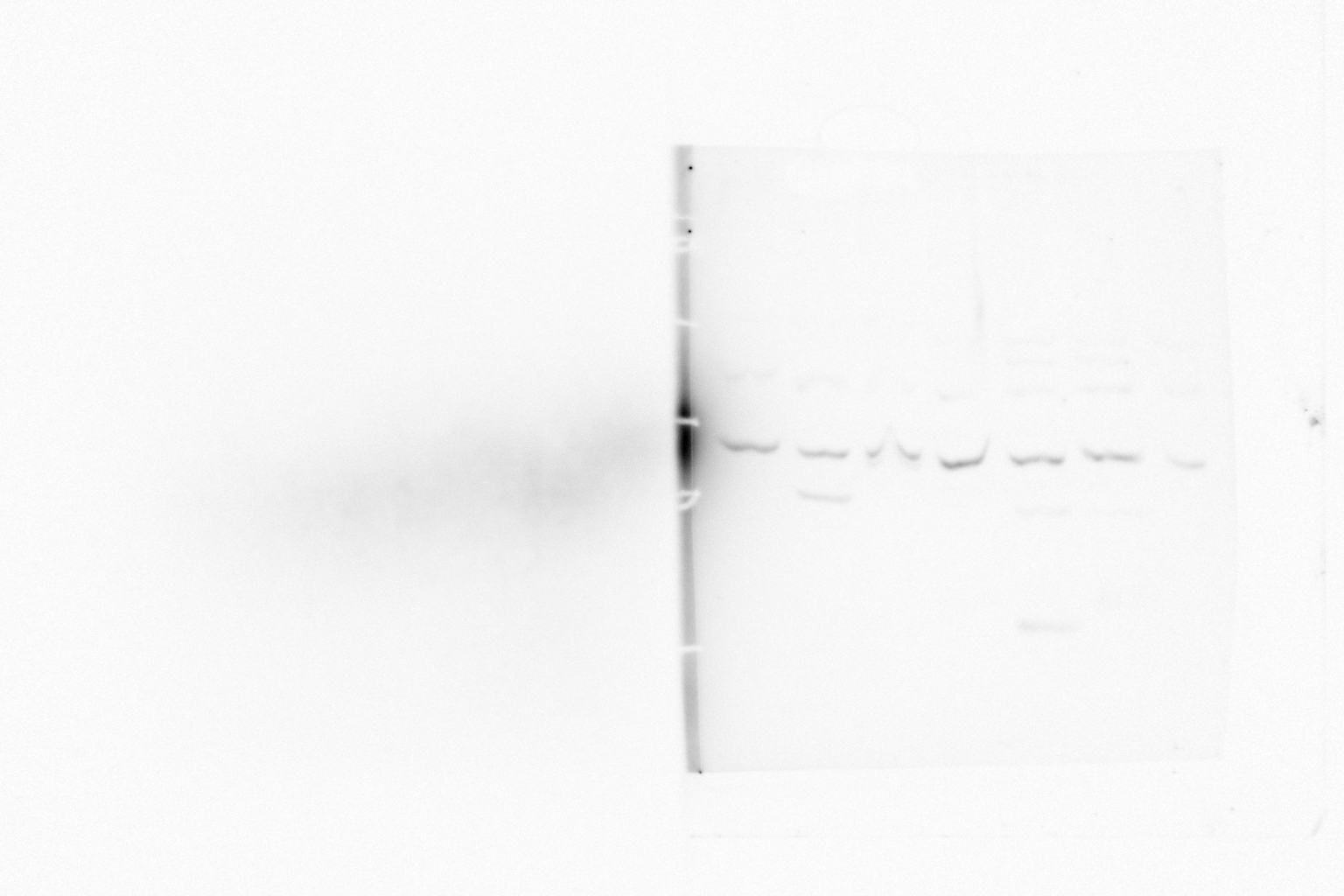

Supplement: Figure 5—figure supplement 1—source data 1. [file elife-72867-fig5-figsupp1-data1.zip › Figure5-figure_supplement_1-source data1/SmExp_CHTOPexp_GAPDH_1.10000_30sec_Standard_15.tif]

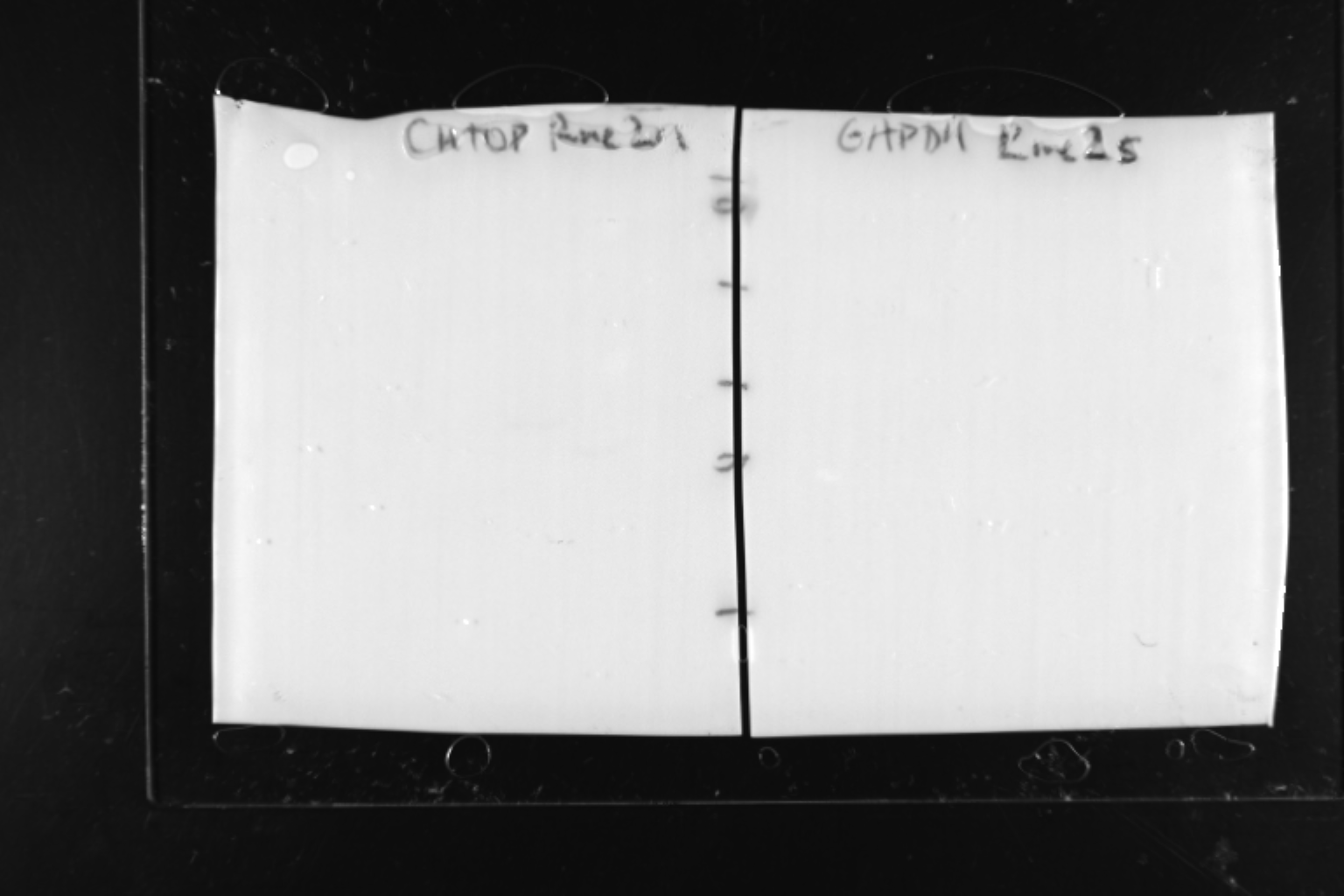

Supplement: Figure 5—figure supplement 1—source data 1. [file elife-72867-fig5-figsupp1-data1.zip › Figure5-figure_supplement_1-source data1/SmExp_CHTOPexp_Rme2a_1.2000_Rme2s_1.2000_120sec_ladder.tif]

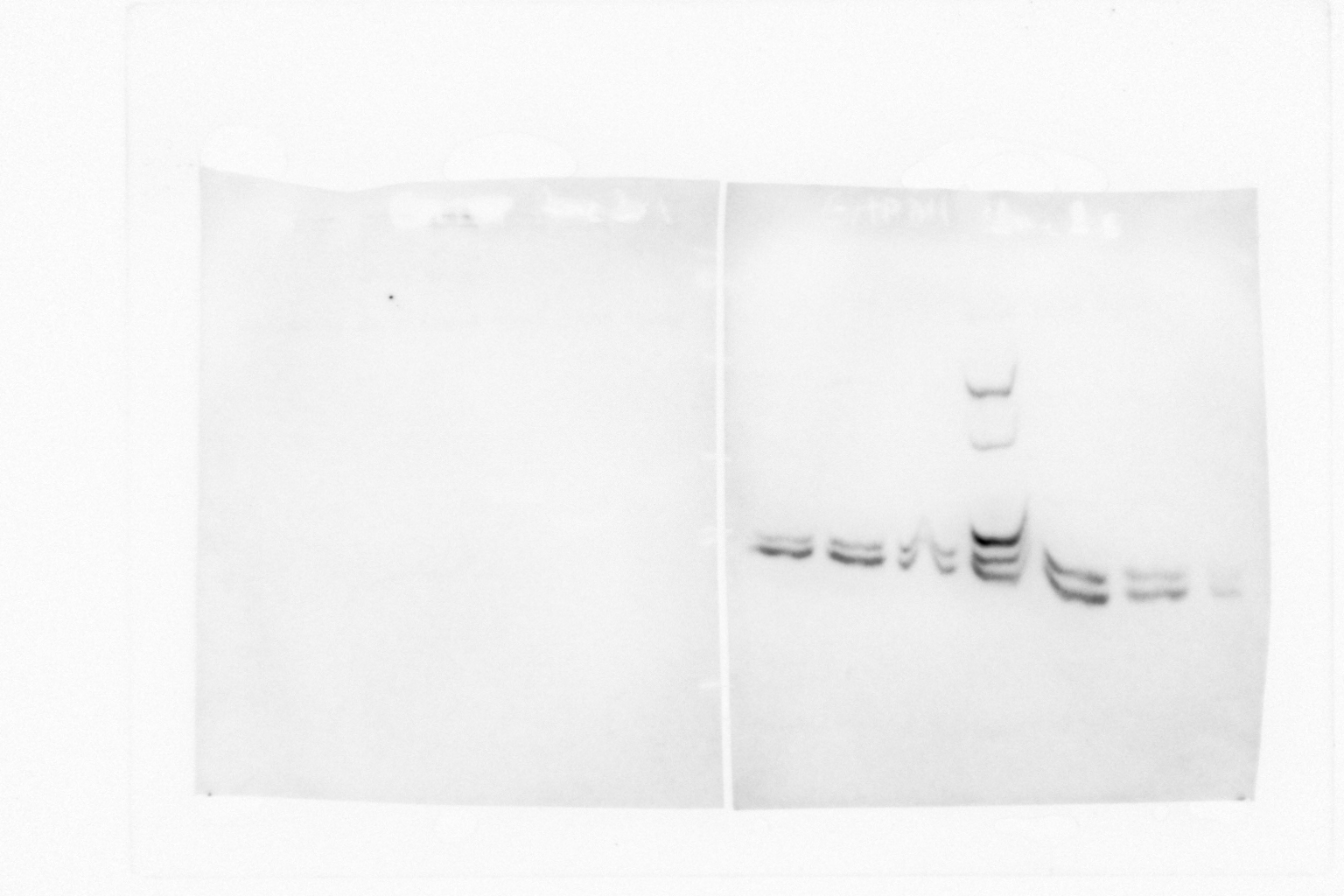

Supplement: Figure 5—figure supplement 1—source data 1. [file elife-72867-fig5-figsupp1-data1.zip › Figure5-figure_supplement_1-source data1/SmExp_CHTOPexp_Rme2a_1.2000_Rme2s_1.2000_30sec_Standard_10.tif]

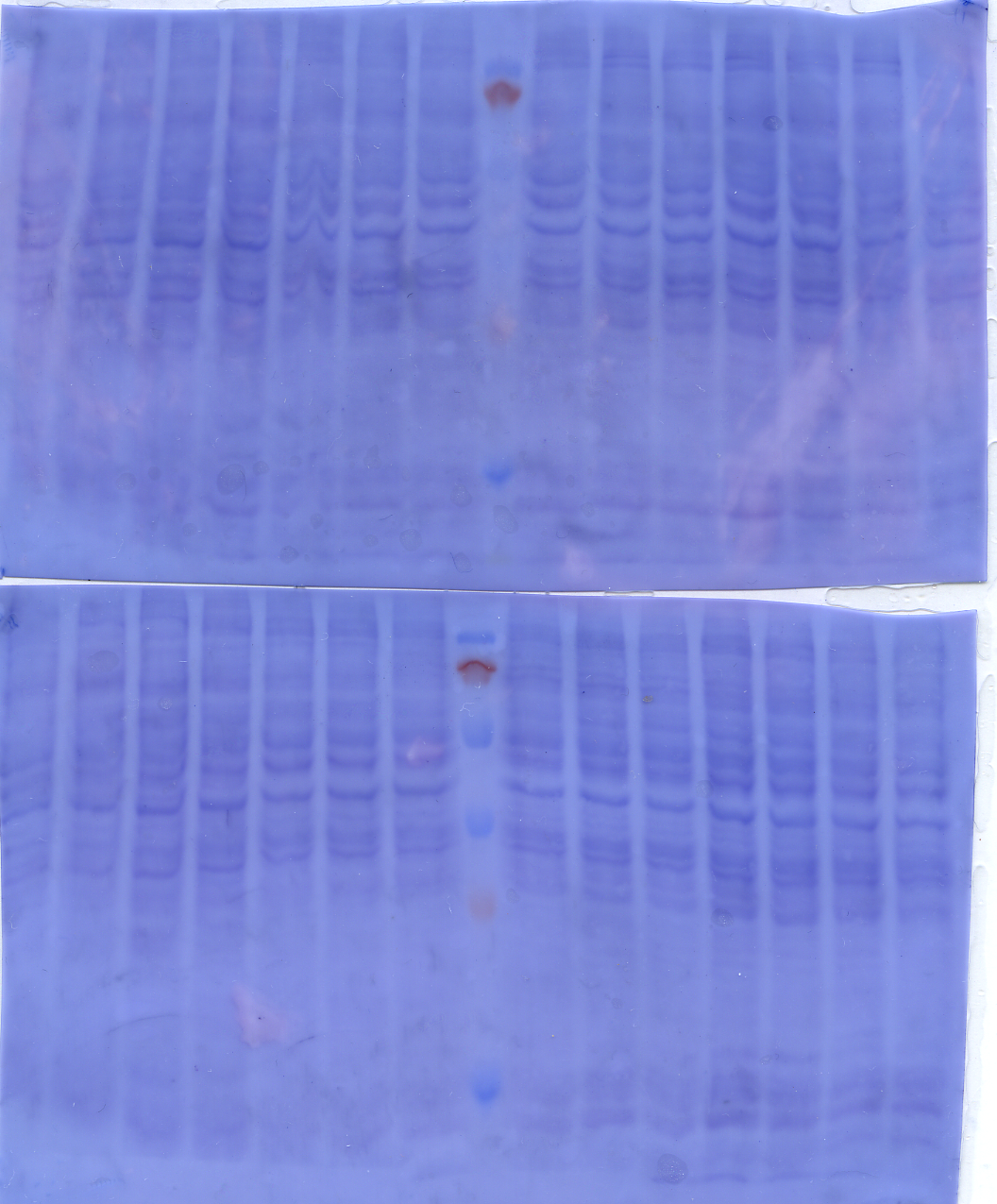

Supplement: Figure 5—figure supplement 1—source data 1. [file elife-72867-fig5-figsupp1-data1.zip › Figure5-figure_supplement_1-source data1/Sm_CHTOP_western_DirectBlue71.tif]

c

Chemiluminescence

Digital

Direct Blue 71 Membrane Stain

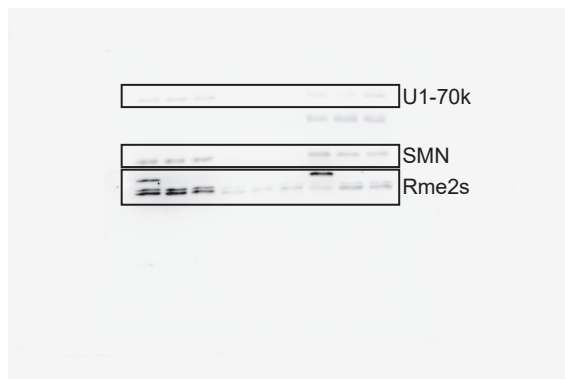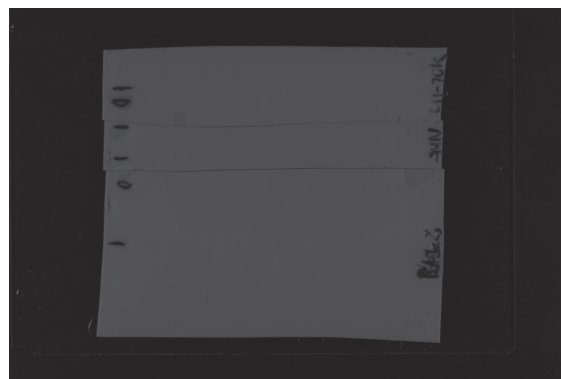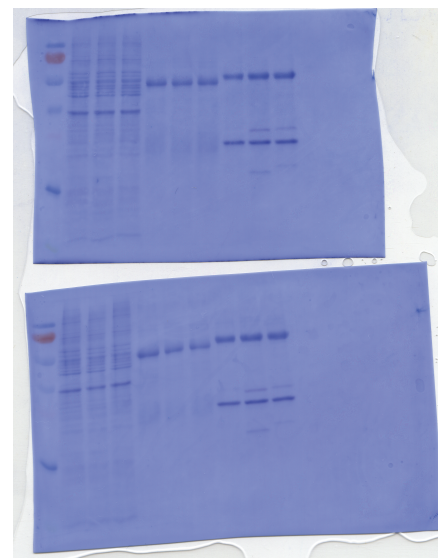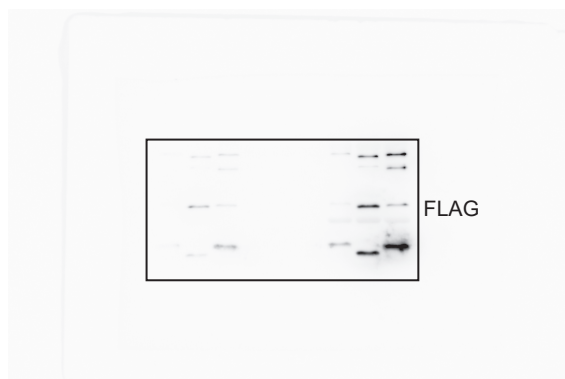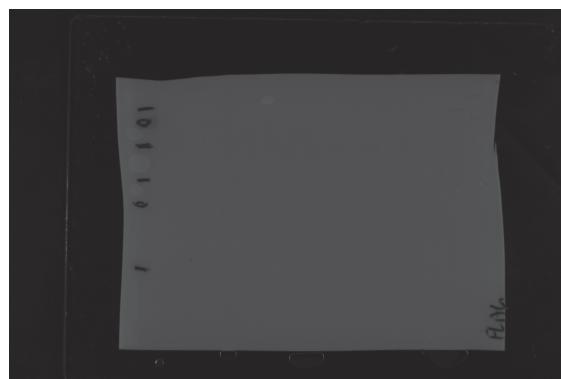

Supplement: Figure 5—figure supplement 2—source data 1. [file elife-72867-fig5-figsupp2-data1.zip › Figure5-figure_supplement_2-source data1/Figure 5-Figure Supplement 2-source data 1.pdf]

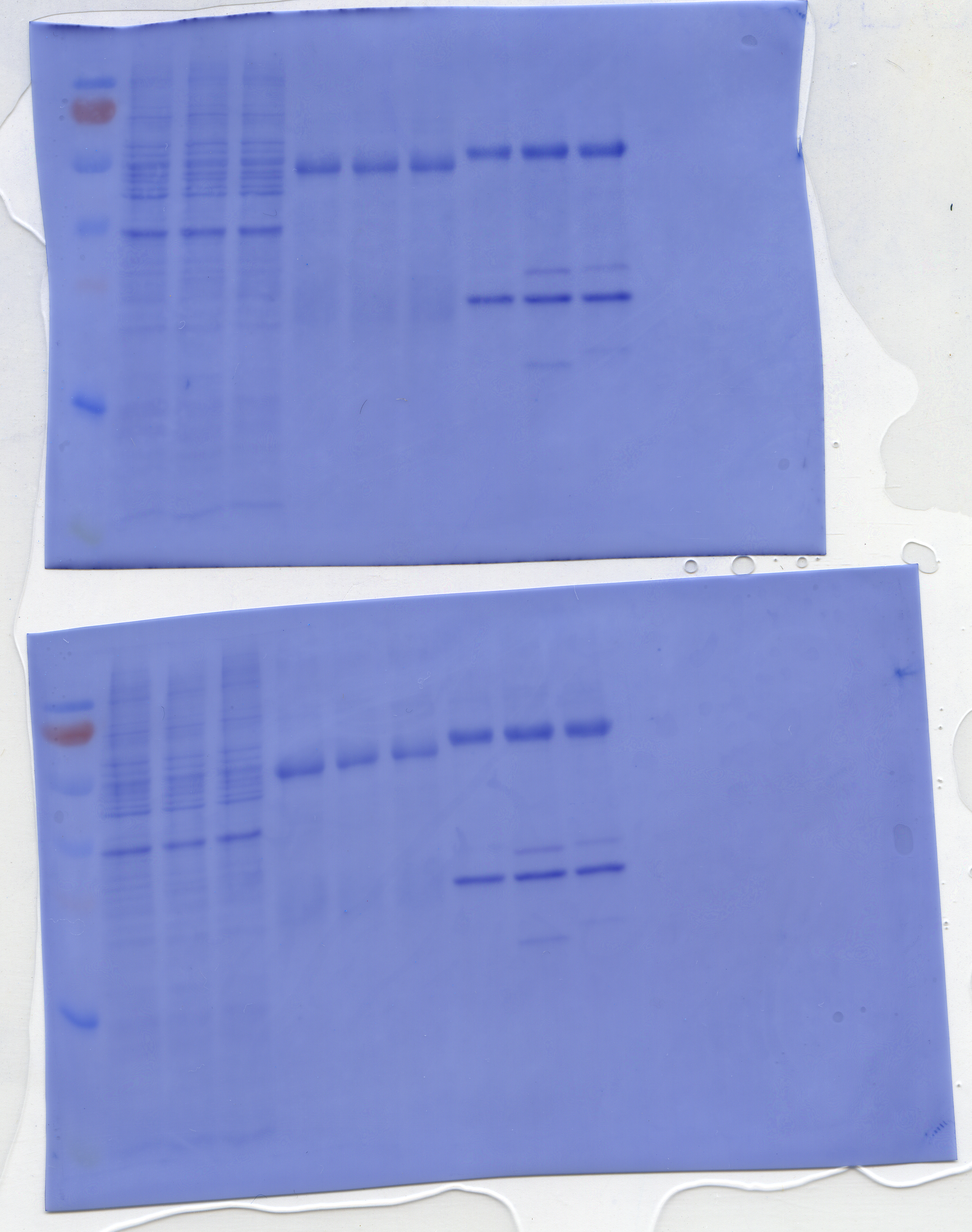

Supplement: Figure 5—figure supplement 2—source data 1. [file elife-72867-fig5-figsupp2-data1.zip › Figure5-figure_supplement_2-source data1/SmExp_FLAG_CoIP_DirectBlue.tif]

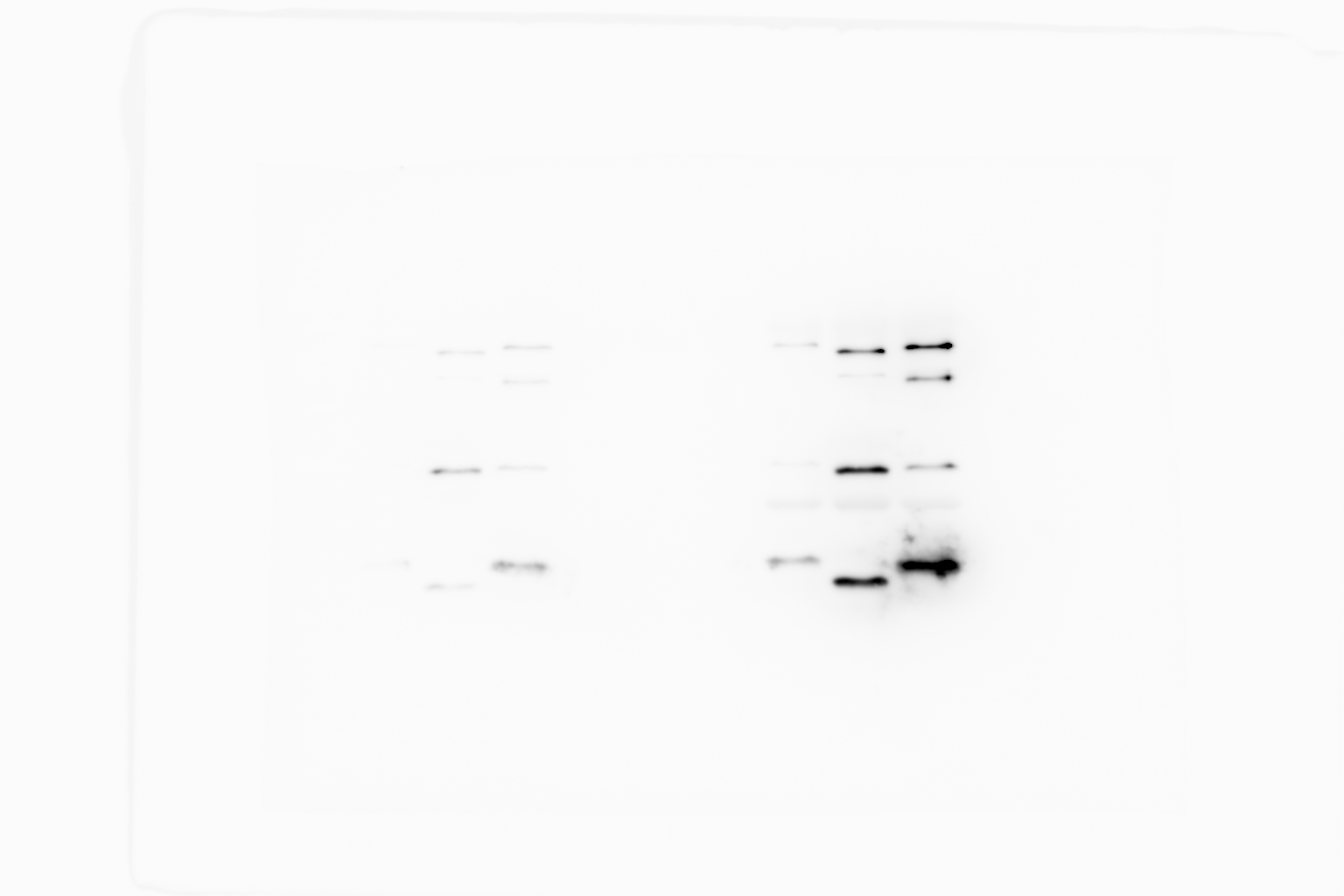

Supplement: Figure 5—figure supplement 2—source data 1. [file elife-72867-fig5-figsupp2-data1.zip › Figure5-figure_supplement_2-source data1/SmExp_FLAG_CoIP_FLAG_1.2000_30sec_Std_10.tif]

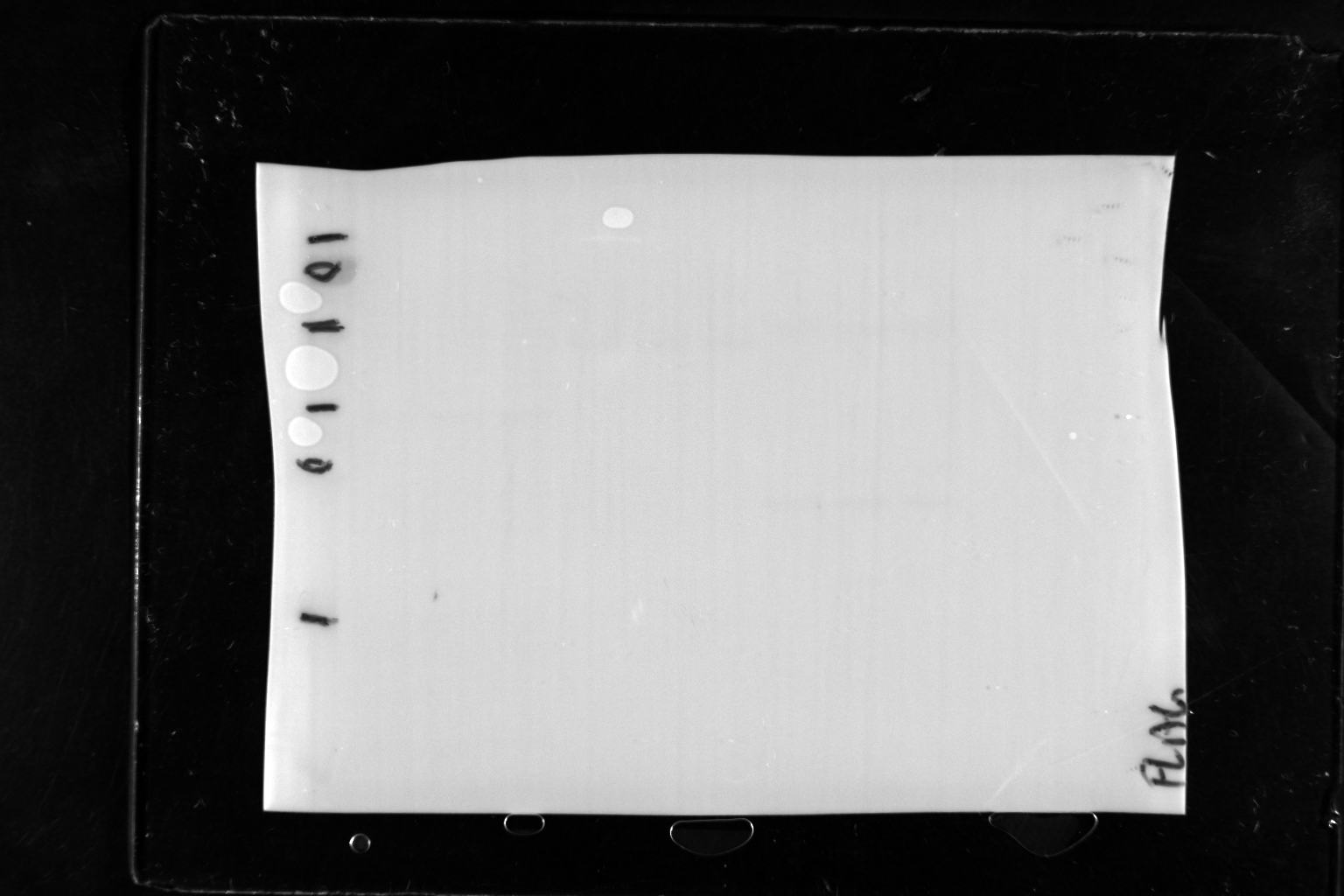

Supplement: Figure 5—figure supplement 2—source data 1. [file elife-72867-fig5-figsupp2-data1.zip › Figure5-figure_supplement_2-source data1/SmExp_FLAG_CoIP_FLAG_1.2000_30sec_Std_ladder.tif]

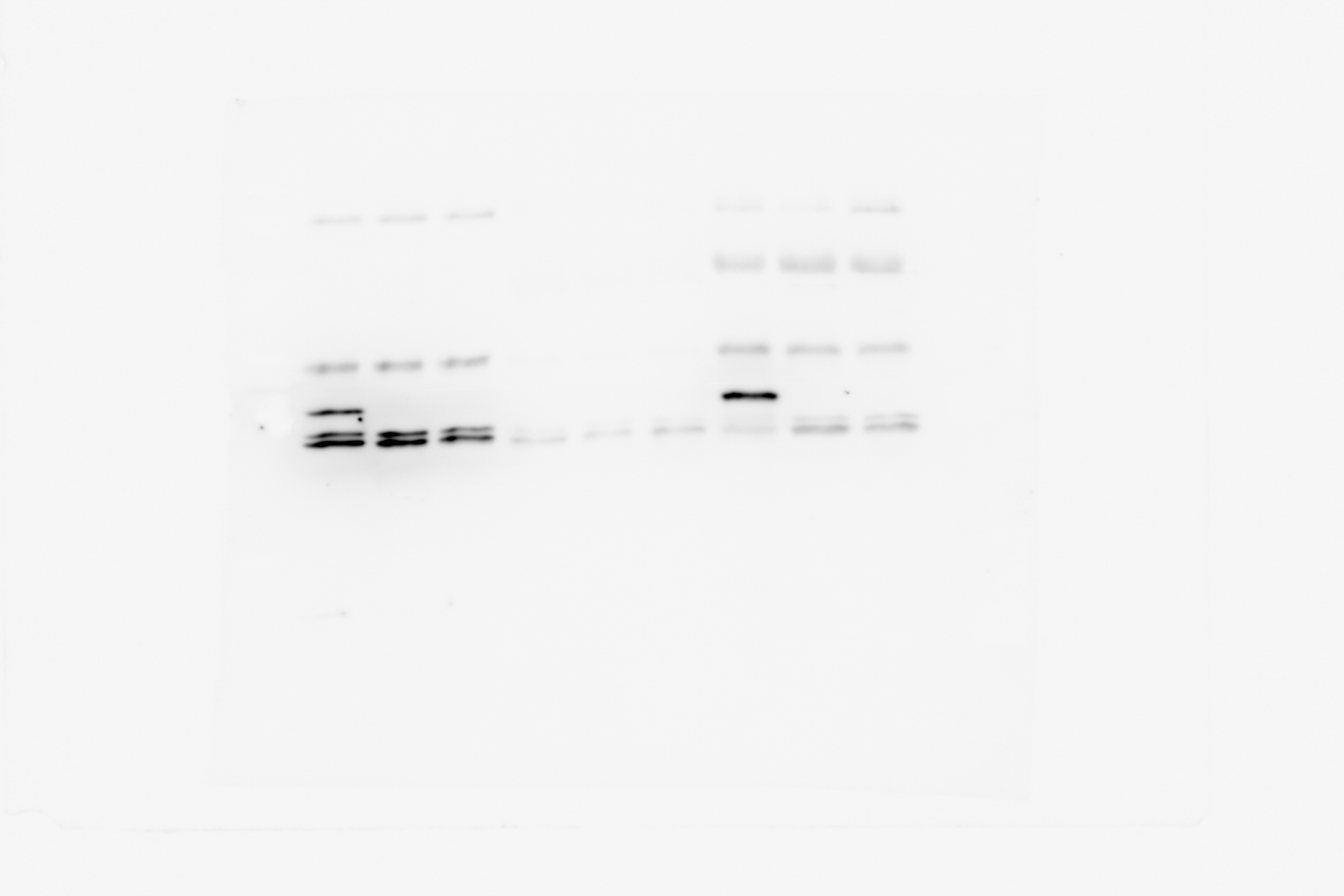

Supplement: Figure 5—figure supplement 2—source data 1. [file elife-72867-fig5-figsupp2-data1.zip › Figure5-figure_supplement_2-source data1/SmExp_FLAG_CoIP_Rme2s_1.2000_SMN_1.2000_U170k_1.2000_30sec_Std_10.tif]

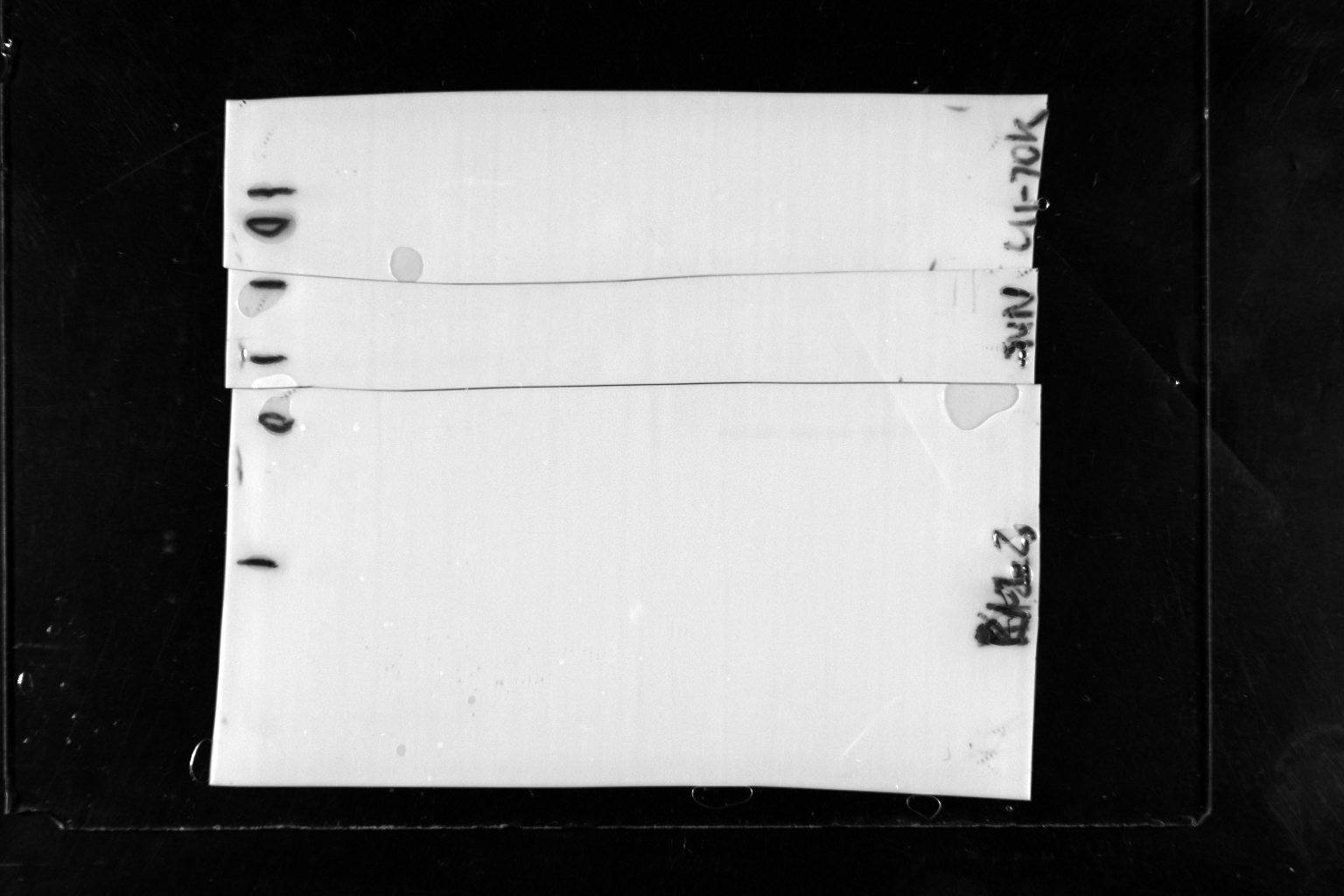

Supplement: Figure 5—figure supplement 2—source data 1. [file elife-72867-fig5-figsupp2-data1.zip › Figure5-figure_supplement_2-source data1/SmExp_FLAG_CoIP_Rme2s_1.2000_SMN_1.2000_U170k_1.2000_30sec_Std_ladder.tif]

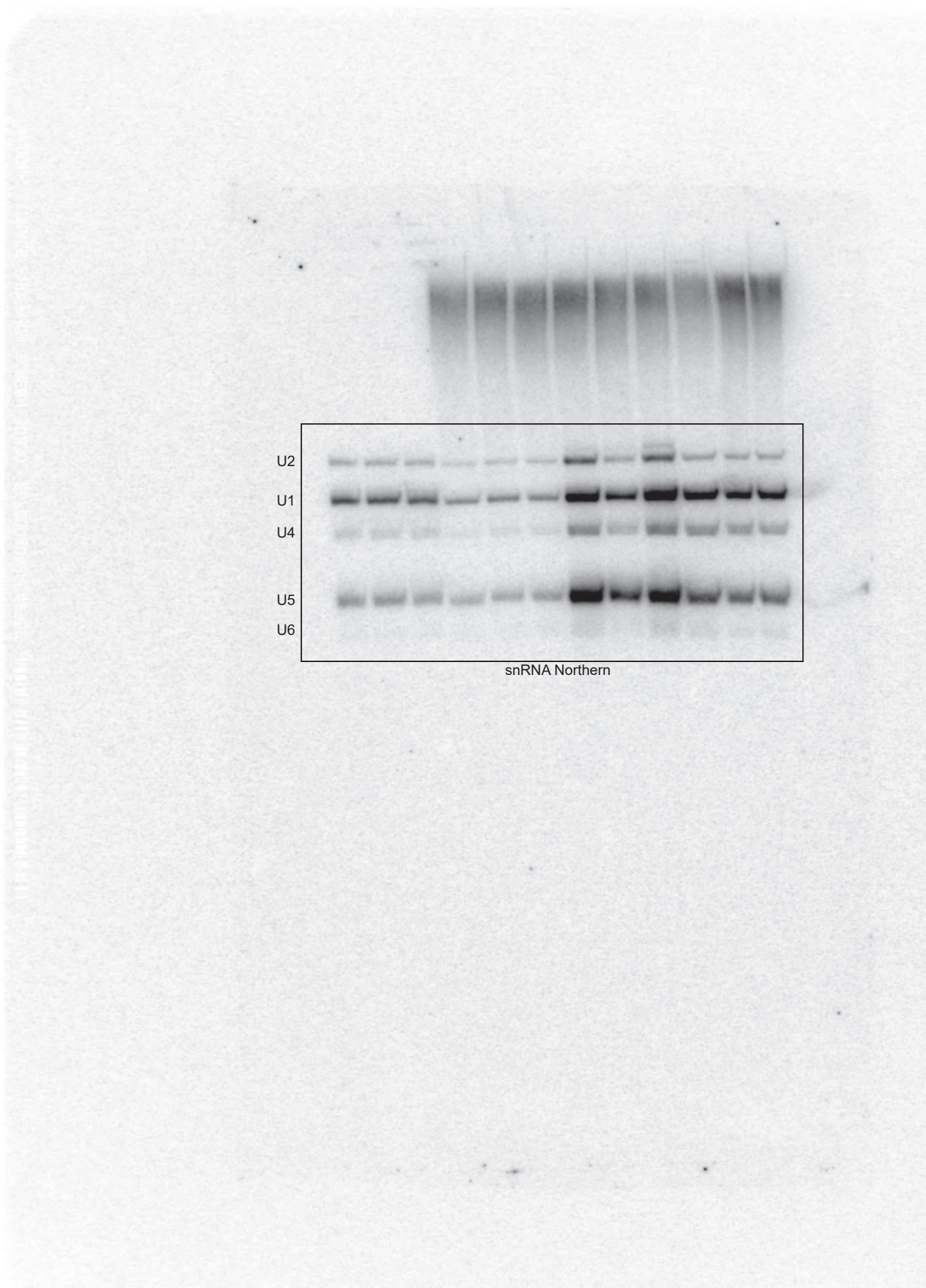

Supplement: Figure 5—figure supplement 2—source data 2. [file elife-72867-fig5-figsupp2-data2.zip › Figure5-figure_supplement_2-source data2/Figure 5-Figure Supplement 2-source data 2.pdf]

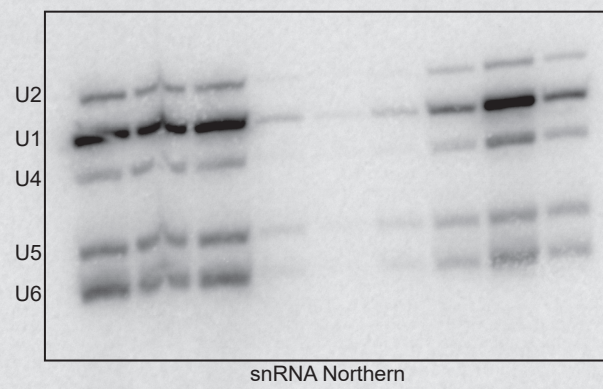

Supplement: Figure 5—figure supplement 2—source data 3. [file elife-72867-fig5-figsupp2-data3.zip › Figure5-figure_supplement_2-source data3/Figure 5-Figure Supplement 2-source data 3.pdf]

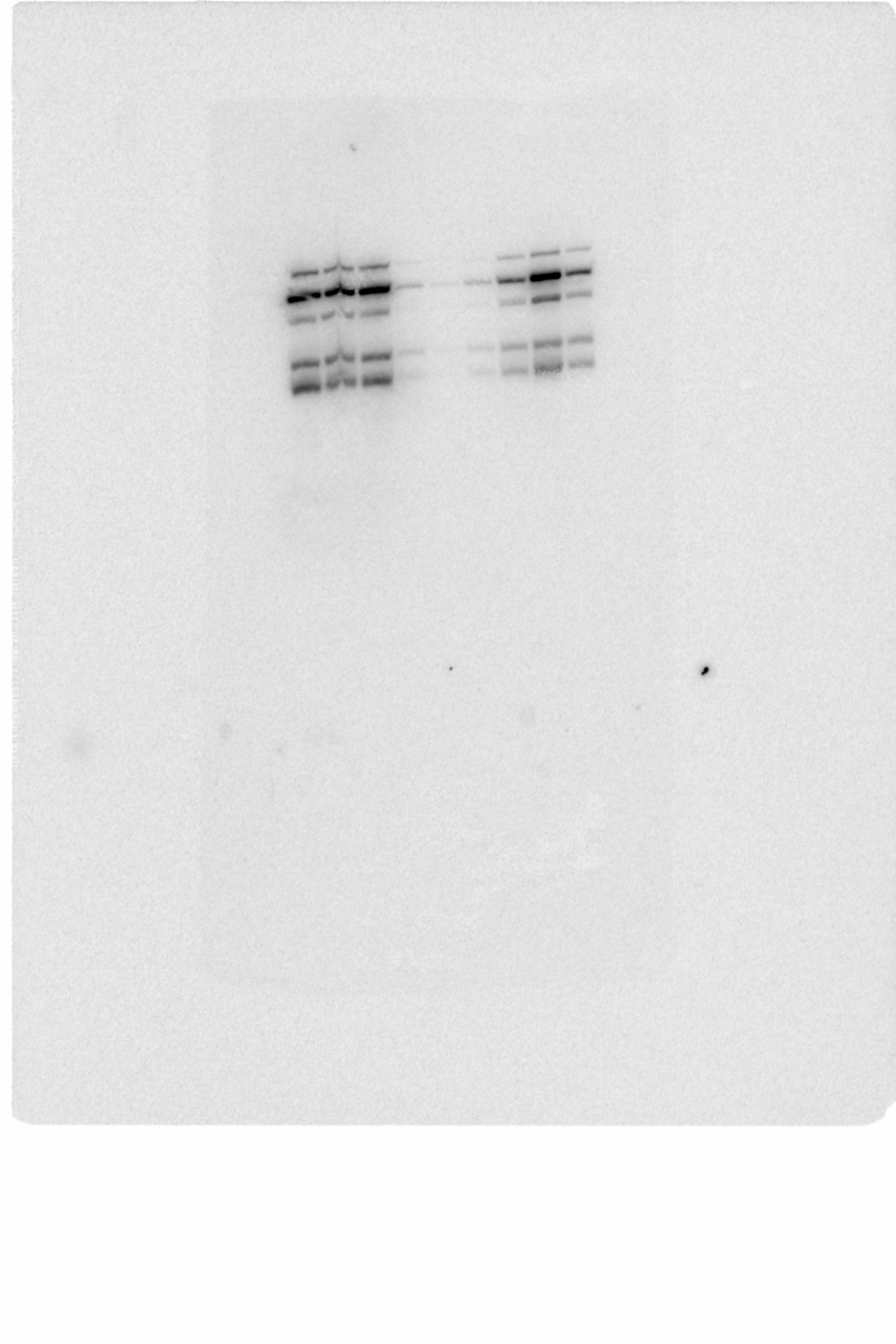

Supplement: Figure 5—figure supplement 2—source data 3. [file elife-72867-fig5-figsupp2-data3.zip › Figure5-figure_supplement_2-source data3/PRMTi_SmB_RIP_5dayExposure.gel]

**d**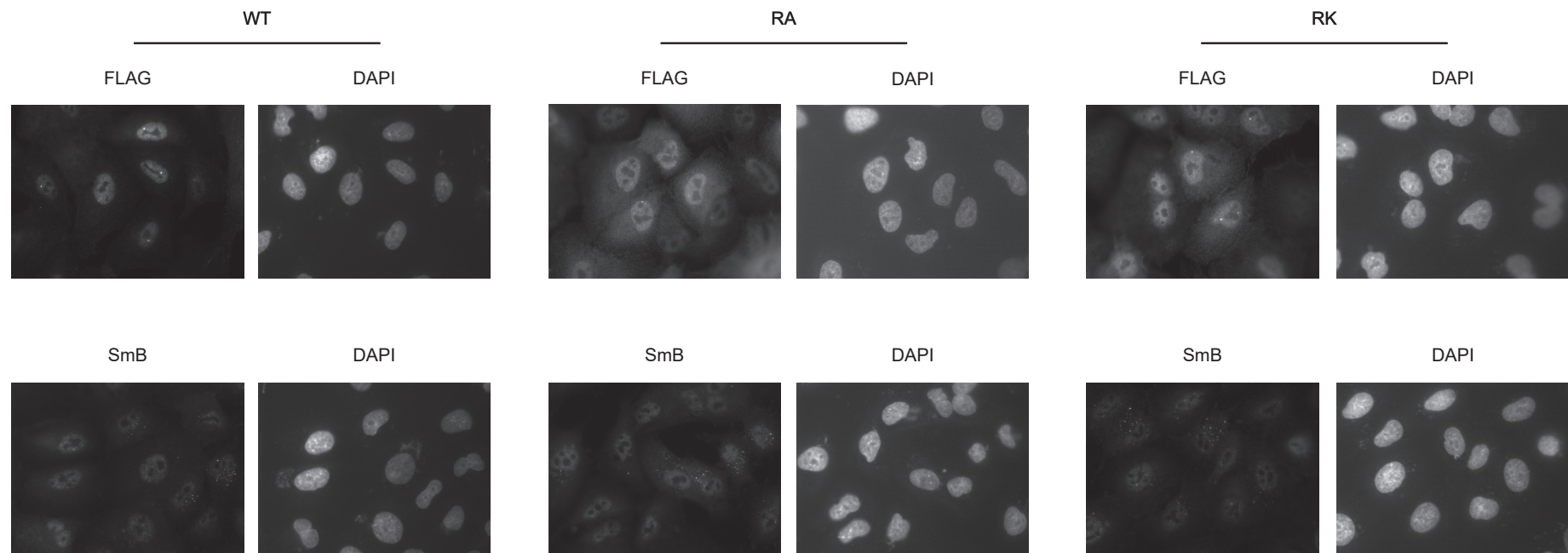

Supplement: Figure 5—figure supplement 2—source data 4. [file elife-72867-fig5-figsupp2-data4.zip › Figure5-figure_supplement_2-source data4/Figure5-figure_supplement_2-data4.pdf]

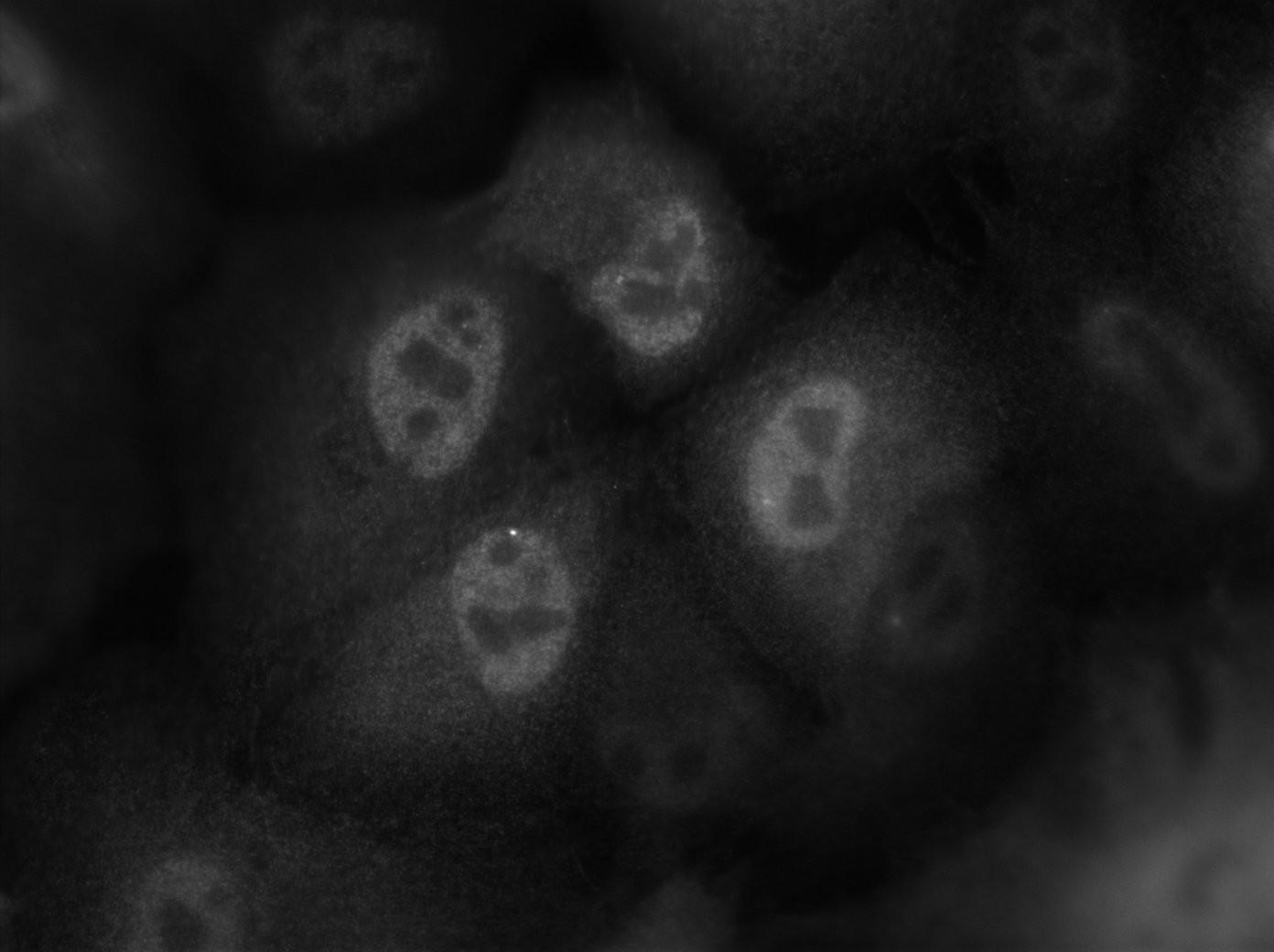

Supplement: Figure 5—figure supplement 2—source data 4. [file elife-72867-fig5-figsupp2-data4.zip › Figure5-figure_supplement_2-source data4/SmRA_FLAG_555_60x_50_500ms_03.jpg]

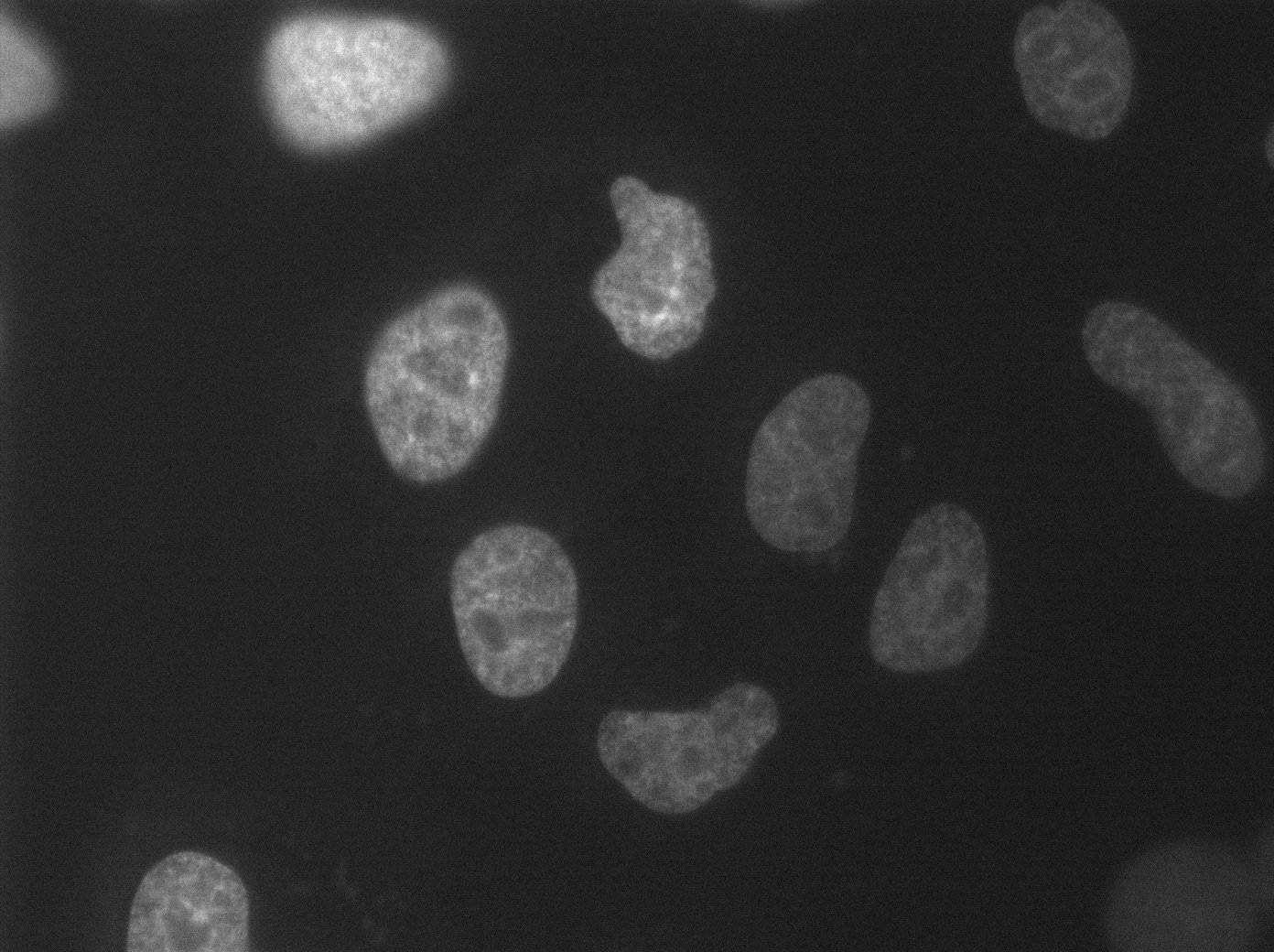

Supplement: Figure 5—figure supplement 2—source data 4. [file elife-72867-fig5-figsupp2-data4.zip › Figure5-figure_supplement_2-source data4/SmRA_FLAG_DAPI_60x_50_50ms_03.jpg]

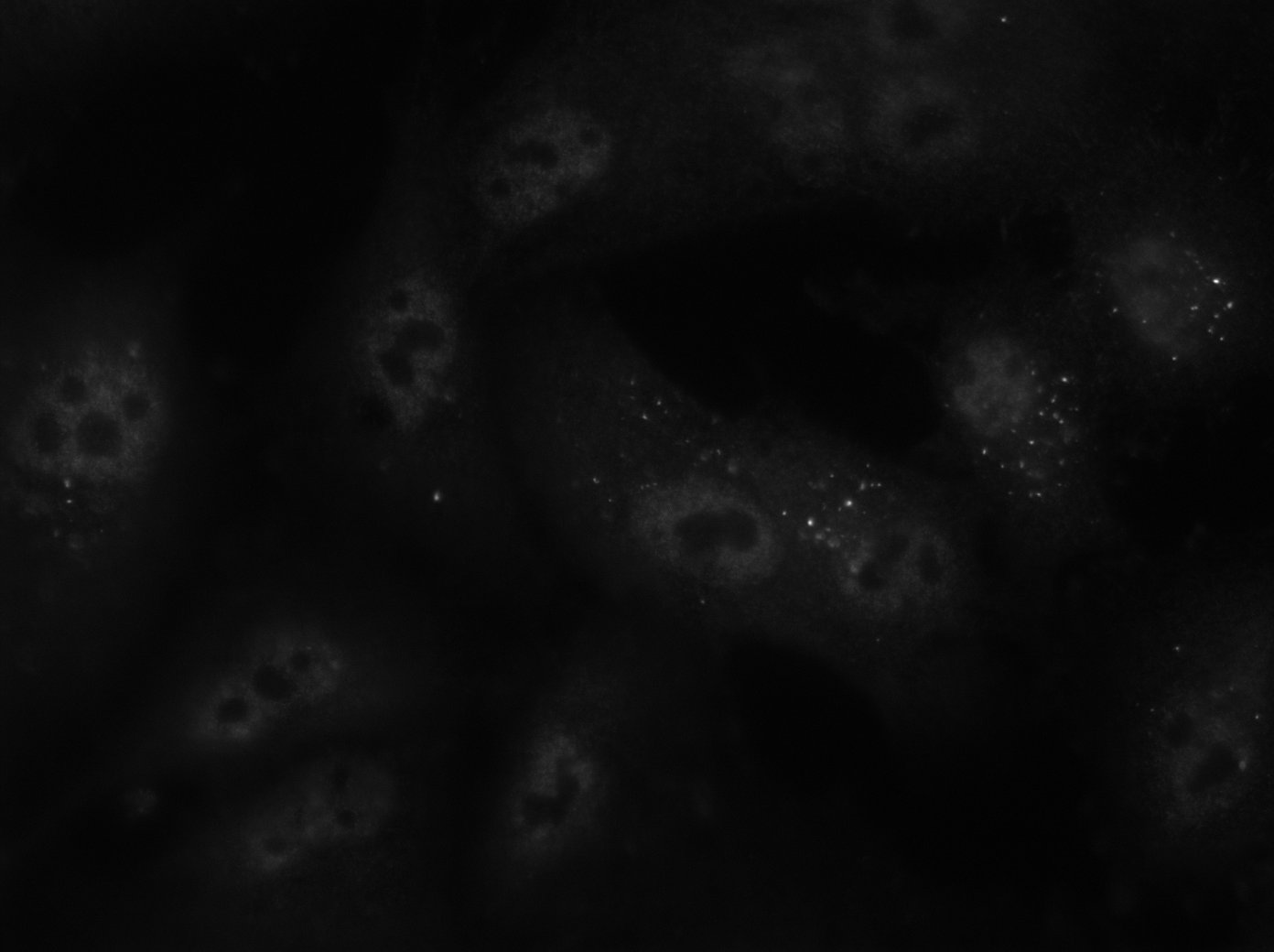

Supplement: Figure 5—figure supplement 2—source data 4. [file elife-72867-fig5-figsupp2-data4.zip › Figure5-figure_supplement_2-source data4/SmRA_SmB_488_60x_125_500ms_01.jpg]

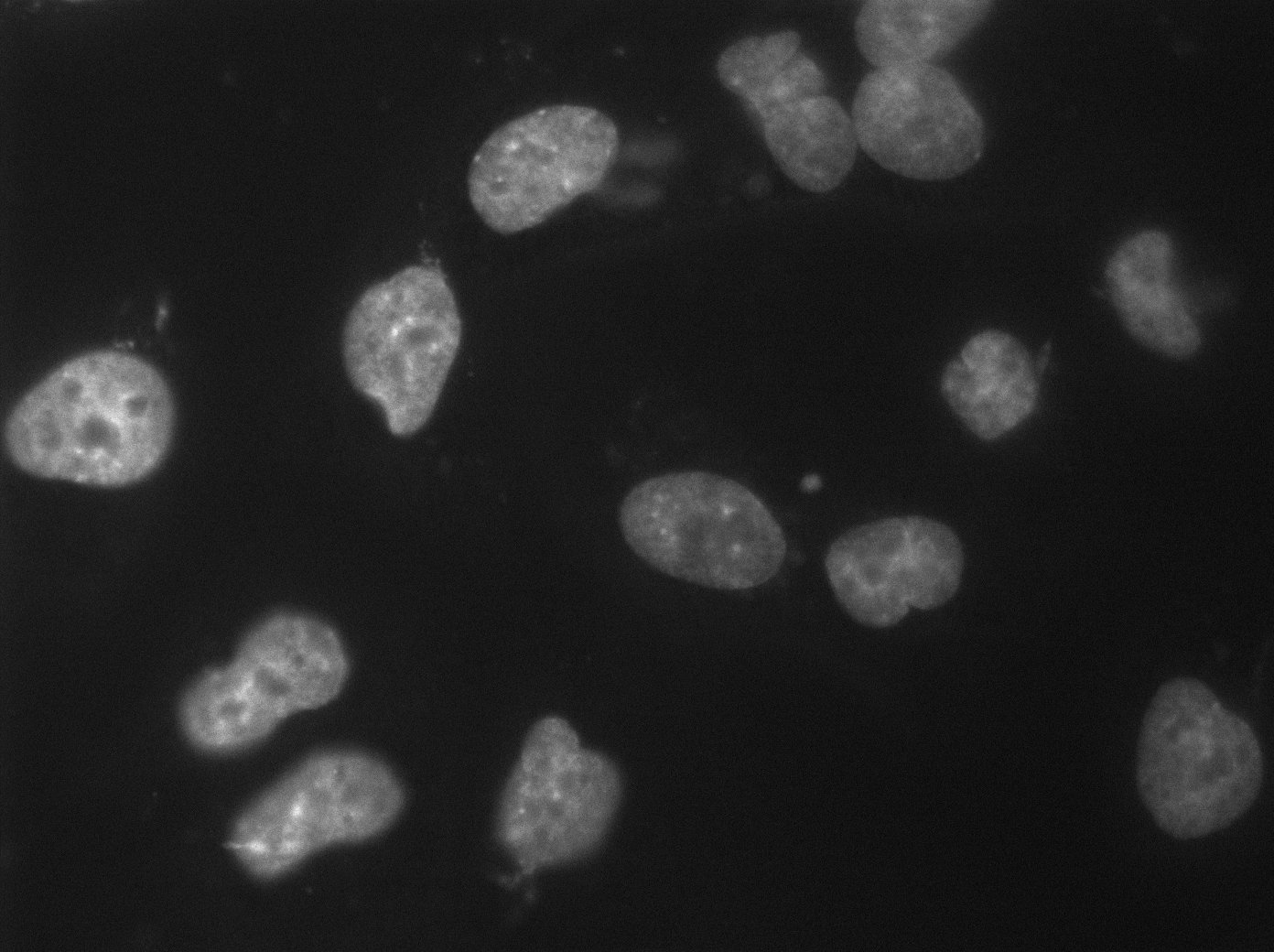

Supplement: Figure 5—figure supplement 2—source data 4. [file elife-72867-fig5-figsupp2-data4.zip › Figure5-figure_supplement_2-source data4/SmRA_SmB_DAPI_60x_125_50ms_01.jpg]

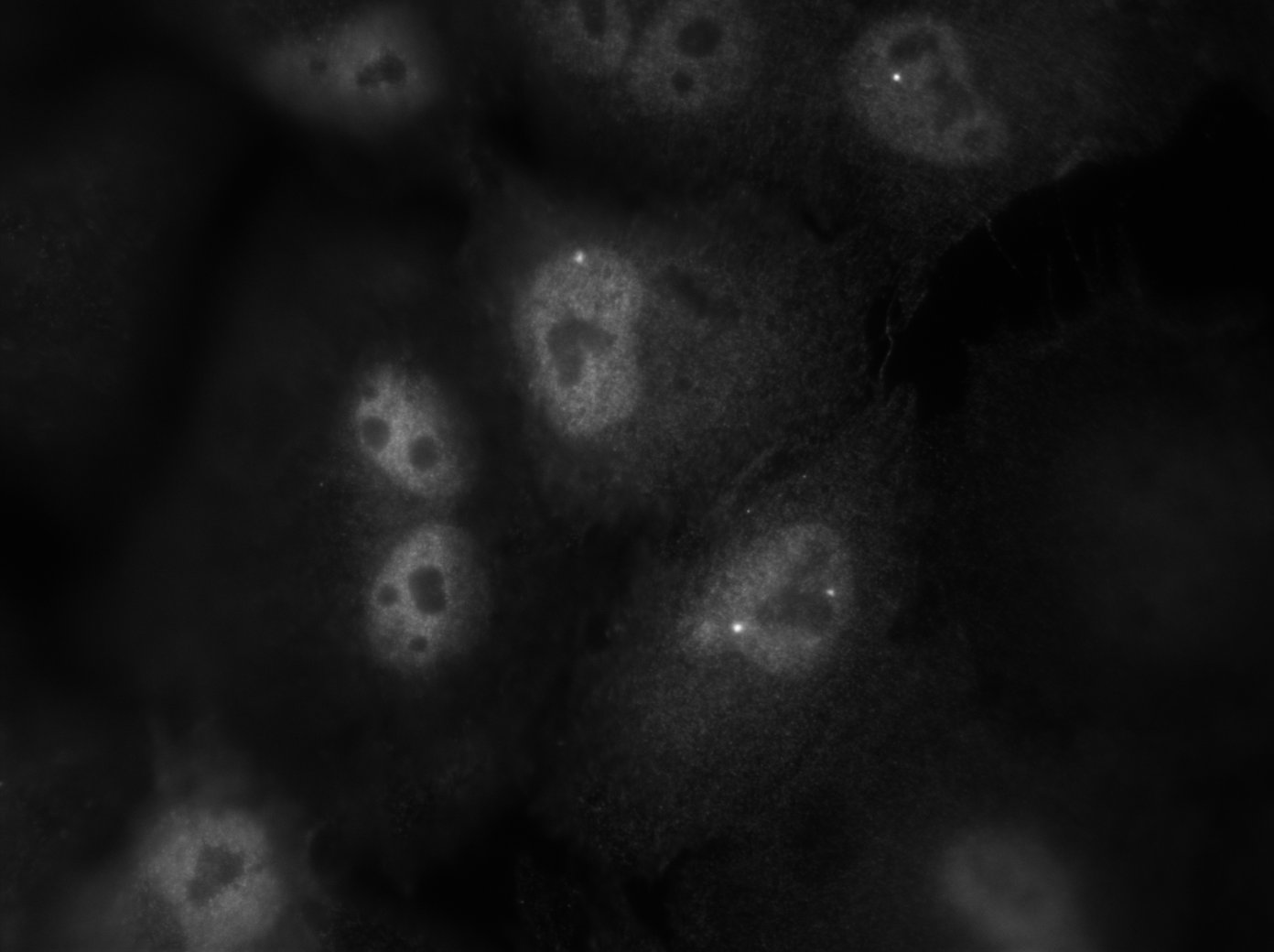

Supplement: Figure 5—figure supplement 2—source data 4. [file elife-72867-fig5-figsupp2-data4.zip › Figure5-figure_supplement_2-source data4/SmRK_FLAG_555_60x_50_500ms_05.jpg]

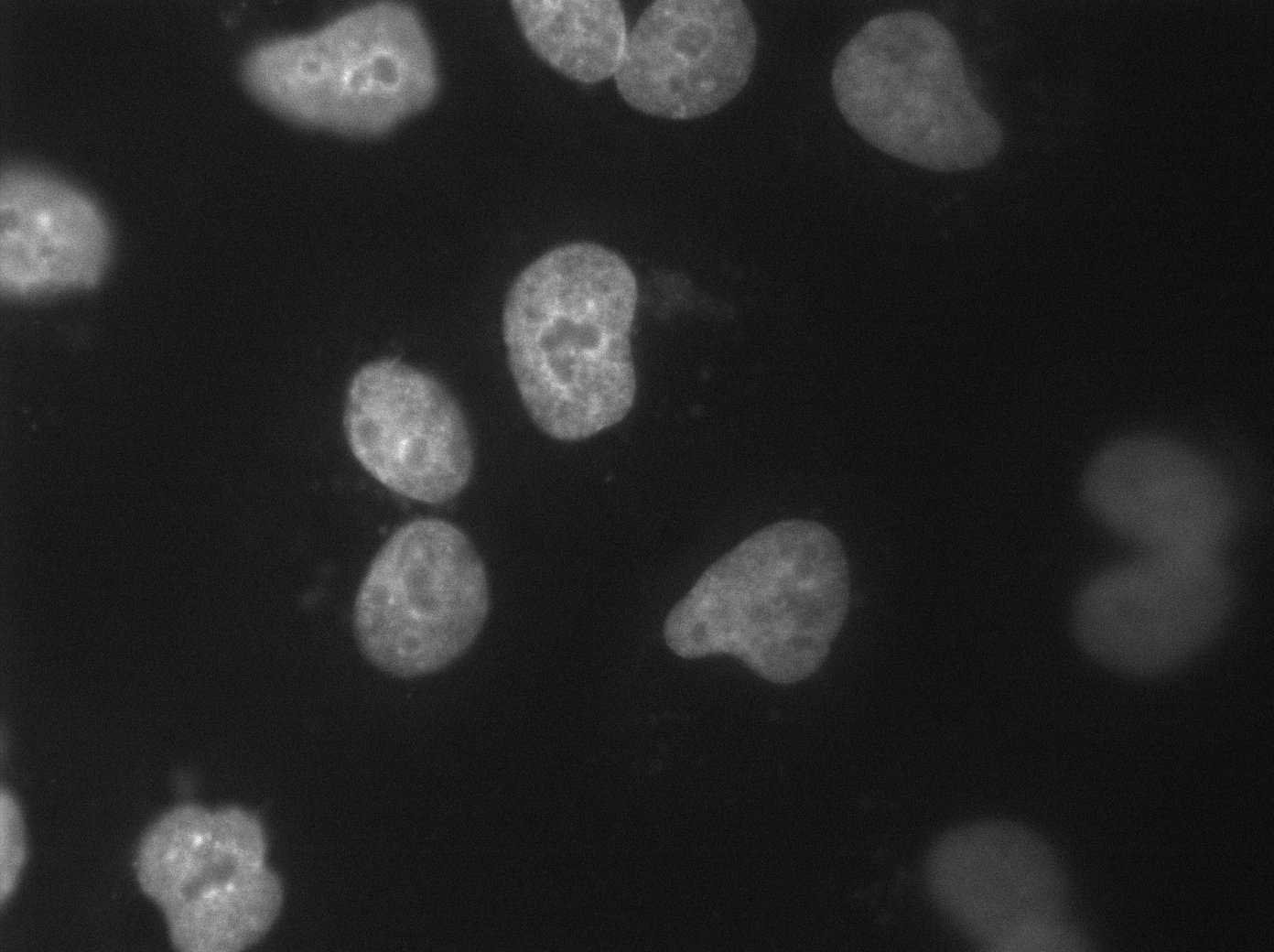

Supplement: Figure 5—figure supplement 2—source data 4. [file elife-72867-fig5-figsupp2-data4.zip › Figure5-figure_supplement_2-source data4/SmRK_FLAG_DAPI_60x_50_50ms_05.jpg]

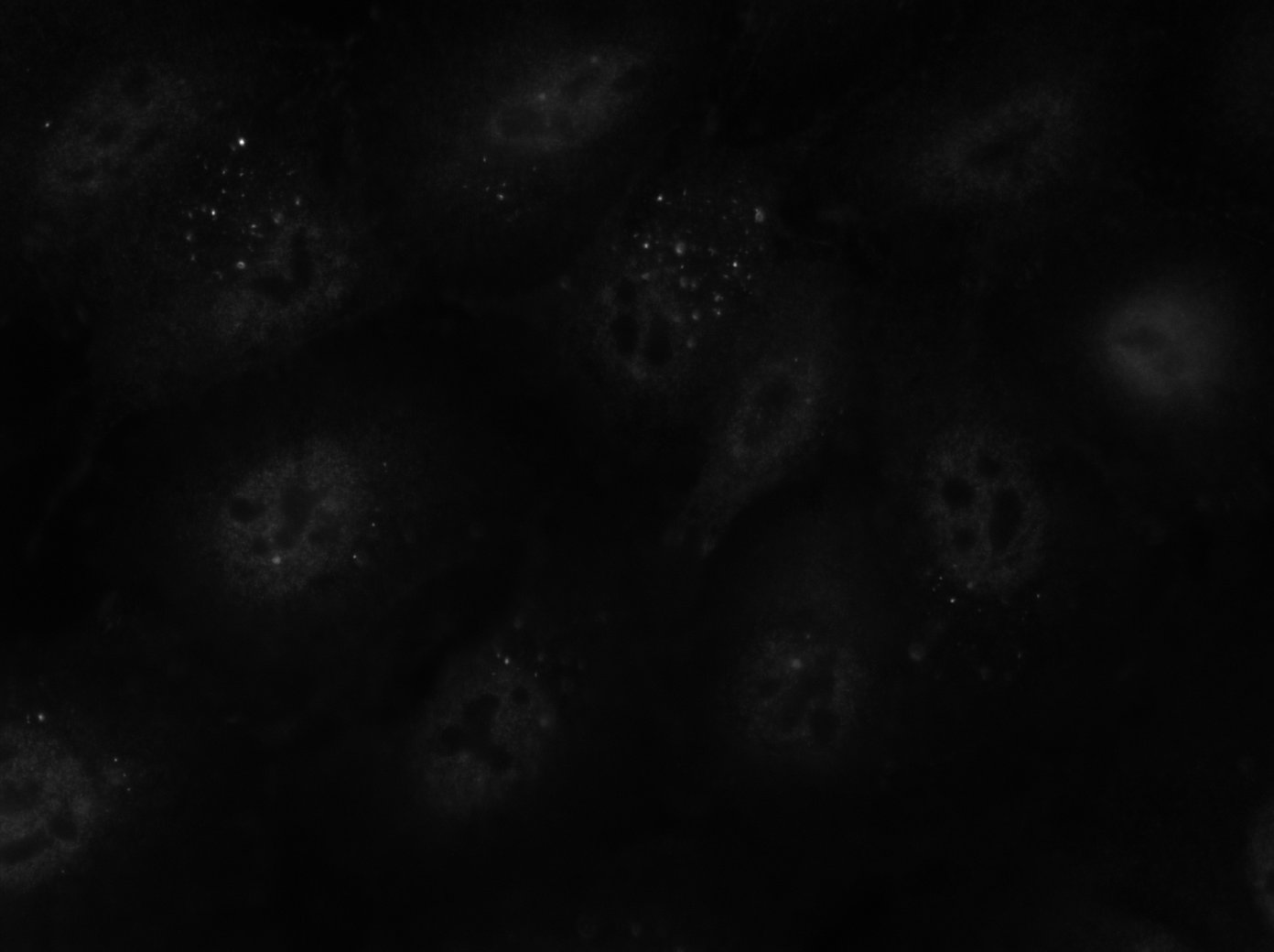

Supplement: Figure 5—figure supplement 2—source data 4. [file elife-72867-fig5-figsupp2-data4.zip › Figure5-figure_supplement_2-source data4/SmRK_SmB_488_60x_125_500ms_01.jpg]

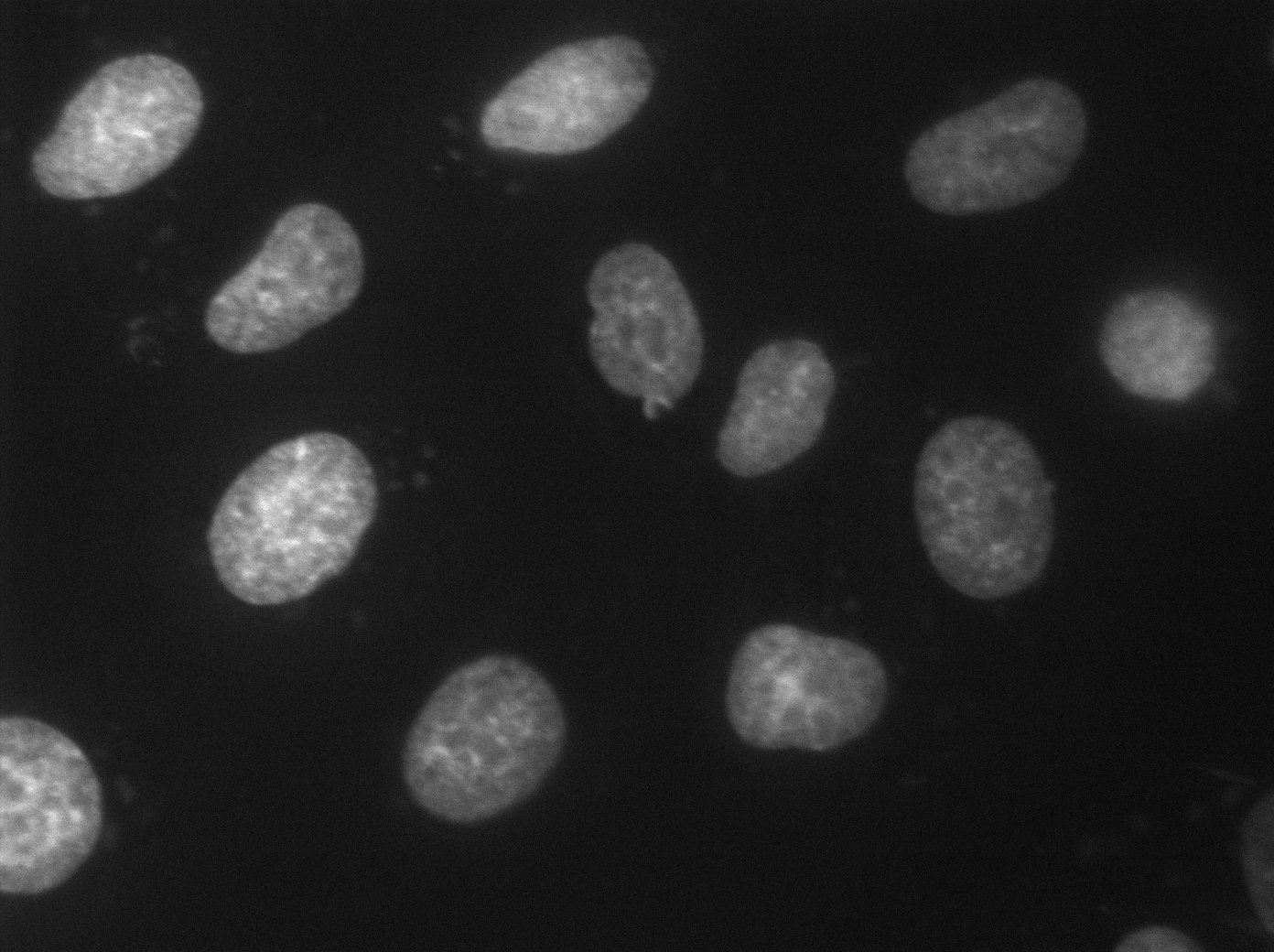

Supplement: Figure 5—figure supplement 2—source data 4. [file elife-72867-fig5-figsupp2-data4.zip › Figure5-figure_supplement_2-source data4/SmRK_SmB_DAPI_60x_125_50ms_01.jpg]

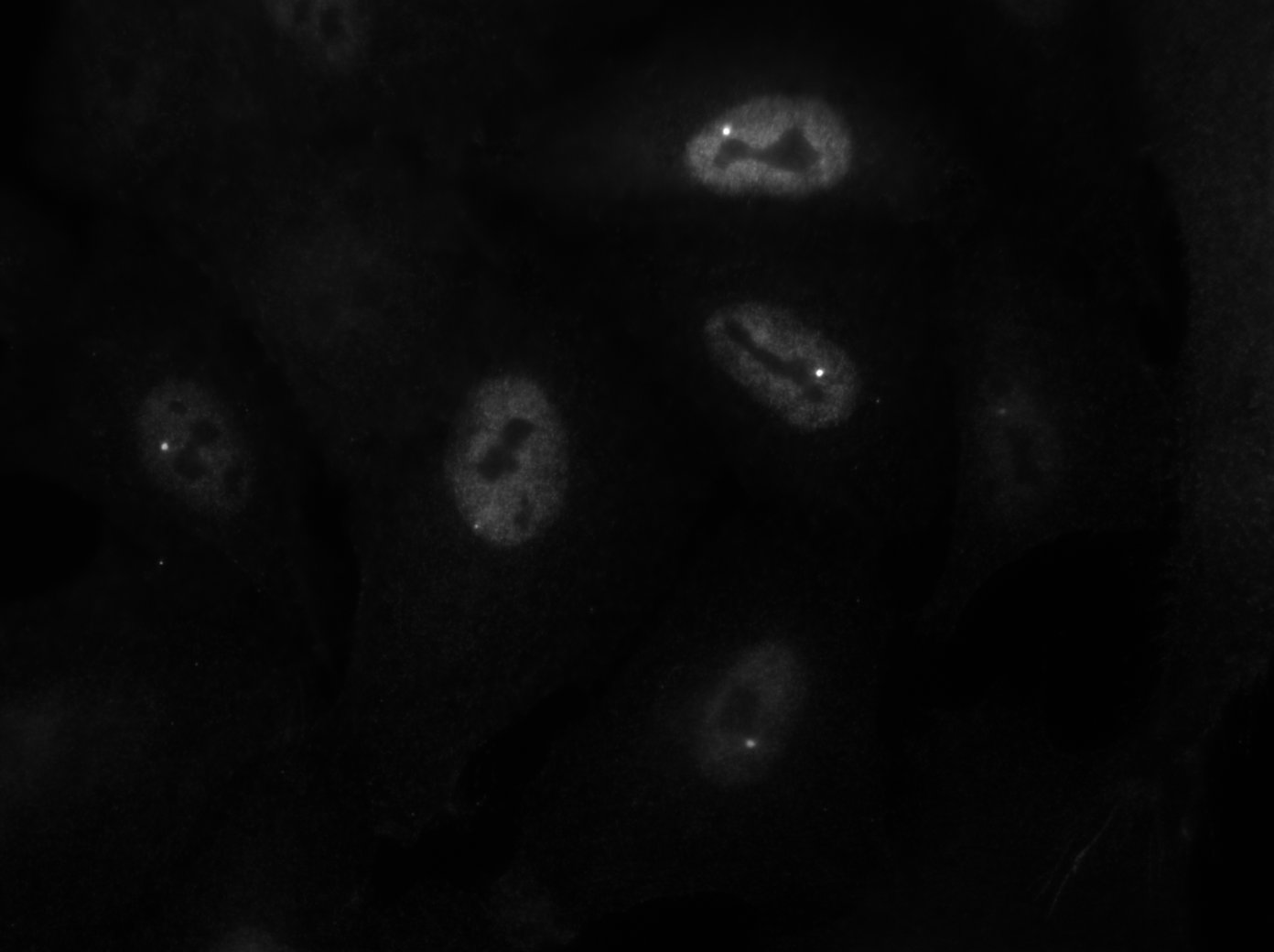

Supplement: Figure 5—figure supplement 2—source data 4. [file elife-72867-fig5-figsupp2-data4.zip › Figure5-figure_supplement_2-source data4/SmWT_FLAG_555_60x_50_500ms_05.jpg]

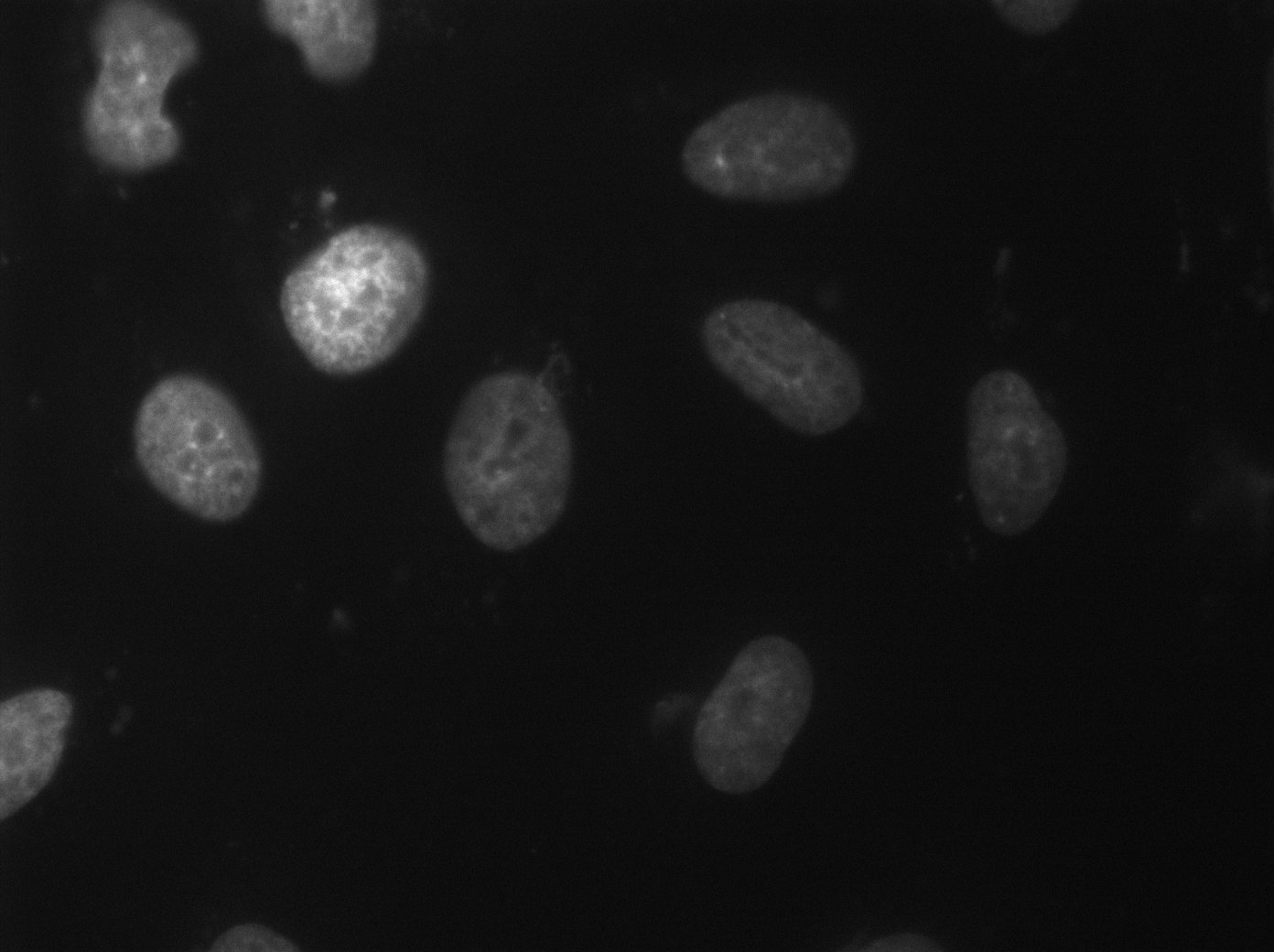

Supplement: Figure 5—figure supplement 2—source data 4. [file elife-72867-fig5-figsupp2-data4.zip › Figure5-figure_supplement_2-source data4/SmWT_FLAG_DAPI_60x_50_50ms_05.jpg]

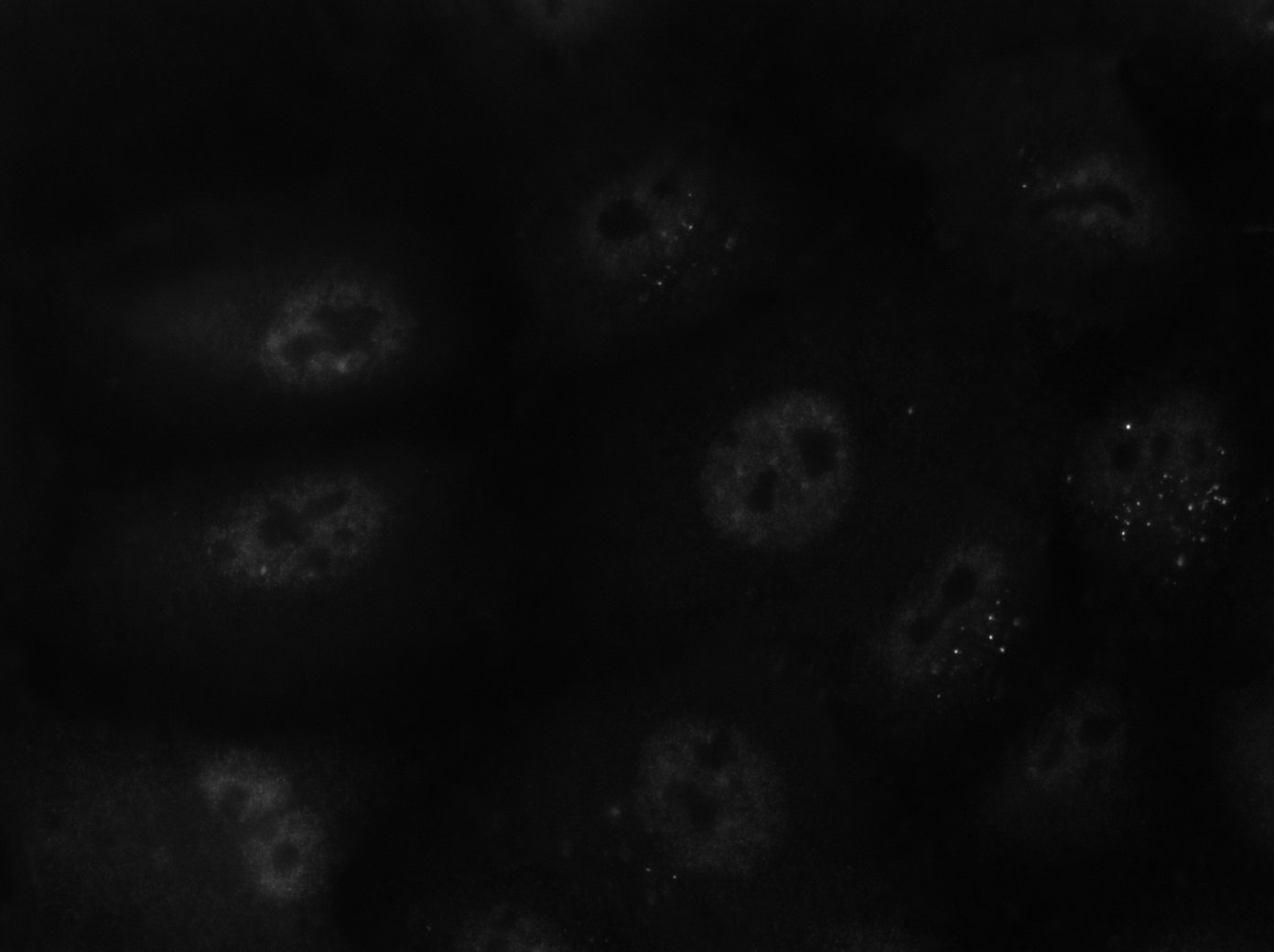

Supplement: Figure 5—figure supplement 2—source data 4. [file elife-72867-fig5-figsupp2-data4.zip › Figure5-figure_supplement_2-source data4/SmWT_SmB_488_60x_125_500ms_01.jpg]

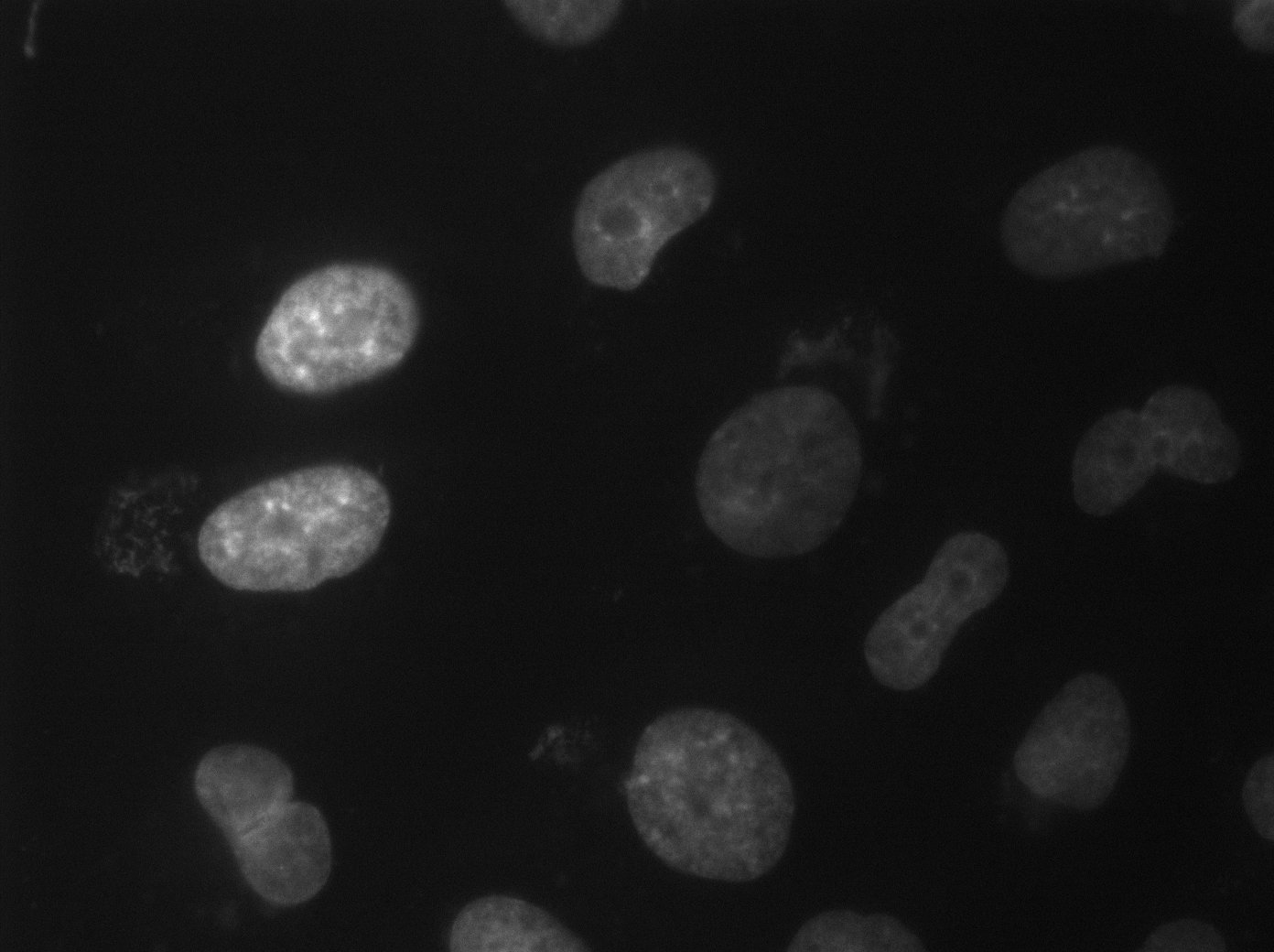

Supplement: Figure 5—figure supplement 2—source data 4. [file elife-72867-fig5-figsupp2-data4.zip › Figure5-figure_supplement_2-source data4/SmWT_SmB_DAPI_60x_125_500ms_01.jpg]

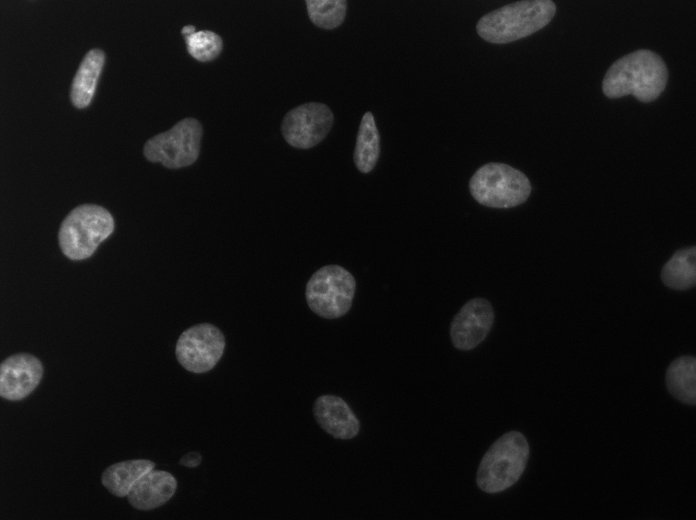

Supplement: Figure 5—figure supplement 2—source data 5. [file elife-72867-fig5-figsupp2-data5.zip › Figure5-figure_supplement_2-source data5/48H_DMSO_DAPI_01.jpg]

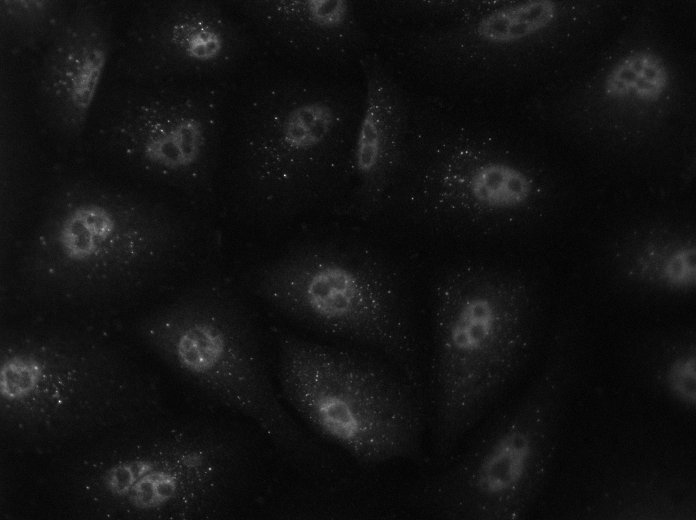

Supplement: Figure 5—figure supplement 2—source data 5. [file elife-72867-fig5-figsupp2-data5.zip › Figure5-figure_supplement_2-source data5/48H_DMSO_SNRPB_488_01.jpg]

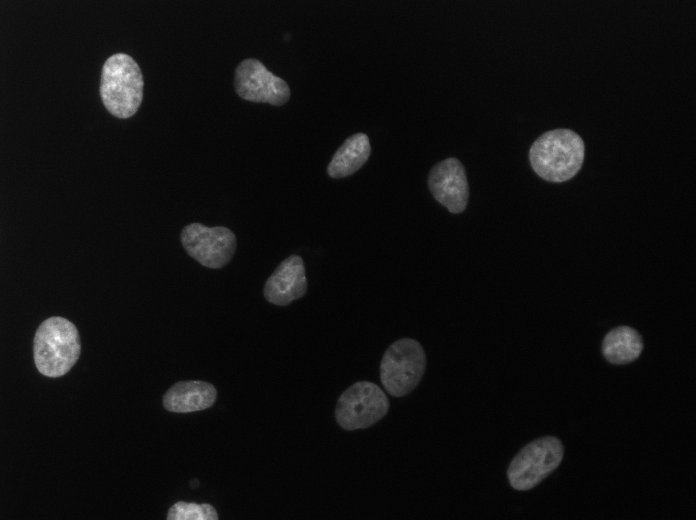

Supplement: Figure 5—figure supplement 2—source data 5. [file elife-72867-fig5-figsupp2-data5.zip › Figure5-figure_supplement_2-source data5/48H_GSK591_DAPI_02.jpg]

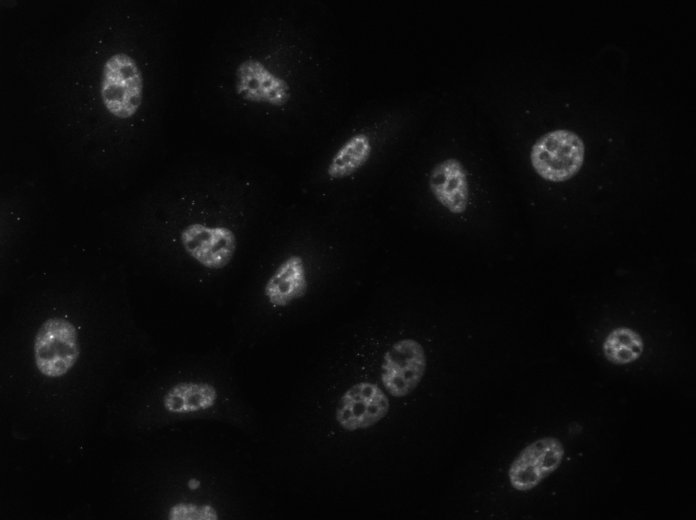

Supplement: Figure 5—figure supplement 2—source data 5. [file elife-72867-fig5-figsupp2-data5.zip › Figure5-figure_supplement_2-source data5/48H_GSK591_SNRPB_488_02.jpg]

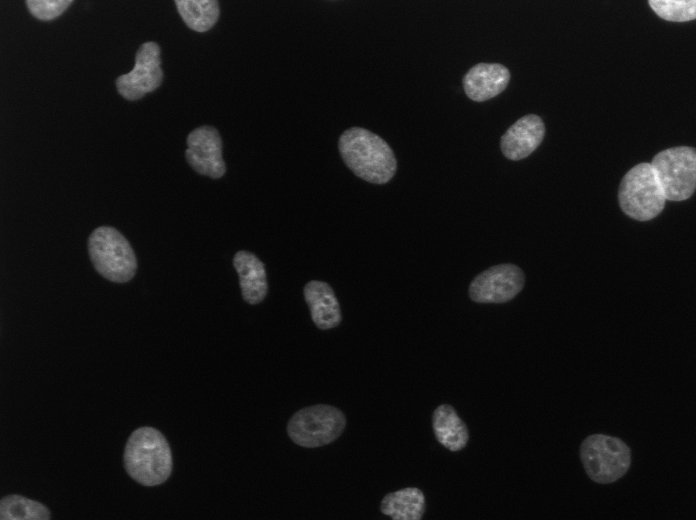

Supplement: Figure 5—figure supplement 2—source data 5. [file elife-72867-fig5-figsupp2-data5.zip › Figure5-figure_supplement_2-source data5/48H_MS023_DAPI_01.jpg]

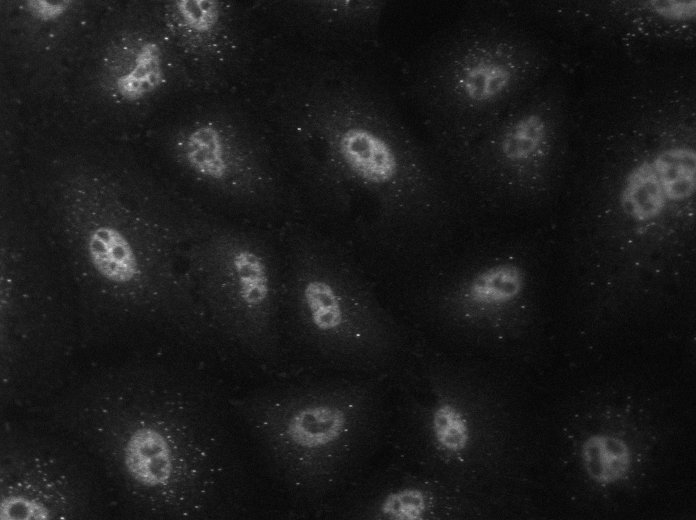

Supplement: Figure 5—figure supplement 2—source data 5. [file elife-72867-fig5-figsupp2-data5.zip › Figure5-figure_supplement_2-source data5/48H_MS023_SNRPB_488_01.jpg]

e

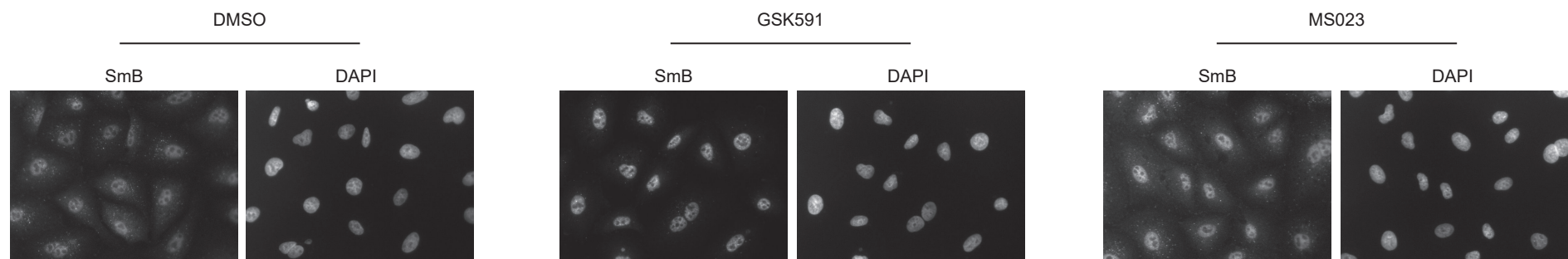

Supplement: Figure 5—figure supplement 2—source data 5. [file elife-72867-fig5-figsupp2-data5.zip › Figure5-figure_supplement_2-source data5/Figure5-figure_supplement_2-data5.pdf]

c

Chemiluminescence

Digital

Direct Blue 71 Membrane Stain

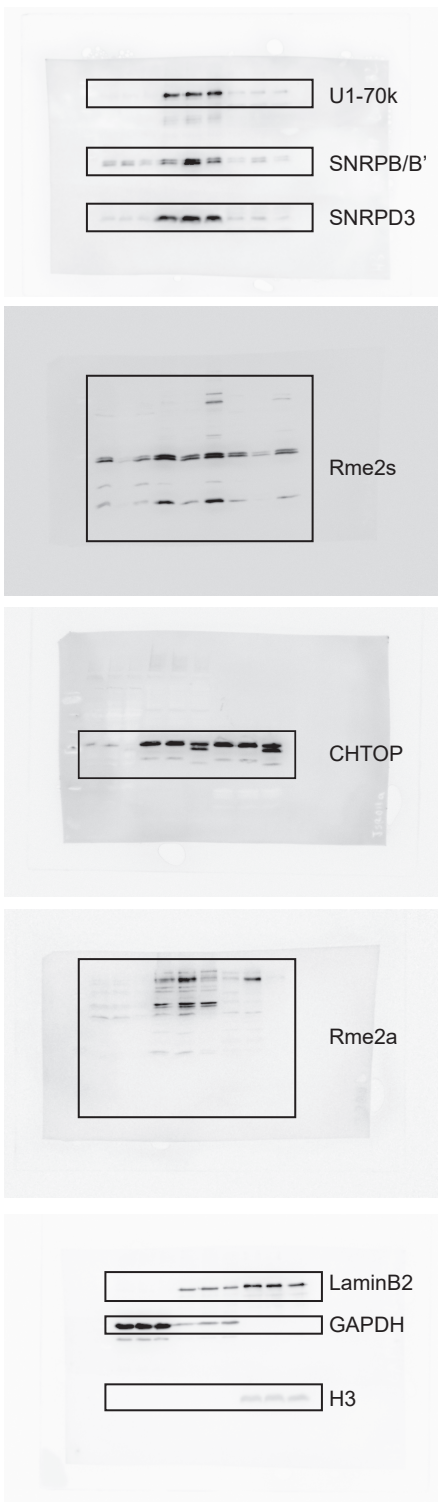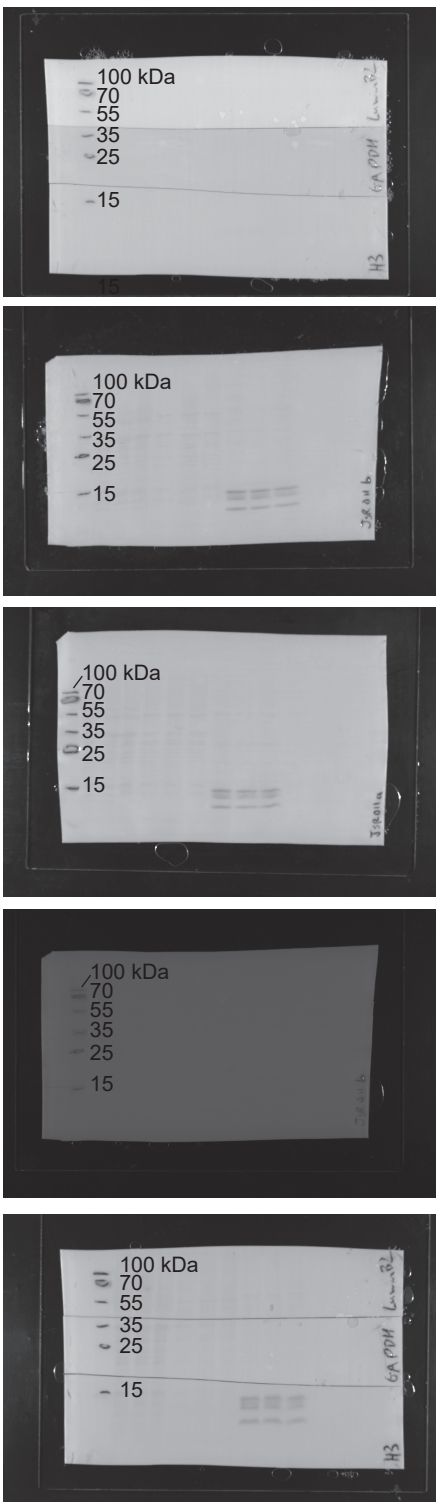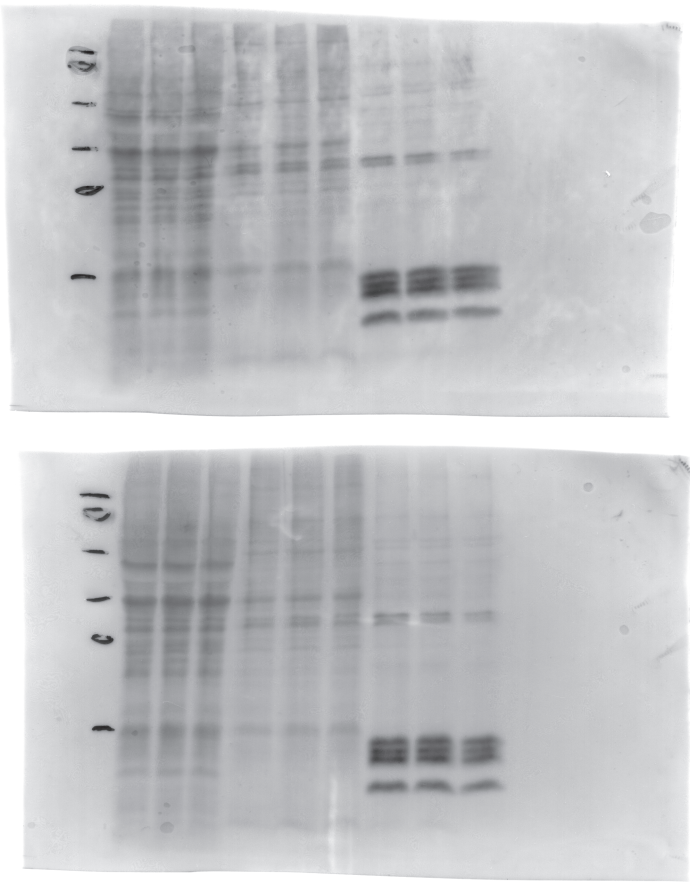

Supplement: Figure 6—source data 1. [file elife-72867-fig6-data1.zip › Figure6-source data1/Figure 6-source data 1.pdf]

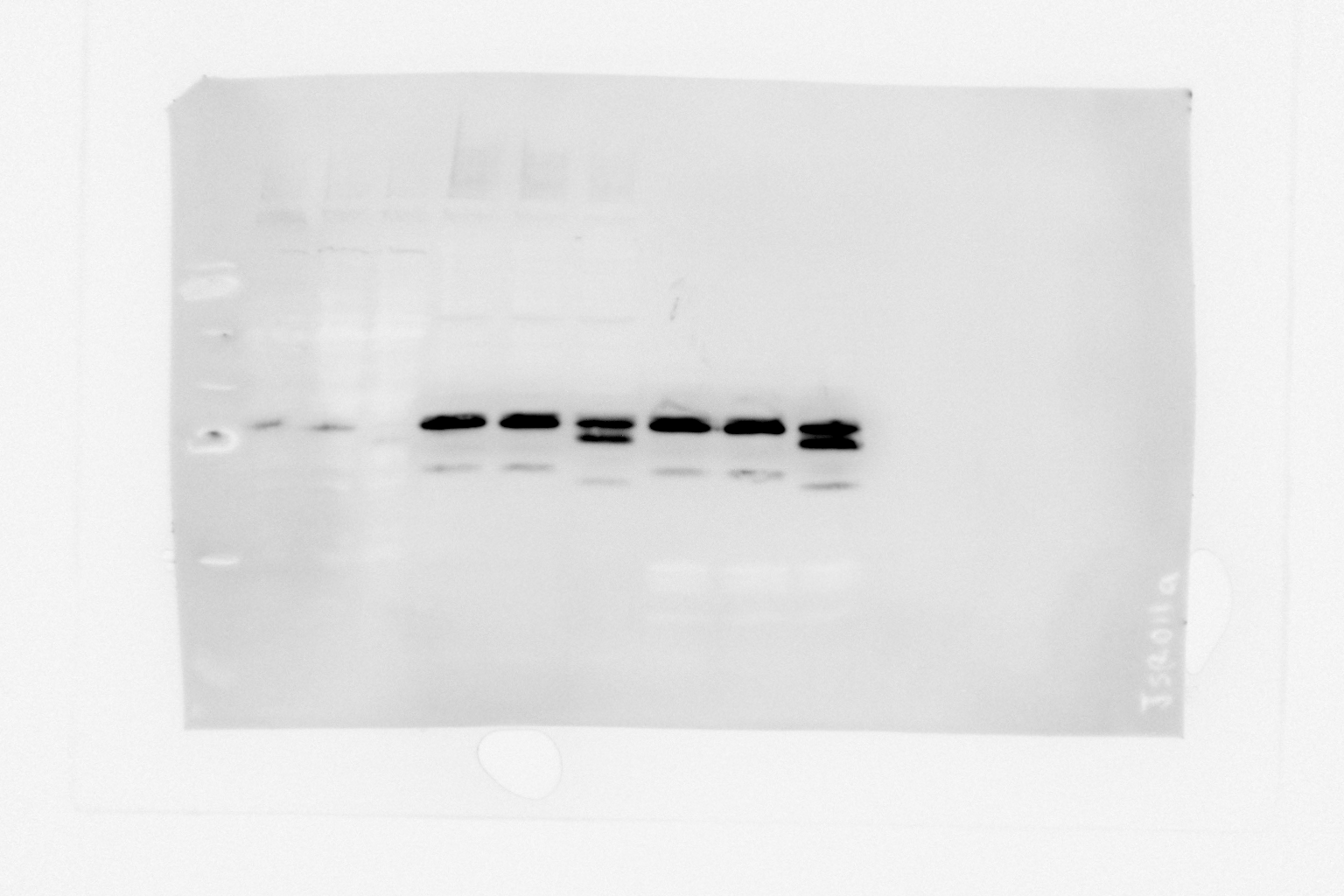

Supplement: Figure 6—source data 1. [file elife-72867-fig6-data1.zip › Figure6-source data1/FractionatedTreatments_DMSO-GSK591-MS023_CHTOP_1.2000_Standard_10sec_10.tif]

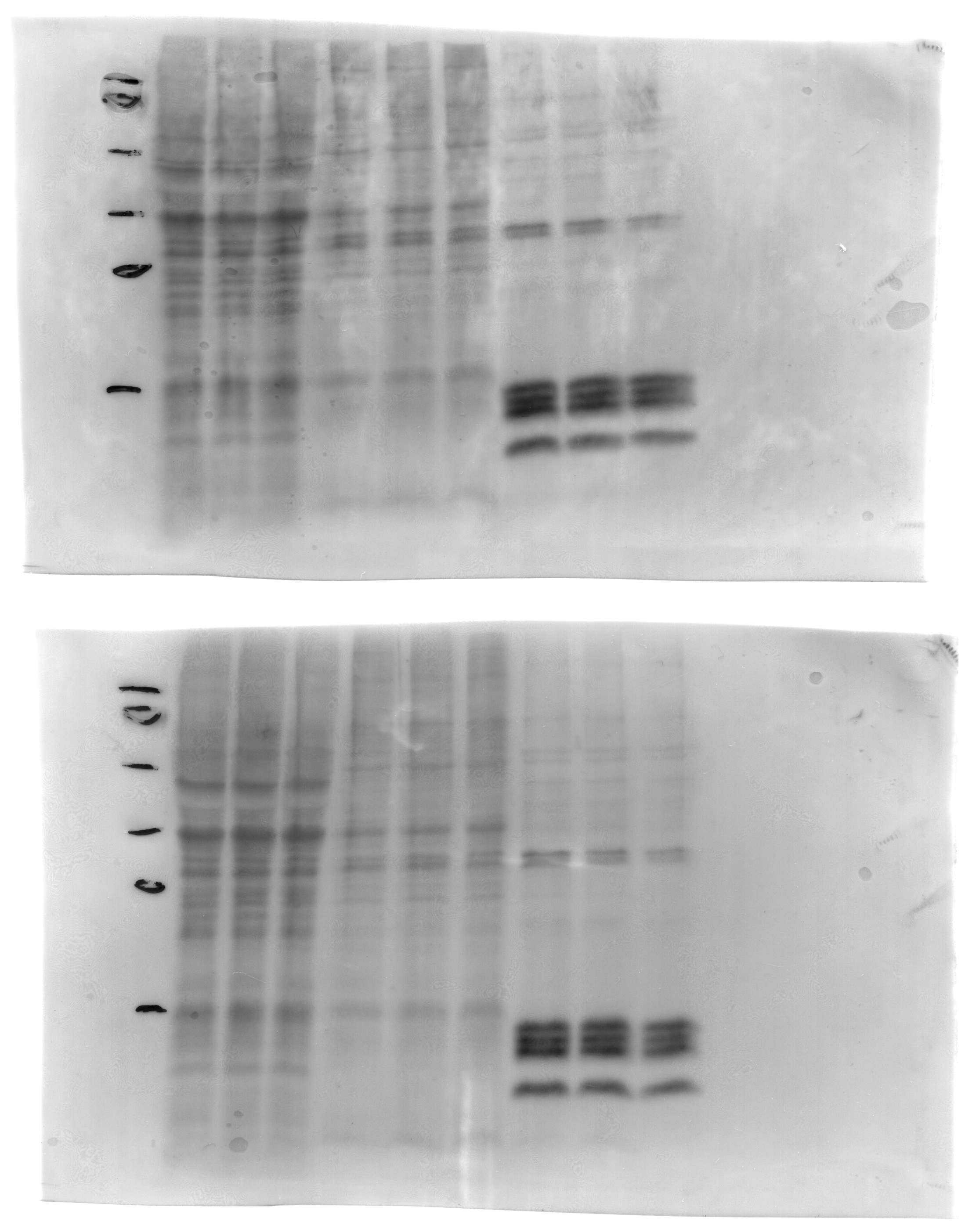

Supplement: Figure 6—source data 1. [file elife-72867-fig6-data1.zip › Figure6-source data1/FractionatedTreatments_DMSO-GSK591-MS023_DB71.tif]

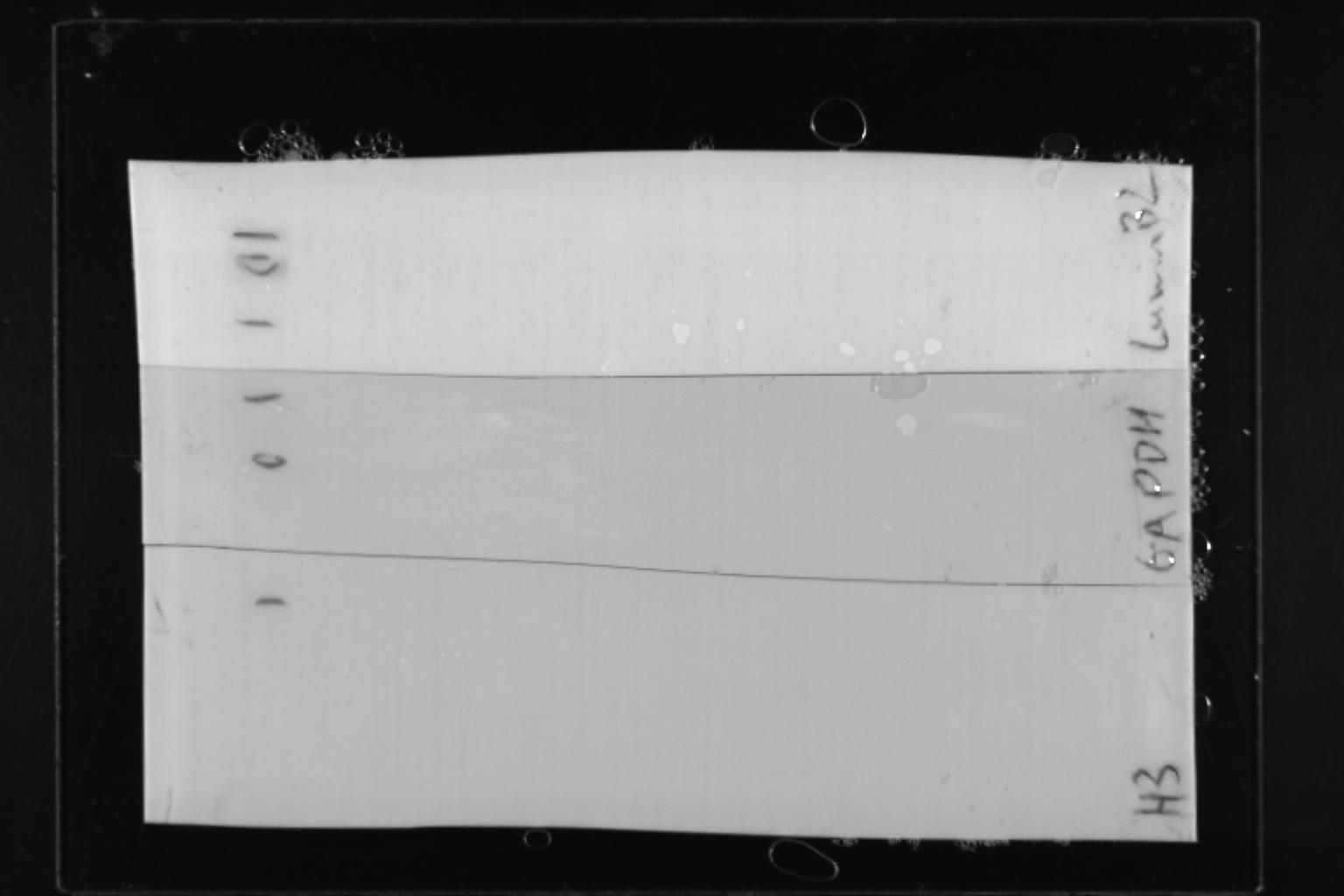

Supplement: Figure 6—source data 1. [file elife-72867-fig6-data1.zip › Figure6-source data1/FractionatedTreatments_DMSO-GSK591-MS023_High_digital.tif]

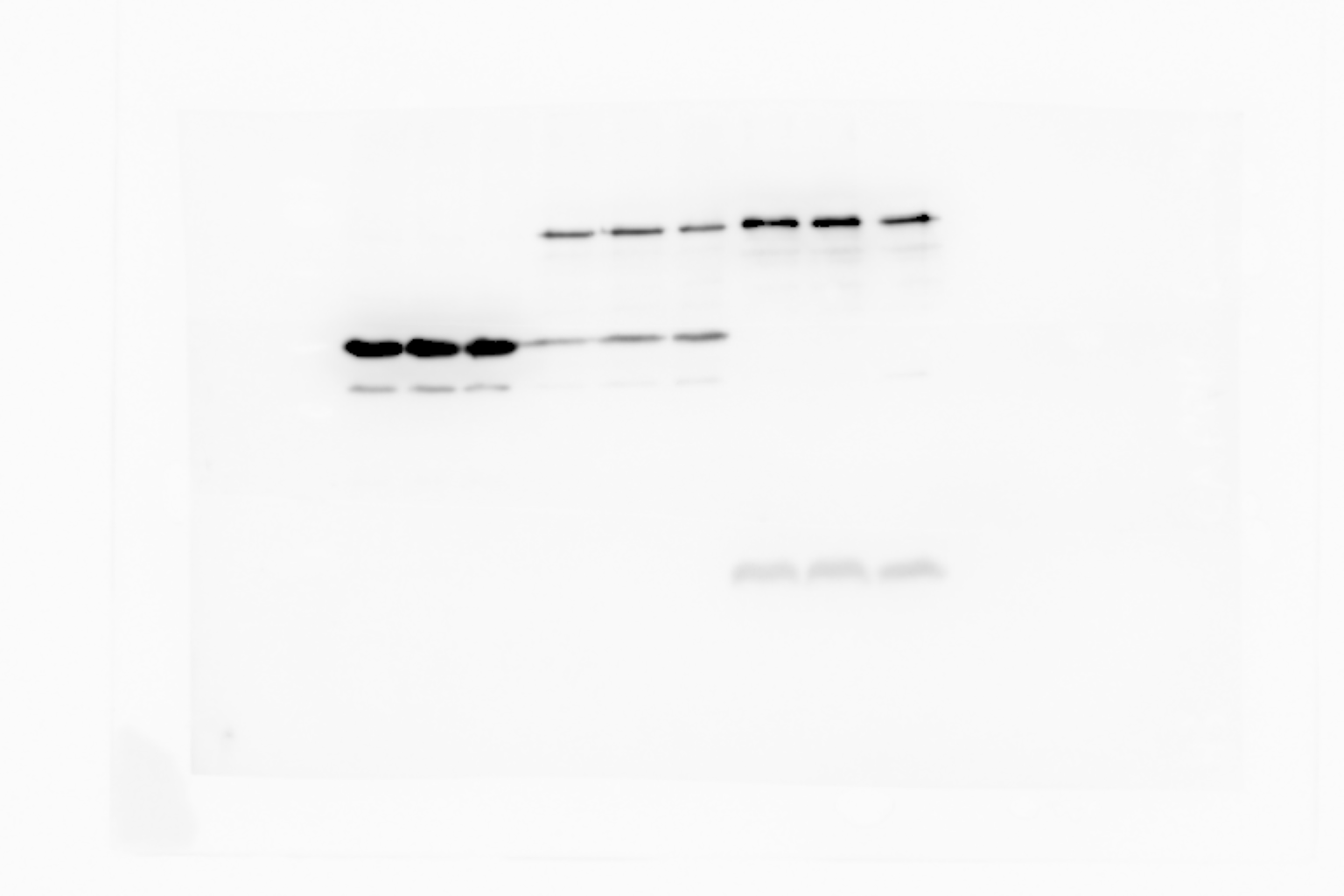

Supplement: Figure 6—source data 1. [file elife-72867-fig6-data1.zip › Figure6-source data1/FractionatedTreatments_DMSO-GSK591-MS023_LaminB2_GAPDH_H3_High_30sec_10.tif]

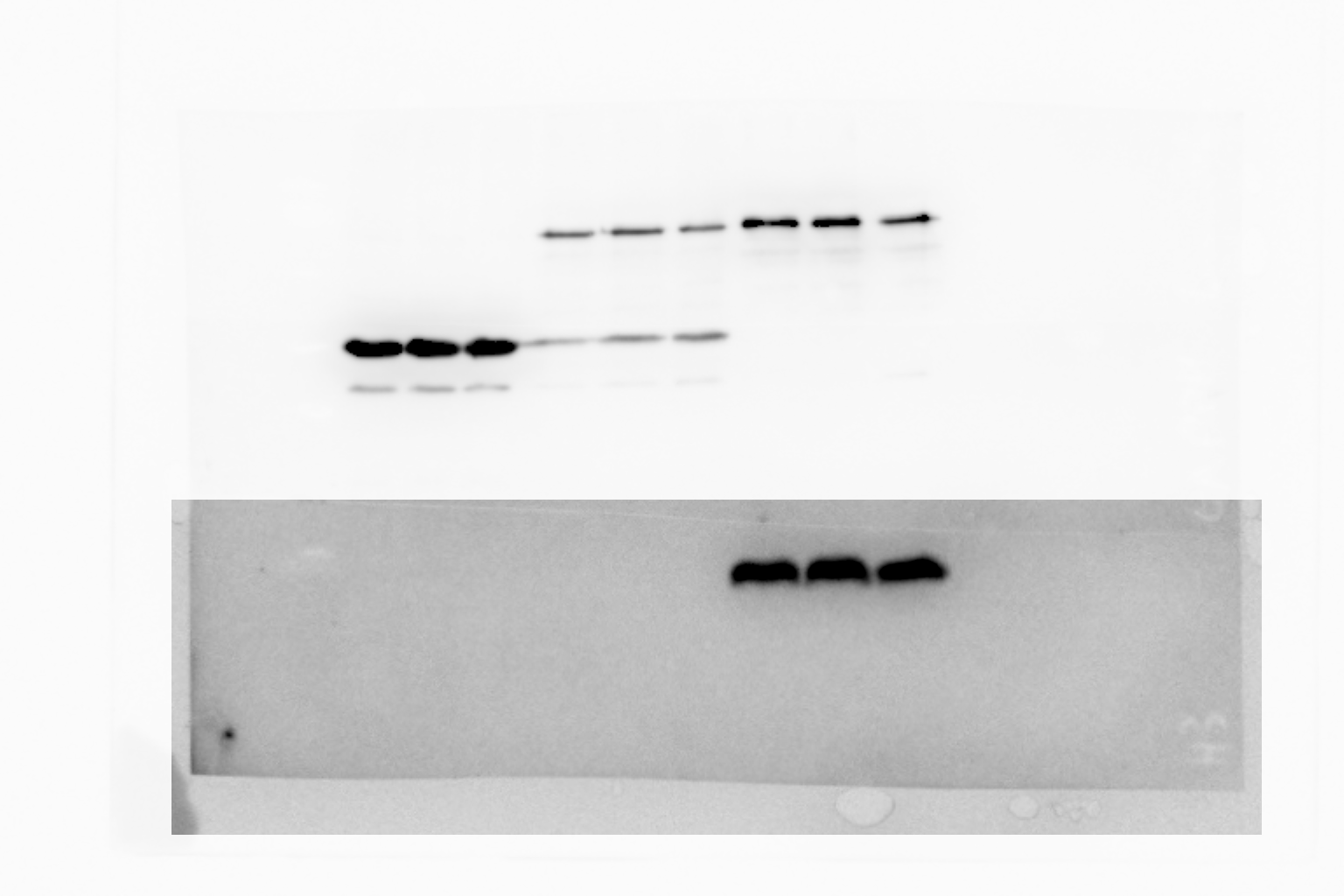

Supplement: Figure 6—source data 1. [file elife-72867-fig6-data1.zip › Figure6-source data1/FractionatedTreatments_DMSO-GSK591-MS023_LaminB2_GAPDH_H3_High_30sec_10_H3_HigherLevels.tif]

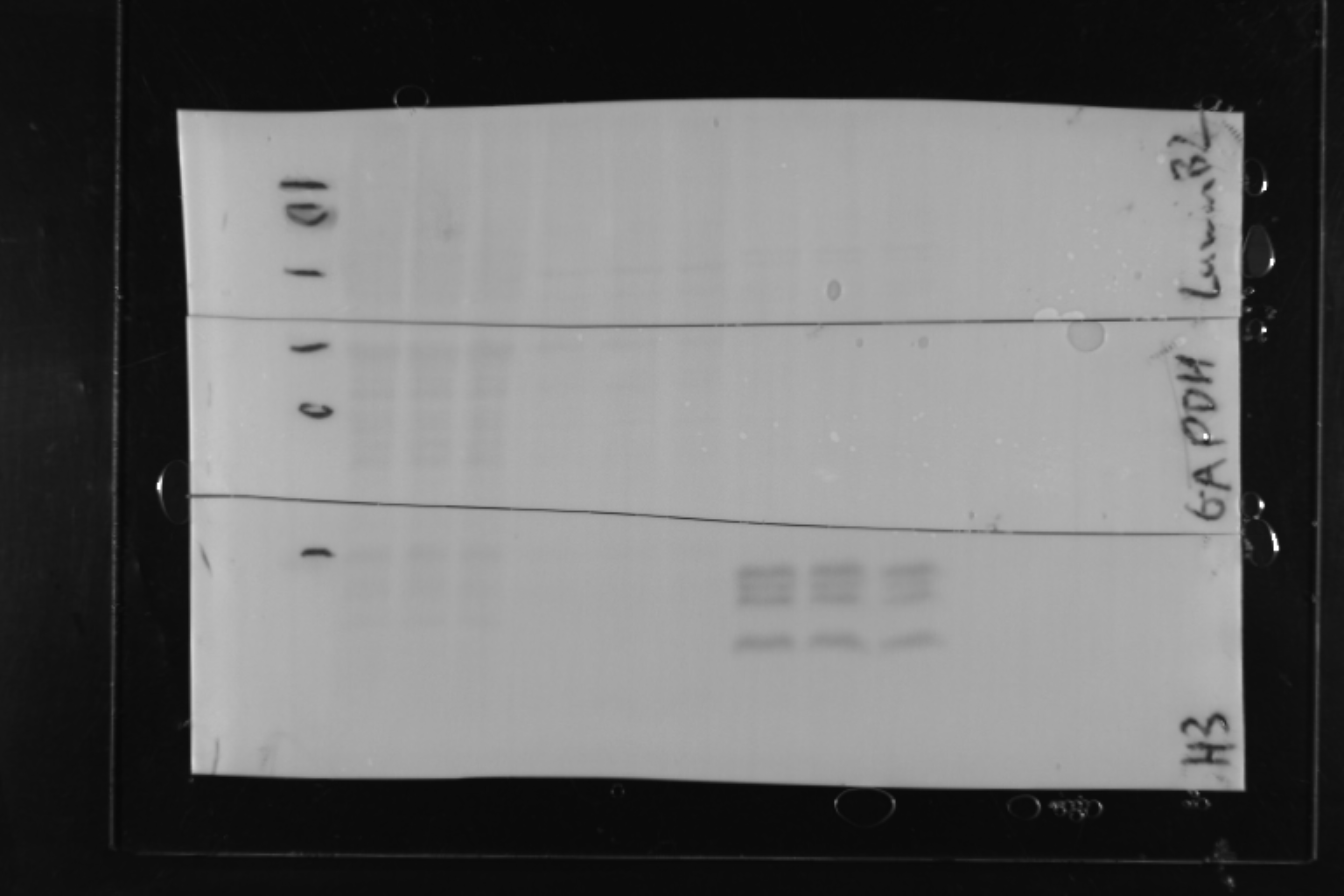

Supplement: Figure 6—source data 1. [file elife-72867-fig6-data1.zip › Figure6-source data1/FractionatedTreatments_DMSO-GSK591-MS023_LaminB2_GAPDH_H3_High_digital.tif]

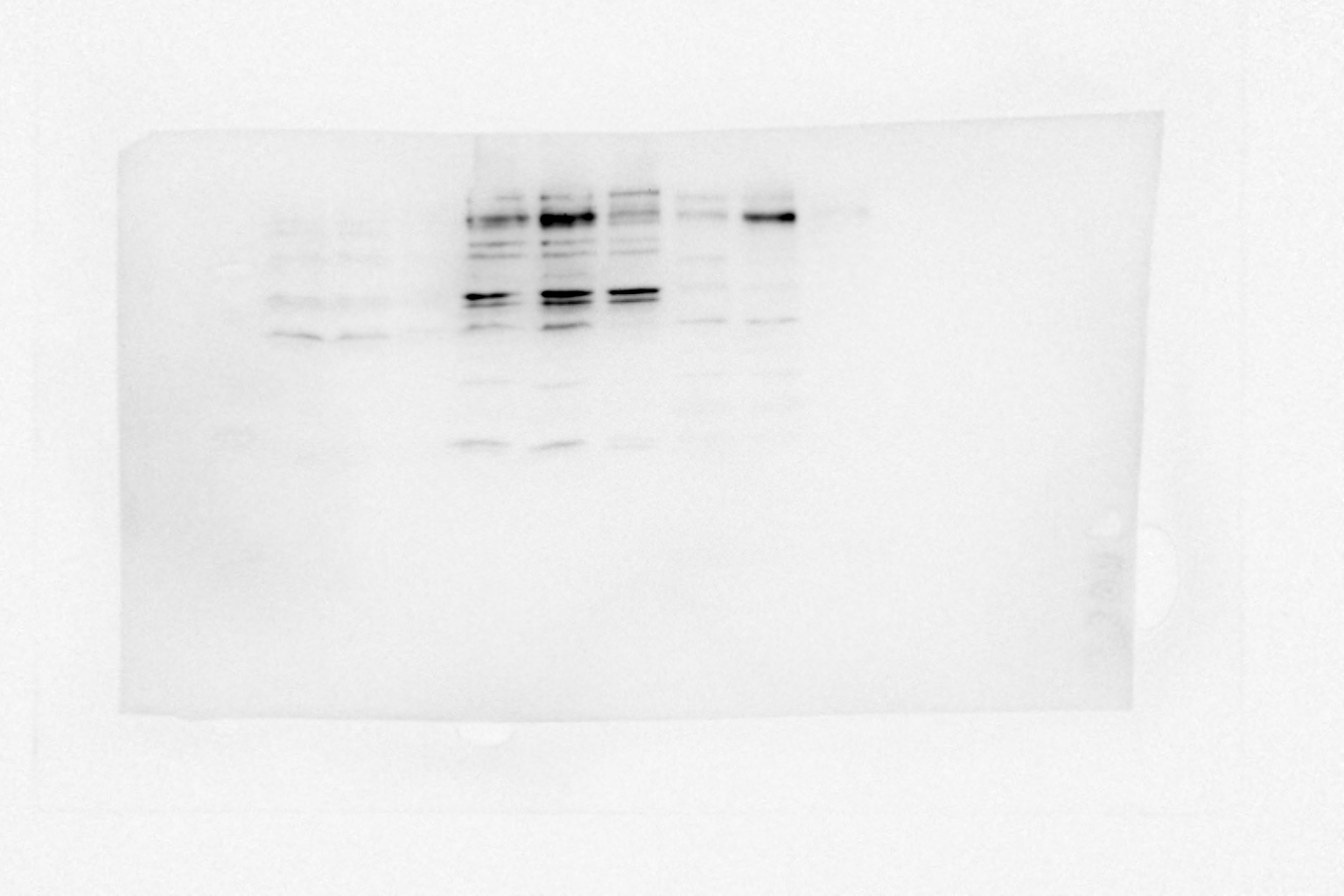

Supplement: Figure 6—source data 1. [file elife-72867-fig6-data1.zip › Figure6-source data1/FractionatedTreatments_DMSO-GSK591-MS023_Rme2a.tif]

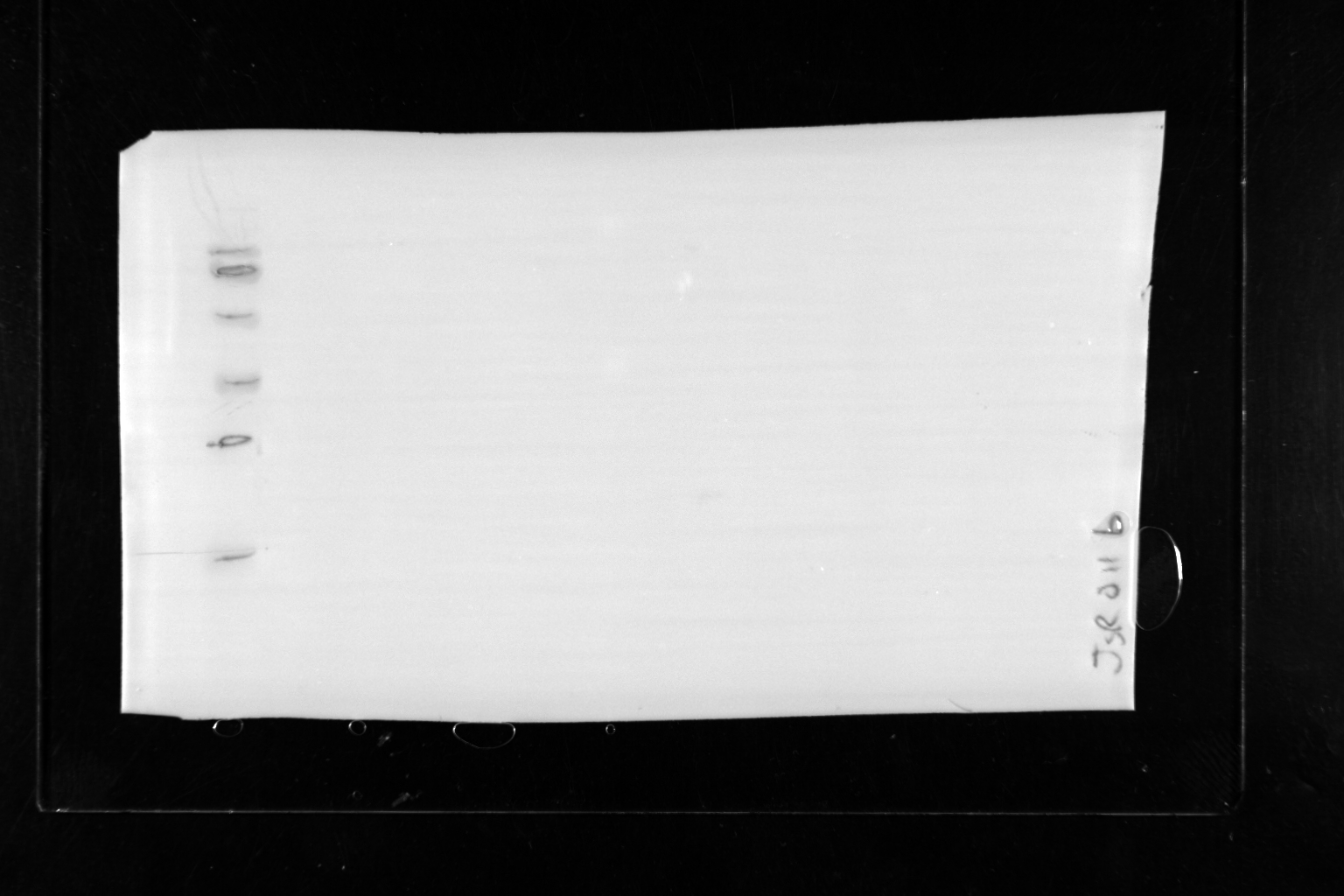

Supplement: Figure 6—source data 1. [file elife-72867-fig6-data1.zip › Figure6-source data1/FractionatedTreatments_DMSO-GSK591-MS023_Rme2a_High_digital.tif]

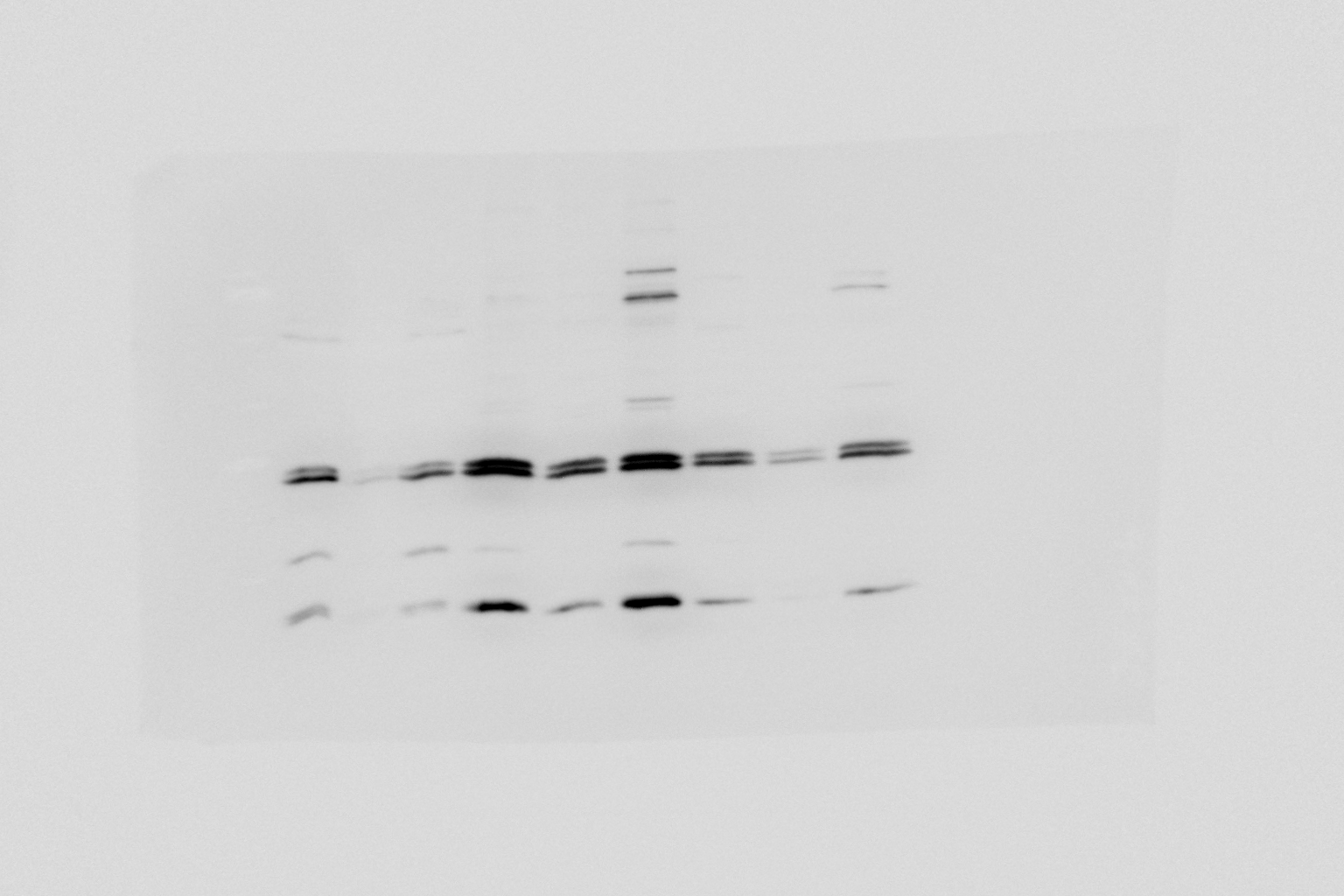

Supplement: Figure 6—source data 1. [file elife-72867-fig6-data1.zip › Figure6-source data1/FractionatedTreatments_DMSO-GSK591-MS023_Rme2s_1.2000_Standard_10sec_10.tif]

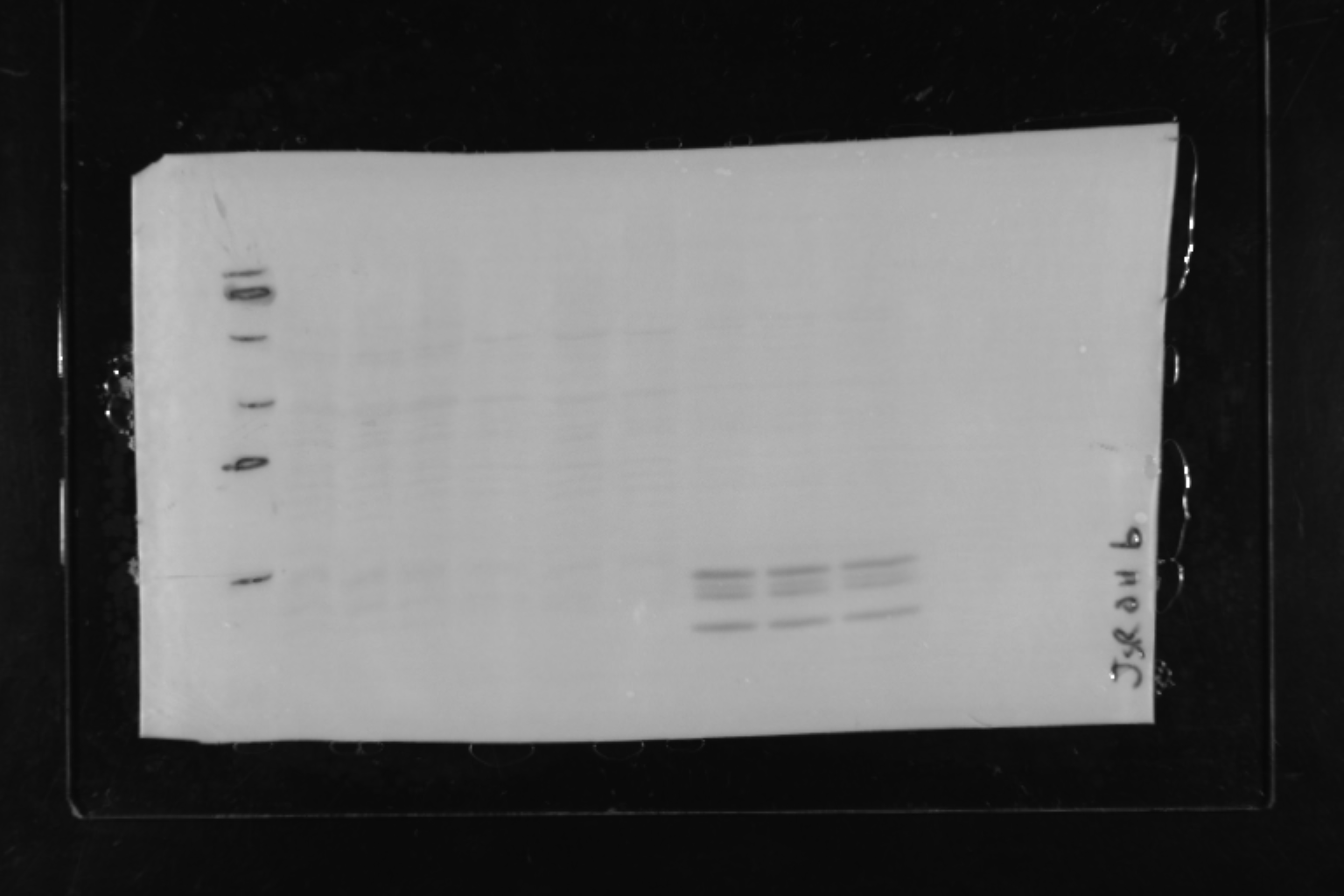

Supplement: Figure 6—source data 1. [file elife-72867-fig6-data1.zip › Figure6-source data1/FractionatedTreatments_DMSO-GSK591-MS023_Rme2s_digital.tif]

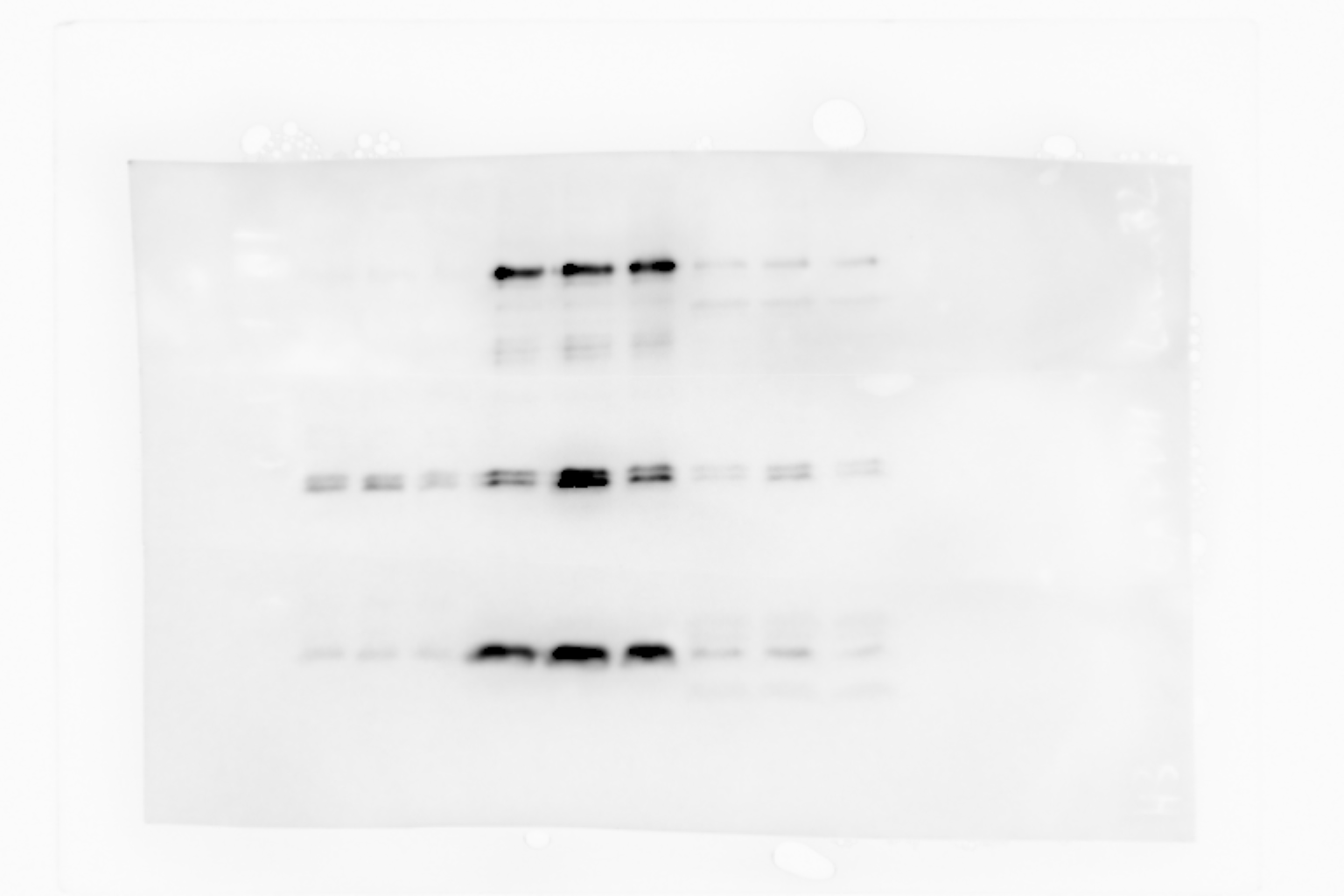

Supplement: Figure 6—source data 1. [file elife-72867-fig6-data1.zip › Figure6-source data1/FractionatedTreatments_DMSO-GSK591-MS023_U170k_SmB_SmD3_High_30sec_10.tif]

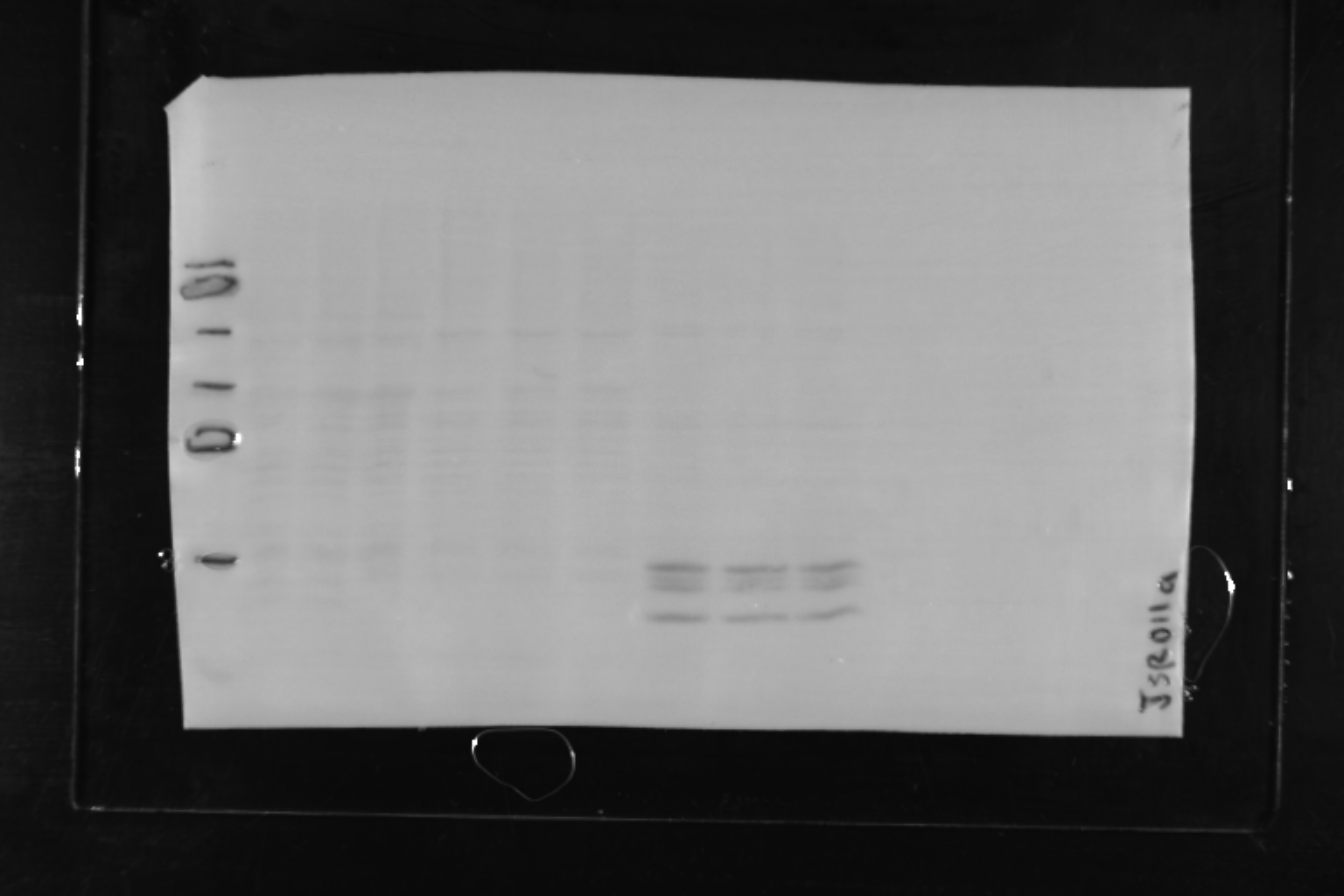

Supplement: Figure 6—source data 1. [file elife-72867-fig6-data1.zip › Figure6-source data1/FractionatedTreatment_CHTOP_1.2000_High_digital.tif]
